# A Consistent Approach to One Coordinate Pnictide Moieties M≡Pn^−^ Using PnH_2_^−^ Salts (M = Zr, Ti; Pn = P, As, Sb).

**DOI:** 10.1002/anie.202523745

**Published:** 2026-01-01

**Authors:** Matthew R. Mena, Mrinal Bhunia, Rishi T. Bhandari, Kevin Dollberg, Nick Michel, Alexandra M. Bacon, Michael R. Gau, Florian Weigend, Carsten von Hänisch, Daniel J. Mindiola

**Affiliations:** ^1^ Department of Chemistry University of Pennsylvania Philadelphia Pennsylvania 19104 USA; ^2^ Fachbereich Chemie and Marburg Center for Quantum Materials and Sustainable Technology (mar.quest) Philipps‐Universität Marburg Hans‐Meerwein‐Straße 4 Marburg 35043 Germany; ^3^ Institute for Quantum Materials and Technology Karlsruhe Institute of Technology Kaiserstr. 12 Karlsruhe 76131 Germany

**Keywords:** Arsenide, Atom transfer, Stibide, Titanium, Zirconium

## Abstract

Establishing a congruent approach to delivering a pnictide atom (Pn^3−^ = P, As, and Sb) to transition metal ions is a challenge, especially for the heavier congeners As and Sb, and when varying the transition metal. We showcase here a convenient route to molecular forms of one coordinate stibide ligands bound to Zr^IV^ and Ti^IV^ ions, represented by the discrete salts [K(L)][(PN)_2_M≡Sb] (M = Zr (**2**), Ti (**10**); PN^−^ = (N‐(2‐P^i^Pr_2_‐4‐methylphenyl)‐2,4,6‐Me_3_C_6_H_2_; L = 2,2,2‐Kryptofix or 18‐C‐6 crown‐ether/2THF), which were prepared via H_2_ extrusion from [K(18‐C‐6)(THF)SbH_2_] added to the Zr^IV^ cyclometallated‐hydride, [(PN)(PN’)Zr(H)] (**1**), and the Ti^II^ precursor [K(18‐C‐6)][(PN)_2_TiCl] (**8**) respectively. This strategy was extended to the lighter congeners Pn = As, P using [K(18‐C‐6)(THF)AsH_2_] (M = Zr (**3**)), and NaPH_2_ (M = Ti (**6**)). Structural and computational studies were applied to understand the bonding trends in the pnictide series, and the role of the metal ion.

## Graphical abstract

One coordinate pnictide (Pn^3−^ = P, As, and Sb) salts of Ti^IV^ and Zr^IV^ were prepared using PnH_2_^−^ as pnictogen atom transfer from suitable Ti^II^ and Zr^IV^ complexes through H_2_ extrusion. This strategy allows access to unprecedented 3d and 4d terminal stibides, alongside phosphide and arsenide analogues, enabling systematic comparisons across the Pn group and M ion. Crystallographic, spectroscopic, and computational studies reveal maximum covalency in the TiAs and ZrAs bonding.

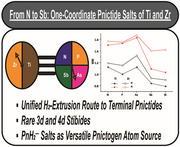

## Introduction

**Figure 1:**
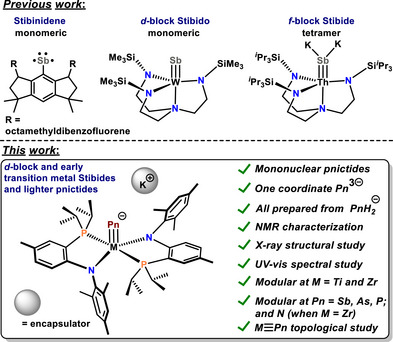
Top: Previous work showing examples of one coordinate Sb compounds (left); transition metal stibido (center) and actinide stibide salt (right). Bottom: This work showing terminal stibide of 3d and 4d transition metals and other pnictides along with their unique features.

Pnictogen‐atom transfer to a transition metal center is generally governed by the pnictogen (Pn = N, P, As, Sb, and Bi) and the transition metal complex accepting the atom. For instance, one‐coordinate N bound to a metal center can be installed using multiple reagents such as N_2_,^[^ ^1^ ^]^ N_3_^−^, and analogues,^[^ ^2^, ^3^, ^4^, ^5^, ^6^, ^7^, ^8^, ^9^, ^10^, ^11^, ^12^ ^]^ NH_2_^−^ and protected nitrides such as N{SiMe_3_}_2_^−^,^[^ ^8^, ^13^, ^14^, ^15^, ^16^ ^]^ as well as electrophilic metal nitrides among many other examples.^[^ ^17^, ^18^, ^19^ ^]^ In contrast, P‐atom transfer to a transition metal is limited to fewer reagents like P_4_,^[^ ^20^, ^21^, ^22^, ^23^, ^24^ ^]^ PCO^−^,^[^ ^25^, ^26^, ^27^, ^28^, ^29^ ^]^ PH_2_^−^ and protected phosphides,^[^ ^30^, ^31^, ^32^, ^33^, ^34^ ^]^ and anthracene extrusion using a chloro‐dibenzo‐7‐λ^3^‐phosphanorbornadiene.^[^ ^35^, ^36^, ^37^ ^]^ The list becomes narrower for As‐atom transfer reagents such as metastable As_4_,^[^ ^38^, ^39^, ^40^, ^41^ ^]^ AsCO^−^,^[^ ^10^, ^26^, ^42^ ^]^ AsH_2_^−^ and protected arsenides.^[^ ^34^, ^43^, ^44^, ^45^, ^46^, ^47^, ^48^, ^49^ ^]^ This inventory of reagents significantly dwindles for the heavier congeners Sb, and Bi, with the latter having no reported known one coordinate bismuthido (Bi^3−^) complexes. In the case of Sb, only Li[Sb(H)CH(SiMe_3_)_2_],^[^ ^50^ ^]^ and more recently, SbH_2_^−^ or analogs,^[^ ^51^, ^52^, ^53^, ^54^, ^55^ ^]^ have been shown to deliver an Sb‐atom to a metal center. In conjunction to one coordinate Sb,^[^ ^56^ ^]^ complexes containing a multiple bond to Sb was restricted, until recently, to Scheer's^[^ ^50^ ^]^ (Tren^S^)W≡Sb (Tren^S^ = N{CH_2_CH_2_NSiMe_3_}_3_ (Figure 1). However, the introduction of Sb‐ and Bi‐atom transfer reagents such as K(18‐C‐6)(THF)SbH_2_ and KBiMe_2_,^[^ ^55^ ^]^ has enabled the isolation of an *f*‐block stibide,^[^ ^53^ ^]^ [{(Tren^TIPS^)Th≡SbK_2_}_4_] (Tren^TIPS^ = N{CH_2_CH_2_NSi(^i^Pr)_3_}_3_, Figure 1, Liddle and coworkers) as well as metallodipnictenes, {(PNP)M–Pn═Pn–M(PNP)} (PNP = N(CHCHP*^t^* Bu_2_)_2_; M = Pd^II^, Pt^II^; Pn = P, As, Sb, Bi) complexes via transient metallopnictinidene diradical intermediates,^[^ ^55^ ^]^ {(PNP)M–Pn} (Pn = P, As, Sb, Schneider and coworkers).

Given the advent of a Sb^3−^ transfer reagent, we sought to find a congruent route to a series of isoelectronic terminal pnictides spanning N to Sb using synthetically accessible salts having the PnH_2_^−^ group. In addition, we wanted to extend this methodology to other early‐transition metals therefore allowing us to compare the M≡Pn bonding by switching systematically the Pn, but also M. Herein, we describe the use of PnH_2_^−^ reagents (where Pn = P, As, and Sb) as reliable Pn^3−^ atom sources via H_2_ extrusion, when treated with Zr^IV^ and Ti^II^ precursors to yield the pnictide series [K(L)][(PN)_2_M≡Pn] (M = Zr and Ti; L = cryptand or crown‐ether; Pn = P, As, and Sb). In addition to their structural elucidation, these one coordinate pnictides series were probed computationally to help elucidate their bonding topology.

**Scheme 1:**
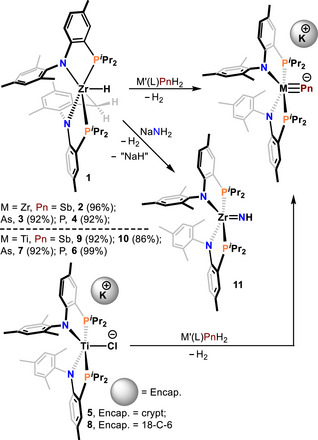
Top reaction shows the synthesis of one coordinate pnictides of Zr^IV^ using precursor **1**. In the case of NaNH_2_, the parent Zr^IV^ imide, **11** is obtained. The bottom reaction shows the Ti^IV^ pnictides stemming from the Ti^II^ complexes **5** and **8** (bottom left).

**Figure 2:**
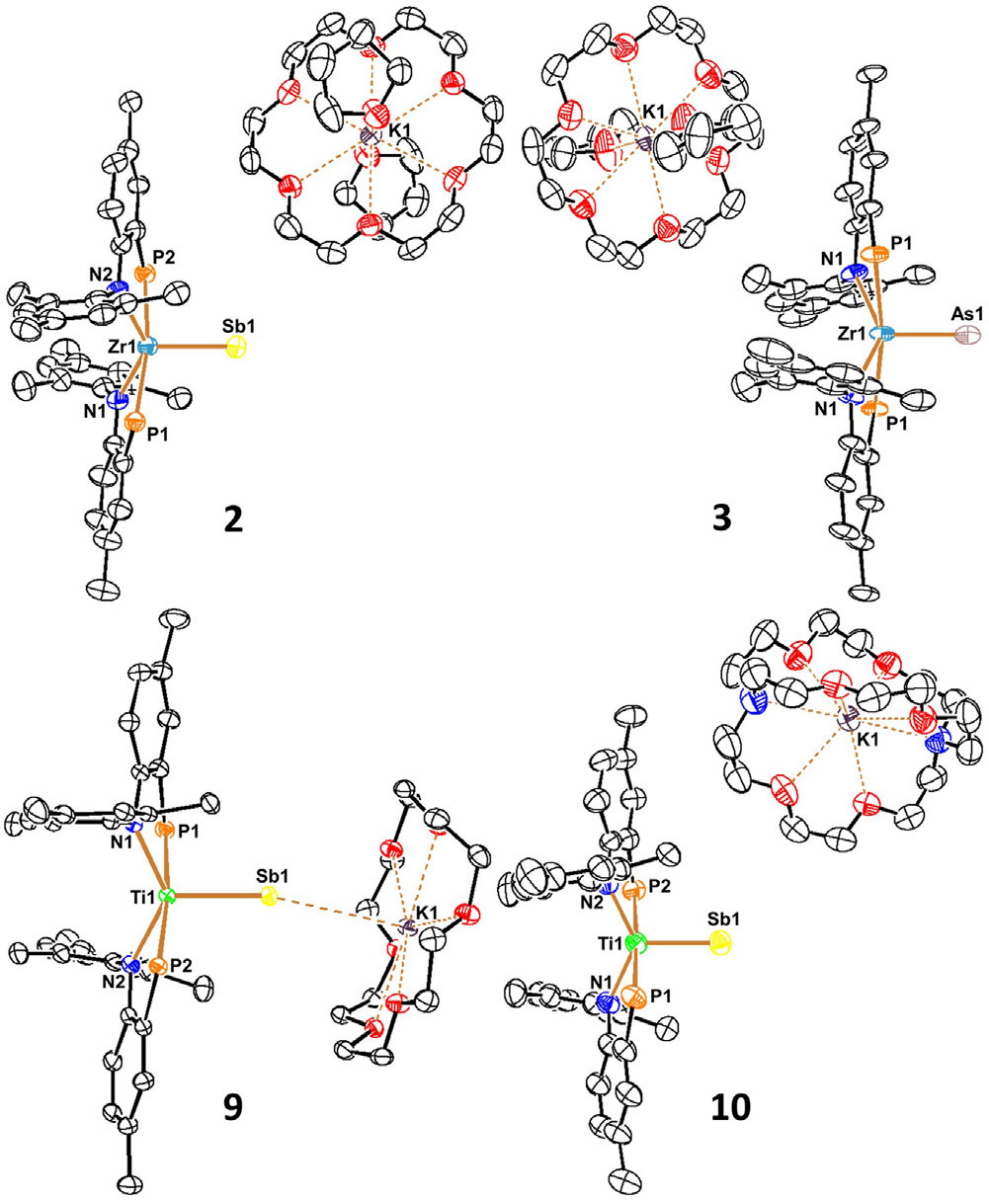
Molecular structures of Zr‐stibide (**2**), and arsenide (**3**), along with Ti‐stibide complexes **9** and **10** with thermal ellipsoids at 50% probability level and hydrogen atoms, cocrystallized solvents and ^*i*^Pr groups on P atom of PN‐ligands are omitted for clarity.

Recently, our group found a route to terminal phosphides of Zr^IV^, [K(L)][(PN)_2_Zr≡P] (**4**), by treatment of NaPH_2_ with an encapsulator L (L = 18‐crown‐6 ether, 18‐C‐6/2THF; crypt = 2,2,2‐Kryptofix) and the cyclometallated‐hydride [(PN)(PN’)Zr(H)] (**1**) (PN’^2−^ = (N‐(2‐P*^i^* Pr_2_‐4‐methylphenyl)‐2‐CH_2_‐4,6‐Me_2_C_6_H_2_).^[^ ^32^ ^]^ By analogy, one would anticipate that the heavier analogues AsH_2_^−^ and SbH_2_^−^, reported by Hänisch and coworkers,^[^ ^43^, ^52^ ^]^ should render access to the respective arsenide and stibide analogues. Accordingly, treatment of **1** with K(18‐C‐6)(THF)PnH_2_ (Pn = Sb, As) in benzene‐*d_6_* led to an immediate color change from orange to greyish‐brown (Sb) and magenta pink (As) (Scheme 1). Inspection of ^1^H NMR spectral data showed a new diamagnetic and *C_2v_* symmetric compound along with H_2_, whereas the ^31^P{^1^H} NMR spectra evinced a single resonance for the symmetrically related PN ligands at 46.5 ppm (Sb) and 31.2 ppm (As). Single crystals obtained from each worked‐up reaction revealed a 5‐coordinate Zr^IV^ center containing an unprecedented terminal stibide, [K(18‐C‐6)(THF)_2_][(PN)_2_Zr≡Sb] (**2**) and arsenide, [K(18‐C‐6)(THF)_2_][(PN)_2_Zr≡As] (**3**), via a single crystal X‐ray diffraction study (scXRD). Their solid‐state structures show no Pn•••K interactions (7.48638(17) Å, **2**; 7.44804(5) Å, **3**) (Figure 2) while the [Zr≡Sb]^−^ and [Zr≡As]^−^ were short at 2.6303(7) and 2.3951(8) Å, respectively. The Pauling equation^[^ ^57^ ^]^ corrected by the Schomaker–Stevenson coefficient^[^ ^61^ ^]^ (Table 1) arguably suggests **2** to possess more double bond character (Zr≡Sb^−^ ↔ Zr = Sb^−^), whereas **3** is intermediate between double and triple bond character.^[^ ^57^, ^58^, ^59^, ^60^, ^61^ ^]^ The [Zr≡Sb]^−^ bond length in **2** lies between other crystallographically characterized stibidos reported by Scheer (stibido, [W≡Sb]; 2.5255(17) Å) and Liddle (stibide [Th≡Sb^2−^], 2.823(16)–3.269(19) Å) which correlates well with their ionic radii {W^6+^(0.60 Å), Zr^4+^(0.72 Å), and Th^4+^(1.05 Å)}.^[^ ^50^, ^53^, ^62^ ^]^ The [Zr≡As]^−^ motif in **3** also joins a scant class of structurally characterized arsenidos.^[^ ^10^, ^26^, ^30^, ^42^, ^46^, ^49^ ^]^ In addition, the slightly elongated bond length of 2.3951(8) Å is consistent with other 4d metal arsenides (Nb, 2.3078(5) Å; Mo, 2.252(3), 2.2248(5) Å)) due to decreasing metal size across the row.^[^ ^40^, ^41^ ^]^

**Table 1:** Salient metrical parameters^a^ and ^31^P NMR chemical shifts of Zr‐ and Ti‐pnictides.

M≡Pn		N^3−^	P^3−^	As^3−^	Sb^3−^
**[Zr]≡Pn/ [Ti]≡Pn** **bond distances** **(Å); [Zr] = (PN)_2_Zr, [Ti] = (PN)_2_Ti**	**X‐ray** ^a)^	1.822(2)^11^/ 1.719(3)^61^	2.298(17)^32^/ 2.195(2)^33^	2.3951(8)/ 2.2661(5)^42^	2.6303(7)/ 2.5118(9)
	**Pauli calc**.^b)^	1.613/1.500	2.098/1.981	2.236/2.114	2.466/2.340
	**PBE** ^c)^	1.829/1.670	2.347/2.191	2.441/2.294	2.670/2.524
**^31^P{^1^H} (*δ*) [(*P*N)_2_M≡Pn]** ^d)^ **^,^** ^e)^ **;** **M = Zr/Ti**	**Expt**.	12.2/6.4	24.1/28.4	31.2/30.6	46.5/58.5
	**1c‐PBE**	8.1/9.2	14.7/21.6	16.2/23.4	17.5/25.4
	**2c‐PBE**	9.4/5.2	19.3/20.8	26.5/30.9	40.9/56.9

^a)^ Metrical parameters denote bond metrics obtained from scXRD studies of discrete salts unless otherwise noted.

^b)^ Calculations performed using the Pauling equation corrected by the Schomaker–Stevenson coefficient.

^c)^ Calculations performed using PBE/dhf‐TZVP/D3‐BJ/COSMO.

^d)^ All NMR spectra for Zr compounds were collected in benzene‐*d*_6_.

^e)^ All NMR spectra for Ti compounds were collected in THF‐*d*_8_.

Unlike Zr^IV^ where the use of two intramolecular bases facilitates sequential deprotonation of the PnH_2_^−^ in generating the Pn^3−^ ligand; our use of various low‐valent Ti^II^ fragments has been demonstrated to promote deazotation (N_3_^−^) and decarbonylation (AsCO^−^) to obtain one coordinate N^3−^ or As^3−^ ligand, respectively.^[^ ^42^, ^63^, ^64^ ^]^ In the case of P^3−^, a different approach was required using the Ti^II^ complex, [K(crypt)][(PN)_2_TiCl] (**5**) with NaP(SiMe_3_)_2_, followed by oxidative deprotection with XeF_2_ to form the phosphide salt [K(crypt)][(PN)_2_Ti≡P] (**6**).^[^ ^33^ ^]^ It was surmised that a more convenient and scalable route could be achieved directly with PH_2_^−^ and taking advantage of the weaker bond dissociation enthalpies (BDE, kcal/mol) for P−H (74.2) versus P−Si (86.8).^[^ ^65^, ^66^ ^]^ Gratifyingly, treatment of 1 equiv. NaPH_2_ with equimolar **5** ^42^ in THF at 60 °C for 30 min resulted in quantitative formation of **6**.^[^ ^67^ ^]^ Complex **6** could be spectroscopically confirmed via the highly downfield and distinguishable broadened resonance for the [Ti≡P]^−^ in the ^31^P{^1^H} NMR spectrum at 1446 ppm (Figure S24). Given the decreasing BDE of Pn‐H (Pn‐H; Pn = P > As > Sb)^[^ ^65^, ^67^ ^]^ our efforts were directed toward extending this protocol to the heavier analogs, including an unknown 3d stibide moiety, [Ti≡Sb]^−^. Moreover, addition of K(18‐C‐6)(THF)AsH_2_ to **5** in benzene‐*d_6_* over 10 min at 25 °C also led to rapid color change from brown‐red to pink‐red, and work‐up of the reaction mixture resulted in isolation of the corresponding arsenide [K(crypt)][(PN)_2_Ti≡As] (**7**) in 92% yield, which was confirmed spectroscopically by comparison to independently prepared complex (Figures S25,S26).^[^ ^68^ ^]^ Performing the same reaction of **5** with 1 equiv. K(18‐C‐6)(THF)SbH_2_ in benzene‐*d_6_* resulted in a rapid color change from brown‐red to dark green solution over 5 min at 25 °C. Unfortunately, monitoring the reaction mixture by ^1^H and ^31^P NMR spectra revealed incomplete consumption of **5** along with a new diamagnetic complex and H_2_ (4.47 ppm). The ^31^P{^1^H} NMR spectrum showed two new resonances, a major product at 58 ppm along with a minor species at 48 ppm (Figure S27). Despite optimization attempts, separation of the new species formed in the mixture was always marred with unreacted **5**.^[^ ^69^ ^]^ To overcome this, we explored a more soluble “Ti^II^” synthon by replacing the cryptand in **5** with crown ether 18‐C‐6. Accordingly, treating [(PN)_2_TiCl]^[^ ^63^ ^]^ with KC_8_ and 18‐C‐6 in toluene at 25 °C over 12 h resulted in the isolation of green and paramagnetic [K(18‐C‐6)][(PN)_2_TiCl] (**8**) in 67% yield (*µ*_eff_ = 2.61(1) *µ*_B,_ 25 °C, benzene‐*d_6_*). A scXRD study revealed similar bonding metrics to that of **5** but with a longer Ti–Cl distance of 2.4687(10) Å [**5**; 2.435(1) Å] and a closer Cl…K(18‐C‐6) contact of 6.018(6) Å [**5**; 6.976(1) Å].^[^ ^68^ ^]^ With precursor **8** in hand, addition of equimolar K(18‐C‐6)(THF)SbH_2_ resulted in quantitative formation of the stibide‐ate complex [(PN)_2_Ti≡Sb{K(18‐C‐6)}] (**9**), (Figure 2).^[^ ^68^ ^]^ NMR spectral features (^1^H, ^13^C, and ^31^P) along with scXRD confirmed its identity, with the most diagnostic feature being the short Ti≡Sb of 2.5181(4) Å as well as close contact pairing interaction of the terminal stibide and counter cation (Sb…K; 3.7289(5) Å) (Figure S59). To generate the discrete salt, the addition of cryptand to a toluene/THF solution of **9** quantitatively afforded the one coordinate stibide [K(crypt)][(PN)_2_Ti≡Sb] (**10**) as a green solid, which was also confirmed crystallographically (Figure 2) and spectroscopically.^[^ ^68^ ^]^

The scXRD of **10** (Figure 2) revealed a *τ*_5_ = 0.69 and terminal stibide ligand (Ti≡Sb; 2.5118(9) Å, Table 1), with no close contact pairing (Sb…K: 7.7309(14) Å). Akin to stibide **2**, the experimentally determined Ti≡Sb bond length of 2.5118(9) Å aligns closer to the calculated value for a double bond (2.502 Å) than that of a triple bond (2.340 Å, Table 1). This interpretation is further implied by comparison with the only crystallographically characterized 3d‐metal bridging stibinidene [{Cp*(CO)_2_Cr}_2_Sb][GaCl_4_] reported by Imhof and coworkers (Cr; 2.378(2), 2.396(2) Å).^[^ ^70^ ^]^

Considering previous findings in our group of using protected N atom sources, N(SiMe_3_)_2_^−^, to access imido scaffolds,^[^ ^16^ ^]^ we sought to explore the viability of the ubiquitous amide anion, NH_2_^−^, as an N‐atom transfer reagent. Accordingly, heating an equimolar mixture of NaNH_2_, 18‐C‐6, and **1** in benzene‐*d_6_* for 8 h afforded the parent imido complex, [(PN)_2_Zr = NH] (**11**) in 35% yield along with the elimination of H_2_ (Scheme 1).^[^ ^68^ ^]^ Following H_2_ extrusion, the formation of **11** most likely proceeds via an imide‐hydride [(PN)_2_Zr═NH(H)]^−^, followed by loss of H^−^ presumably in the form of insoluble “NaH”. The formation of **11** also tantalizingly suggests a likely mechanism to the pnictide salts whereby the alkali hydride acts as a base to form the pnictide anion. However, the parent imide in **11** is such a weak acid (43 > p*K*_a_ > 36),^[^ ^11^ ^]^ that the deprotonation step does not take place. Formation of **11** avoids the more treacherous and lower yield synthetic protocol involving reduction of *trans*‐(PN)_2_Zr(N_3_)_2_.^[^ ^11^ ^]^ Since complex **11** is in fact a convenient precursor to the nitride complex {(PN)_2_Zr≡N[µ_2_‐Li(THF)]}_2_, this strategy provides a more straightforward alternative to traditional methods for the synthesis of parent imides as well as nitride salts.

**Figure 3:**
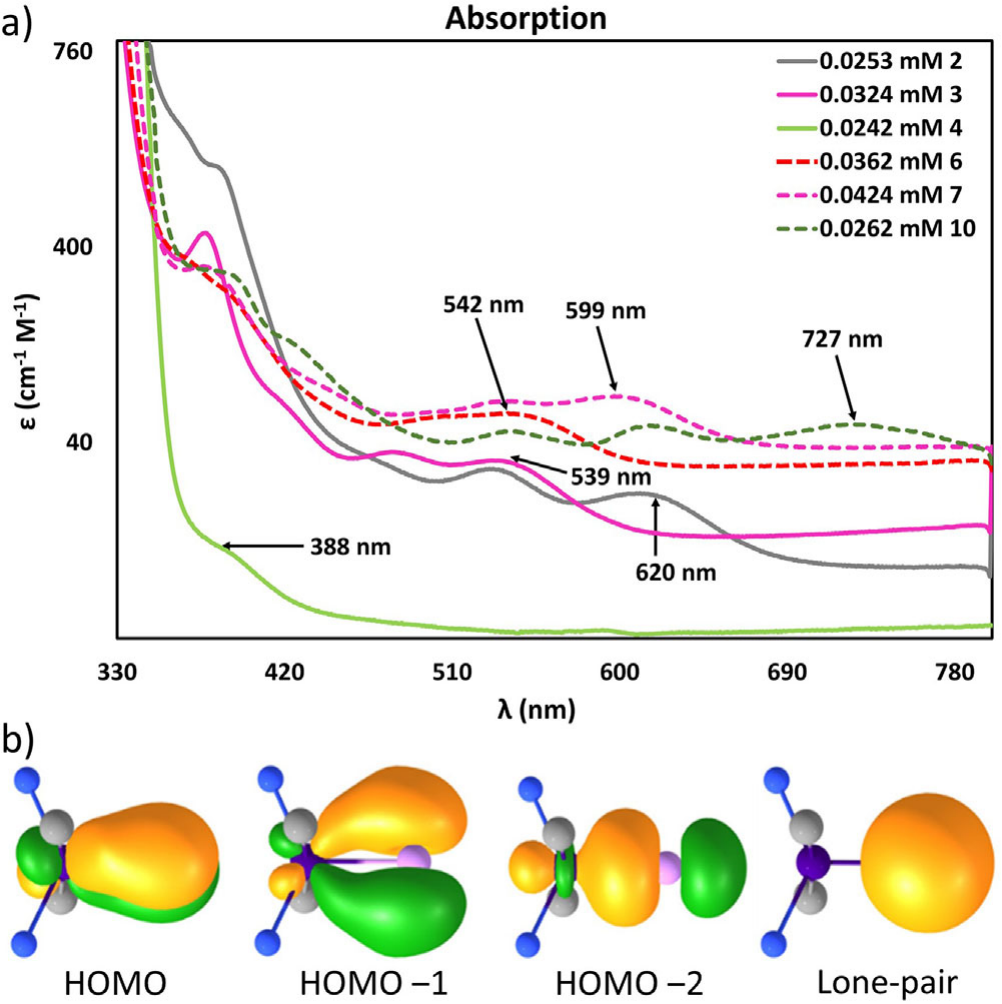
a) UV–vis spectra of Pnictide complexes (Solid‐Grey, **2**; Solid‐Pink, **3**; Solid‐Green, **4**; Dashed‐Red, **6**; Dashed‐Pink, **7**; Dashed‐Green, **10**) in THF, respectively. b) Localized molecular orbitals (LMOs) for the Zr─As bond. Contours are drawn at 0.05 a.u.

To probe the electronic features in the pnictide series we collected UV–vis spectra of the Zr^IV^ complexes **2**, **3**, and **4**. The electronic absorption spectra clearly reveal a red shift when moving down the pnictide series (388 (**4**), 539 (**3**), and 620 nm (**2**), Figure 3a). The observed differences in the UV–vis spectrum could be attributed to relativistic effects (vide infra). Notably, while the [Zr≡P]^−^ complex lacks visible region absorption bands, the [Zr≡As]^−^, and [Zr≡Sb]^−^ analogs exhibit weak but detectable transitions in the visible range. Likewise, Ti^IV^ pnictides also exhibits red shift when moving down the pnictide series (542 (**6**), 599 (**7**), and 727 nm (**10**)). The trend is consistent with a smaller HOMO‐LUMO gap due to lesser extend of orbital overlap, but also the increasing contribution of spin‐orbit coupling.^[^ ^55^ ^]^

**Figure 4:**
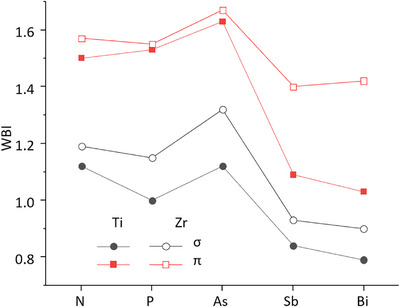
Wiberg bond indices for the Pn─M bond, broken down into σ and π contributions as one moves down the pnictogen group.

Quantum‐chemical calculations^[^ ^71^ ^]^ were conducted to a) investigate subtle details of the Pn─M bond for the different choices of Pn (Pn = N, P, As, Sb, and hypothetical Bi) and M (Ti, Zr), b) rationalize the trends in the ^31^P NMR shifts and c) clarify the nature of the lowest energy transitions in the UV–vis spectrum. For a), the ten structures were optimized at level PBE^[^ ^72^ ^]^/dhf‐TZVP^[^ ^73^ ^]^/D3‐BJ^[^ ^74^ ^]^/COSMO^[^ ^75^ ^]^ assuming *C_2_* symmetry, in reasonable agreement with scXRD, see Table 1. Next, a Pipek–Mezey localization procedure^[^ ^76^ ^]^ was applied and Wiberg bond indicies (WBIs)^[^ ^77^ ^]^ were calculated. This was done separately for the two irreducible representations, which by construction allows for fully separating σ and π contributions in the Pn─M multiple bond. Exemplary results are shown for As–Zr in Figure 3b. The localized molecular orbitals (LMOs) of all compounds are quite similar: A lone pair (LMO1), a σ‐bond (LMO2) and two π‐bonds (LMO3 and LMO4). In all cases there is Pn–M triple bond character, but the bond strength in terms of the WBIs (Figure 4) suggests two types. For N, P and As, the total WBIs amount to 2.5 to 3, thus in fact in the typical range for triple bonds whereas for Sb and Bi they are significantly lower at 1.9 to 2.3, respectively. The sudden decrease from As to Sb holds independent from the choice of M and for both the σ and π contributions; also reflected by a decrease of the electron density at the bond‐critical points from As to Sb (Table S5).

Regarding b), ^31^P NMR shielding constants were calculated at one‐ and two‐component all‐electron relativistic level within the (one‐electron) exact two‐component decoupling method, 1c‐X2C^78^ and 2c‐X2C,^[^ ^78^ ^]^ employing x2c‐TZVPall‐2c basis sets and keeping the remaining settings.^[^ ^79^ ^]^ Table 1 illustrates how the experimental chemical shifts, in particular the increase from N to Sb, are accurately reproduced by the two‐component variant^[^ ^79^ ^]^ which accounts for spin‐orbit coupling (SOC), but not by the one‐component variant^[^ ^80^ ^]^ which neglects it. This clearly indicates the role of SOC at the Pn atom on the chemical shift of the P atom (SO at heavy atom influences shift at light atom, SO‐HALA^[^ ^81^ ^]^), in this case remarkably beyond the M atom.

For c) time‐dependent DFT calculations with the PBE0^[^ ^82^ ^]^ functional were performed without considering SOC. The experimental trend of increasing wavelength for the onset of absorption when moving down the Pn series is well reproduced by the calculations, albeit significantly red‐shifted. The lowest singlet excitation (mostly HOMO and LUMO) is at 2.0/1.8/1.6 eV for Pn = P/As/Sb, M = Zr (experiment 3.2/2.3/2.0 eV), followed by excitations from HOMO‐1 to LUMO at 2.1/1.9/1.7 eV and from HOMO‐2 to LUMO at 2.5/2.3/2.0 eV. HOMO and HOMO‐1 are the Pn‐Zr π‐binding orbitals, HOMO‐2 is the σ‐binding orbital, and the LUMO is the delta d_xy_ orbital at Zr, i.e., perpendicular to the Pn─Zr bond (Figures S60 and S61). This trend also holds for the HOMO‐LUMO gap (3.3/3.0/2.8 eV for Pn = P/As/Sb, M = Zr), also when including SOC (3.3/3.0/2.7 eV at 2c‐X2C level), but then singlet‐triplet excitations are allowed, starting at 1.8/1.5/1.1 eV, and with much lower oscillator strengths, see also Table S4.

We reported here a unified and generalizable strategy for the synthesis of a series of one coordinate pnictide salt (Pn^3−^ = P, As, and Sb) complexes of Zr and Ti. Using practicable alkali metal reagents such as PnH_2_^−^, we establish H_2_ extrusion as a congruent strategy for generating discrete, terminal pnictide complexes containing M─Pn multiple bonds including unprecedented 4d and 3d metal stibides. Crystallographic,^[^ ^83^ ^]^ spectroscopic, and computational analysis reveal systematic trends in bond metrics and electronic structure, bridging known gaps between lighter and heavier congeners. We also demonstrate NaNH_2_ to serve as an NH group transfer source, offering a simplified approach to a parent Zr imide and nitride complexes but also providing clues along the H_2_ extrusion pathway.

## Conflict of Interests

The authors declare no conflict of interest.

## Supporting information



Supporting Information

Supporting Information

## Data Availability

The data that support the findings of this study are available in the Supporting Information of this article.
