# Supplementary material for: A Consistent Approach to One Coordinate Pnictide Moieties M≡Pn− Using PnH2 − Salts (M = Zr, Ti; Pn = P, As, Sb)
Source: Angew Chem Int Ed Engl. 2026 Jan 1;65(6):e23745. doi: 10.1002/anie.202523745 (PMC12865143; doi:10.1002/anie.202523745)
Supplement: Supplementary file 1 — Supporting Information [file ANIE-65-e23745-s001.pdf]

Supporting Information

for

A Consistent Approach to One Coordinate Pnictide Moieties $M{\equiv}Pn^{-}$ Using PnH_2^{-} Salts (M = Zr, Ti; Pn = P, As, Sb)

Matthew R. Mena,[‡,†] Mrinal Bhunia,[‡,†] Rishi T. Bhandari,[†] Kevin Dollberg,[€] Nick Michel,[€] Alexandra M. Bacon,[†] Michael R. Gau,[†] Florian Weigend,[§,*] Carsten von Hänisch,[€,*] and Daniel J. Mindiola[†,*]

[‡]Department of Chemistry, University of Pennsylvania, Philadelphia, PA 19104, USA

[€]Fachbereich Chemie and Marburg Center for Quantum Materials and Sustainable Technology, Philipps-Universität Marburg, Hans-Meerwein-Straße 4, 35043 Marburg, Germany

[§]Institute of Quantum Materials and Technology, Karlsruhe Institute of Technology, Hermann-von-Helmholtz-Platz 1, 76344 Karlsruhe, Germany

Corresponding authors email:

mindiola@sas.upenn.edu

carsten.vonhaenisch@staff.uni-marburg.de

florian.weigend@staff.uni-marburg.de

**1.**	**Contents**	**S2–S3**
**2.**	**Materials and Methods**	**S4–S6**
	2.1 Chemical hazards and Disposal	**S6**
**3.**	**Synthesis of Complexes**	**S7**
	3.1 [K(18-C-6)(THF)$_2$][(PN)$_2$Zr≡Sb] (**2**)	**S7–S9**
	3.2 [K(18-C-6)(THF)$_2$][(PN)$_2$Zr≡As] (**3**)	**S9–S11**
	3.3 The Heavier Pnictide Complexes of Titanium	**S11**
	3.3.1 [K(crypt)][(PN)$_2$Ti≡P] (**6**)	**S11**
	3.3.2 [K(crypt)][(PN)$_2$Ti≡As] (**7**)	**S11-S12**
	3.3.3 Reaction of **5** with K(18-C-6)(THF)$_2$SbH$_2$	**S12**
	3.4 [K(18-C-6)][(PN)$_2$TiCl] (**8**)	**S12–S14**
	3.5 [K(18-C-6)][(PN)$_2$Ti≡Sb] (**9**)	**S14–S15**
	3.6 [K(crypt)][(PN)$_2$Ti≡Sb] (**10**)	**S16–S18**
	3.7 Reaction of [(PN)(PN')Zr(H)] (**1**) with NaNH$_2$ and 18-C-6	**S18–S19**
**4.**	**NMR Spectroscopy**	**S20**
	4.1 [K(18-C-6)(THF)$_2$][(PN)$_2$Zr≡Sb] (**2**)	**S20–S30**
	4.2 [K(18-C-6)(THF)$_2$][(PN)$_2$Zr≡As] (**3**)	**S31–S41**
	4.3 ^{31}P{^{1}H} NMR spectroscopy of [K(crypt)][(PN)$_2$Ti≡P] (**6**)	**S42–S43**
	4.4 NMR spectroscopy of [K(crypt)][(PN)$_2$Ti≡As] (**7**)	**S44–S45**
	4.5 Reaction mixture NMR spectra of **5** with K(18-C-6)(THF)SbH$_2$	**S46**
	4.6 ^{1}H{^{31}P} NMR spectroscopy of [K(18-C-6)][(PN)$_2$TiCl] (**8**)	**S47**
	4.7 [K(18-C-6)][(PN)$_2$Ti≡Sb] (**9**)	**S48–S56**
	4.8 [K(crypt)][(PN)$_2$Ti≡Sb] (**10**)	**S57–S63**
	4.9 ^{1}H{^{31}P} and ^{31}P{^{1}H} NMR spectra for the reaction of [(PN)(PN')Zr(H)] (**1**) with NaNH$_2$ and 18-C-6	**S64–S66**
**5.**	**UV-*vis* Spectroscopy**	**S67**
	5.1 UV-*vis* spectroscopy of [K(18-C-6)(THF)$_2$][(PN)$_2$Zr≡Sb] (**2**)	**S67**
	5.2 UV-*vis* spectroscopy of [K(18-C-6)(THF)$_2$][(PN)$_2$Zr≡As] (**3**)	**S68**
	5.3Comparative UV-*vis* spectroscopy of **4**, **3** and **2**	**S69–S70**

5.5 UV-*vis* Spectral Data for [K(18-C-6)][$(PN)_2$Ti-Cl] (**8**) **S71–S72**
5.7 Comparative UV-*vis* spectroscopy of different Titanium pnictides **6-7**, and **10** in THF **S73**
6. **X-Ray Crystallographic Information** **S74–S83**
7. **Computational Studies** **S84**
7.1 Methodology **S84**
7.2 Excitation Energies of Ti and Zr **S85**
7.3 Data of bond-critical points for the Pn-M bond **S86**
7.4 Computational data for Zr **S87**
7.5 Computational data for Ti **S88**
7.6 Images of calculated localized orbitals of PnZr compounds **S89**
7.7 Images of calculated localized orbitals of PnTi compounds **S90**
7.8 Coordinates of optimized molecular structure for ZrPn series **S91-S102**
7.9 Coordinates of optimized molecular structure for TiPn series **S102-S113**
8. **References** **S113-S115**

2. Materials and Methods

Unless otherwise stated, all the operations were performed in a M. Braun Lab Master double-dry box under an inert atmosphere (purified dinitrogen or argon) or using high vacuum standard Schlenk techniques under dinitrogen atmosphere. Hexanes (Fisher Scientific), pentane (Fisher Scientific), diethyl ether (Et_2O, Alfa Aesar), tetrahydrofuran (THF, Fisher Scientific), and toluene (Fisher Scientific) were purchased from commercial vendors. All solvents were thoroughly bubbled with argon for 20 minutes and made anhydrous by passage through columns of activated alumina in a Grubbs-type solvent system (JC Meyer). The anhydrous solvents were stored over sodium metal and 4 Å molecular sieves (Acros Organics). Deuterated benzene (benzene−d_6) was purchased from Cambridge Isotope Laboratories (CIL) and were sparged with nitrogen for 10 minutes, then dried over a potassium mirror for overnight, vacuum transferred to a collection flask and degassed by freeze–pump–thaw cycles. Celite, charcoal, and 4 Å molecular sieves were activated under vacuum overnight at 200 °C.

The PN {PN^- = (N-(2-P^iPr_2-4-methylphenyl)-2,4,6-$Me_3C_6H_2$} ligand,[1] the discrete Ti^{II}-chloro complex [K(Krypt)][$(PN)_2TiCl$][2] (**5**), the cyclometalated Zr-hydride, [(PN)(PN')Zr(H)][3] (**1**), $NaPH_2$,[4–5] [K(18-C-6)(THF)][AsH_2],[6-7] and [K(18-C-6)(THF)][SbH_2],[8] were prepared according to published literature procedures. KC_8 was prepared by heating one equivalent of potassium metal with one equivalent of graphite at 150 °C for 1 hour in a sealed reaction vessel under a N_2 atmosphere until all metallic potassium was reacted (*caution: KC_8 is a strong alkali metal reductant and is highly pyrophoric. Extreme care must be taken when preparing and handling this reagent to avoid laboratory fires caused by exposure to air and water. Excess KC_8 can be safely quenched using a tetrahydrofuran solution of benzoic acid under an inert atmosphere*). The encapsulating agents such as 1,4,7,10,13,16-Hexaoxacyclooctadecane (18-crown-6 (18-C-6), Sigma Aldrich, 99%), and 4,7,13,16,21,24-Hexaoxa-1,10-diazabicyclo[8.8.8]-hexacosane (cryptand, Kryptofix or crypt, Sigma Aldrich, 98%) were purchased from commercial vendors and purified by heating *in vacuo* at 60 °C for overnight, and followed by recrystallization from a concentrated solution of OEt_2 and drying *in vacuo* and used. $NaNH_2$ was purchased from Sigma Aldrich and purified by heating *in vacuo* at 60 °C overnight, followed by washing with copious OEt2 and drying *in vacuo.*

NMR spectroscopic studies were carried out on a Bruker AVII 500, and UNI 500 MHz spectrometers equipped with J. Young NMR tubes. ^{1}H, ^{1}H{^{31}P}, ^{13}C{^{1}H}, ^{31}P{^{1}H}, ^{31}P, ^{1}H-^{1}H COSY, ^{1}H-^{13}C HSQC, and ^{1}H-^{13}C HMBC NMR spectral data were recorded on a Bruker AVII 500 or UNI 500 MHz spectrometer. ^{1}H NMR spectral data are reported with reference to the residual *protio* solvent resonances of benzene–d_6 at δ 7.16 and THF–d_8 at δ 3.58 and 1.73. ^{13}C{^{1}H} NMR spectra were referenced to the corresponding solvent resonances of benzene–d_6 at δ 128.06 and THF–d_8 at δ 67.57, 25.37. ^{31}P{^{1}H} NMR spectra were reported internally using MRESNOVA.[9] J values are given in Hz (s = singlet, d = doublet, t = triplet, quint = quintet, dd = doublet of doublet, sept = septet, ddd = doublet of doublet of doublet, m = multiplet).

Solution state magnetic moments were measured by Evans' method[10] using a J. Young NMR tube containing the paramagnetic complex in benzene–d_6 and a capillary containing pure benzene–d_6.

$$\chi_M = 3\ \Delta\delta\ (4\pi\ c)^{-1} \qquad\qquad \mu_{eff} = (8\ \chi_M\ T)^{½}$$

χ_M is the molar susceptibility, $\Delta\delta$ is the change in chemical shift between solvent in the capillary and the NMR sample (ppm converted to 10^{-6}), and c is the concentration (cgs units). μ_{eff} is the effective magnetic moment, and T is the temperature. Corrections for diamagnetism were made using tabulated Pascal constants.[11]

UV−vis spectroscopic studies were carried out using a Cary 5000 Spectrometer equipped with 1 cm quartz cuvette sealed with J. Young valves.

Crystallographic studies were carried out on single crystals, which were coated with paratone oil, mounted at the end of a cryoloop, and placed in the nitrogen cold stream of the diffractometer. X-ray intensity data were collected on a Rigaku XtaLAB Synergy-S diffractometer[12-13] equipped with an HPC area detector (HyPix-6000HE) and employing confocal multilayer optic-monochromated Cu-Kα radiation (λ=1.54184 Å) and a Rigaku XtaLAB Synergy-i diffractometer[14] equipped with an HPC area detector (HyPix 3000HE) and employing confocal multilayer optic-monochromated Mo-Kα radiation (λ=0.71073 Å) at a temperature of 100K. Rotation frames were integrated using CrysAlisPro,[15−16] producing a listing of unaveraged F^2 and $\sigma(F^2)$ values. The intensity data were corrected for Lorentz and polarization effects and for absorption using SCALE3 ABSPACK[17] and the structures were solved using dual space methods – SHELXT[18] and refined using SHELXL-2018 (least squares).[19] All reflections were used during refinement. The region of disordered on

solvent for which a reliable disorder model could not be devised; the X-ray data were corrected for the presence of disordered solvent using the SQUEEZE algorithm.[20] Typically these data processing was carried out in Olex2.[21]

2.1 Chemical hazards and Disposal

$NaPH_2$: Unreacted $NaPH_2$ needs to be quenched very carefully before discharge, as it is a very pyrophoric reagent. However, $NaPH_2$ was stored at -30 °C to prevent decomposition and excess $NaPH_2$ was quenched, when 2-propanol was added slowly to the dilute solution of sodium phosphanide in THF at 0 °C under argon atmosphere. Then all the glassware was rinsed with sodium hypochlorite solution to oxidize further remaining phosphanides. *Note: To avoid formation of dangerous phosphine gas, it is recommended to quench this reagent under argon atmosphere.*

K(18-C-6)(THF)AsH_2: As containing materials should be handled with care and arsenic waste should be used to collected and disposed of properly. We consolidated all our As waste and contacted the University's EHRS to collect and dispose of. Please review safety datasheets and use proper precautions.

K(18-C-6)(THF)SbH_2: Sb containing materials should be handled with care and arsenic waste should be used to collected and disposed of properly. We consolidated all our Sb waste and contacted the University's EHRS to collect and dispose of. Please review safety datasheets and use proper precautions.

3. Synthesis of Complexes

3.1 [K(18-C-6)(THF)$_2$][(PN)$_2$Zr≡Sb] (**2**)

The cyclometalated Zr-hydride, [(PN)(PN')Zr(H)] (**1**) (15.4 mg, 0.0199 mmol, 1.0 equiv.) was dissolved in 2 mL toluene in a 20 mL vial inside the N_2 filled Glove box. Then K(18-C-6)(THF)SbH_2 (11.4 mg, 0.0199 mmol, 1.0 equiv.) was dissolved in 2.5 mL toluene:THF (4:1) into a separate vial and the resultant clear light brown color solution was immediately transferred to the yellow color toluene solution of **1** at room temperature. Immediate color change from yellow to greyish-brown was observed along with evolution of molecular hydrogen. Then the reaction was further prolonged for another 5 minutes to ensure complete conversion of **1**. After completion of the reaction, it was dried *in vacuo*, affording a sticky brown residue. The residue was washed with pentane (2 x 3 mL) followed by extraction of the brown powder from 5 mL THF:toluene (2:1) followed by filtration over Celite, furnished a clear brown color solution. The resulting solution was concentrated to *ca.* 2 mL and stored the reaction mixture at -35 ˚C for 3d, afforded deposition of plate shaped greyish-brown color crystals on the walls as well as beneath of the vial which suitable for a scXRD. These crystals were isolated by decanting the mother liquor followed by washing with cold pentane and dried *in vacuo*. Further, the mother liquor was concentrated to 1 mL and stored at -35 ˚C for 2d, resulting in the deposition of second crop of greyish-brown color crystals of the discrete salt containing terminal stibido, [K(18-C-6)(THF)$_2$][(PN)$_2$Zr≡Sb] (**2**), which were further characterized through multi-nuclear NMR spectroscopy and UV-*vis* spectroscopy. Overall Yield: (25.7 mg, 0.01915 mmol, 96% yield).

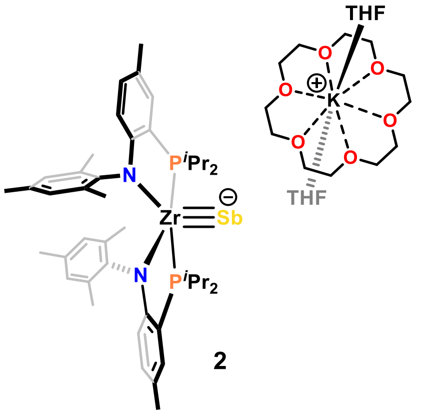

2

*Note: The discrete salt of the terminal zirconium stibide, [K(18-C-6)(THF)$_2$][(PN)$_2$Zr≡Sb] (**2**) is very stable and could be stored at -35 ˚C for several weeks. Noteworthy, **2** is highly stable in comparison to lighter congeners of zirconium pnictides such as {(PN)$_2$Zr≡N[μ_2-Li(THF)]}$_2$[22] and [Na(18-C-6)(THF)$_2$][(PN)$_2$Zr≡P][3] and [Na(222Krypt)][(PN)$_2$Zr≡P][3].*

^{1}**H NMR** (500 MHz, benzene–d_6, 298 K) of **2**: δ 7.27 (dd, J = 6.0, 3.0 Hz, 2H, C–*H*, *m*–aryl, Ar), 7.07 (d, J = 2.0 Hz, 2H, C–*H*, *m*–aryl, Mes), 6.96 (dd, J = 8.5, 2.0 Hz, 2H, C–*H*, *m*–aryl, Ar), 6.80 (d, J = 2.0 Hz, 2H, C–*H*, *m*–aryl, Mes), 6.24 (ddd, J = 8.5, 4.5, 2.0 Hz, 2H, C–*H*, *o*–aryl, Ar), 3.64 (s, $\Delta\nu_{1/2}$ = 3.0 Hz, 6H, CH_3, *o*–CH_3–aryl, Mes), 3.57 (t, J = 6.5 Hz, 8H, CH_2, O–CH_2–CH$_2$, THF), 3.11 (s, $\Delta\nu_{1/2}$ = 6.3 Hz, 24H, CH_2, O–CH_2–CH_2–O, 18-C-6), 2.71 (sept, J = 7.5 Hz, 2H, C–*H*, P–C*H*Me$_2$), 2.27 (s, $\Delta\nu_{1/2}$ = 2.8 Hz, 6H, CH_3, *p*–CH_3–aryl, Ar), 2.26 (s, $\Delta\nu_{1/2}$ = 2.8 Hz, 6H, CH_3, *p*–CH_3–aryl, Mes), 1.98 (s, $\Delta\nu_{1/2}$ = 3.0 Hz, 6H, CH_3, *o*–CH_3–aryl, Mes), 1.78 (dd, J = 13.5, 6.5 Hz, 6H, CH_3, P–CHMe_2), 1.59 (dd, J = 17.0, 10.0 Hz, 6H, CH_3, P–CHMe_2), 1.42 (quint, J = 3.0 Hz, 8H, CH_2, O–CH$_2$–CH_2, THF), 1.39 (sept, J = 6.5 Hz, 2H, C–*H*, P–C*H*Me$_2$), 1.15 (dd, J = 9.5, 3.0 Hz, 6H, CH_3, P–CHMe_2), 0.68 (dd, J = 8.0, 3.0 Hz, 6H, CH_3, P–CHMe_2). ^{1}**H{**^{31}**P} NMR** (500 MHz, benzene–d_6, 298 K): δ 7.27 (d, J = 2.5 Hz, 2H, C–*H*, *m*–aryl, Ar), 7.07 (d, J = 2.5 Hz, 2H, C–*H*, *m*–aryl, Mes), 6.95 (dd, J = 8.5, 2.5 Hz, 2H, C–*H*, *m*–aryl, Ar), 6.80 (d, J = 2.0 Hz, 2H, C–*H*, *m*–aryl, Mes), 6.23 (d, J = 8.5 Hz, 2H, C–*H*, *o*–aryl, Ar), 3.64 (s, $\Delta\nu_{1/2}$ = 3.5 Hz, 6H, CH_3, *o*–CH_3–aryl, Mes), 3.57 (t, J = 6.5 Hz, 8H, CH_2, O–CH_2–CH$_2$, THF), 3.11 (s, $\Delta\nu_{1/2}$ = 6.5 Hz, 24H, CH_2, O–CH_2–CH_2–O, 18-C-6), 2.71 (sept, J = 7.0 Hz, 2H, C–*H*, P–C*H*Me$_2$), 2.27 (s, $\Delta\nu_{1/2}$ = 3.6 Hz, 6H, CH_3, *p*–CH_3–aryl, Ar), 2.26 (s, $\Delta\nu_{1/2}$ = 3.6 Hz, 6H, CH_3, *p*–CH_3–aryl, Mes), 1.98 (s, $\Delta\nu_{1/2}$ = 3.8 Hz, 6H, CH_3, *o*–CH_3–aryl, Mes), 1.78 (d, J = 7.0 Hz, 6H, CH_3, P–CHMe_2), 1.59 (d, J = 7.0 Hz, 6H, CH_3, P–CHMe_2), 1.41 (quint, J = 6.5 Hz, 8H, CH_2, O–CH$_2$–CH_2, THF), 1.40 (sept, J = 7.5 Hz, 2H, C–*H*, P–C*H*Me$_2$), 1.15 (d, J = 6.5 Hz, 6H, CH_3, P–CHMe_2), 0.68 (d, J = 8.3 Hz, 6H, CH_3, P–CHMe_2). ^{13}**C{**^{1}**H} NMR** (125.8 MHz, benzene–d_6, 298 K): δ 160.0 (t, J = 11.3 Hz, 2C, *C*–aryl, Ar), 144.6 (t, J = 1.8 Hz, 2C, *C*–aryl, Mes), 139.4 (s, $\Delta\nu_{1/2}$ = 2.1 Hz, 2C, *o–C*–aryl, Mes), 138.8 (s, $\Delta\nu_{1/2}$ = 1.9 Hz, 2C, *o–C*–aryl, Mes), 133.6 (s, $\Delta\nu_{1/2}$ = 4.3 Hz, 2C, *C*–H, *m–C*–aryl, Ar), 132.7 (s, $\Delta\nu_{1/2}$ = 3.4 Hz, 2C, *p–C*–aryl, Mes), 132.6 (s, $\Delta\nu_{1/2}$ = 3.8 Hz, 2C, *C*–H, *m–C*–aryl, Ar), 130.5 (s, $\Delta\nu_{1/2}$ = 3.8 Hz, 2C, *C*–H, *m–C*–aryl, Mes), 129.6 (s, $\Delta\nu_{1/2}$ = 2.7 Hz, 2C, *C*–H, *m–C*–aryl, Mes), 122.3 (t, J = 1.7 Hz, 2C, *p–C*–aryl, Ar), 112.9 (t, J = 4.0 Hz, 2C, *C*–H, *o–C*–aryl, Ar), 111.8 (dd, J = 10.6, 1.5 Hz, 2C, *C*–P, *o–C*–aryl, Ar), 70.1 (s, $\Delta\nu_{1/2}$ = 3.8 Hz, 12C, *C*H$_2$, O–*C*H$_2$–*C*H$_2$–O, 18-C-6), 67.8 (s, $\Delta\nu_{1/2}$ = 1.6 Hz, 4C, *C*H$_2$, O–*C*H$_2$–CH$_2$, THF), 25.9 (s, $\Delta\nu_{1/2}$ = 2.0 Hz, 2C, *C*H$_3$, *o*–*C*H$_3$–aryl, Mes), 25.8 (s, $\Delta\nu_{1/2}$ = 1.8 Hz, 4C, *C*H$_2$, O–CH$_2$–*C*H$_2$, THF), 23.6 (s, $\Delta\nu_{1/2}$ = 3.1 Hz, 2C, *C*H$_3$, P–CHMe_2), 21.9 (t, J = 3.7 Hz, 2C, *C*H$_3$, P–CHMe_2), 21.3 (t, J = 3.5 Hz, 2C, *C*–H,

P–*C*HMe$_2$), 21.1 (s, $\Delta\nu_{1/2}$ = 1.9 Hz, 2C, *C*H$_3$, *p*–*C*H$_3$–aryl, Ar), 20.9 (s, $\Delta\nu_{1/2}$ = 2.4 Hz, 2C, *C*H$_3$, *p*–*C*H$_3$–aryl, Mes), 20.7 (s, $\Delta\nu_{1/2}$ = 2.4 Hz, 2C, *C*H$_3$, *o*–*C*H$_3$–aryl, Mes), 19.7 (t, *J* = 5.9 Hz, 2C, *C*H$_3$, P–CH*Me*$_2$), 17.4 (t, *J* = 10.4 Hz, 2C, *C*–H, P–*C*HMe$_2$), 16.8 (t, *J* = 3.1 Hz, 2C, *C*H$_3$, P–CH*Me*$_2$). **^{31}P{^{1}H} NMR** (202.5 MHz, benzene–*d*$_6$, 298 K): δ 46.47 (s, $\Delta\nu_{1/2}$ = 7.6 Hz, 2P, *P*(^{i}Pr)$_2$). **^{31}P NMR** (202.5 MHz, benzene–*d*$_6$, 298 K): δ 46.45 (s, $\Delta\nu_{1/2}$ = 53.2 Hz, 2P, *P*(^{i}Pr)$_2$). **UV-*vis***, toluene, λ [nm, ε (max/sh, M^{-1}cm^{-1})]: 307 (max, 1605), 362 (max, 602), 387 (max, 597), 532 (sh, 98), 620 (sh, 101).

3.2 [K(18-C-6)(THF)$_2$][(PN)$_2$Zr≡As] (3)

The cyclometalated Zr-hydride, [(PN)(PN')Zr(H)] (**1**) (18.6 mg, 0.0241 mmol, 1.0 equiv.) was dissolved in 3 mL toluene in a 20 mL vial inside the N$_2$ filled Glove box. Then K(18-C-6)(THF)AsH$_2$ (11.4 mg, 0.0199 mmol, 1.0 equiv.) was taken into a separate vial and dissolved with 2.5 mL toluene:THF (4:1) mixture. The resultant clear solution was transferred at room temperature to the yellow color toluene solution of **1**. Immediate color change was observed from yellow to magenta pink with evolution of molecular hydrogen. Then the reaction mixture was further prolonged at room temperature for another 5 minutes to ensure complete conversion of **1**. Next, the pink color reaction mixture was dried *in vacuo*, followed by washing with pentane (2 x 3 mL). The resultant pink color powder was extracted with 7.5 mL THF:toluene (1:2) followed by filtration over Celite, furnished a clear pink color solution. The resulting solution was concentrated to *ca.* 2.5 mL and stored the reaction mixture at -35 ˚C for 2d, affording deposition of block as well as needle shaped pink color crystals on the walls and also on the beneath of the vial, suitable for a scXRD study. These crystals were isolated by decanting the mother liquor followed by dried *in vacuo*. Further, the mother liquor was concentrated to 1 mL and stored at -35 ˚C for another 2d, afforded in the deposition of second crop of pink color crystals of terminal zirconium arsenide anion, [K(18-C-6)(THF)$_2$][(PN)$_2$Zr≡As] (**3**), which were further characterized through multi-nuclear NMR spectroscopy and UV-*vis* spectroscopy. Overall Yield:

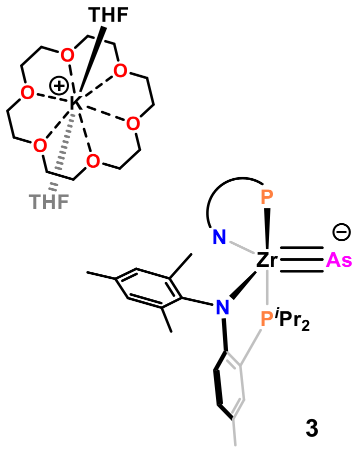

3

(28.7 mg, 0.0222 mmol, 92% yield). *Note: The discrete salt of the terminal zirconium arsenide, [K(18-C-6)(THF)$_2$][(PN)$_2$Zr≡As] (**3**) is also very stable alike the corresponding stibide complex, **2** and stable at -35 °C for several weeks.*

^{1}H NMR (500 MHz, benzene–d_6, 298 K) of **3**: δ 7.26 (dd, J = 6.0, 2.5 Hz, 2H, C–H, m–aryl, Ar), 7.10 (d, J = 2.5 Hz, 2H, C–H, m–aryl, Mes), 6.93 (dd, J = 8.5, 2.5 Hz, 2H, C–H, m–aryl, Ar), 6.85 (d, J = 2.5 Hz, 2H, C–H, m–aryl, Mes), 6.22 (ddd, J = 8.5, 5.0, 2.5 Hz, 2H, C–H, o–aryl, Ar), 3.60 (s, $\Delta\nu_{1/2}$ = 3.1 Hz, 6H, CH_3, o–CH_3–aryl, Mes), 3.06 (s, $\Delta\nu_{1/2}$ = 4.1 Hz, 24H, CH_2, O–CH_2–CH_2–O, 18-C-6), 2.79 (sept, J = 7.0 Hz, 2H, C–H, P–CHMe$_2$), 2.30 (s, $\Delta\nu_{1/2}$ = 3.7 Hz, 6H, CH_3, p–CH_3–aryl, Mes), 2.29 (s, $\Delta\nu_{1/2}$ = 3.3 Hz, 6H, CH_3, p–CH_3–aryl, Ar), 2.07 (s, $\Delta\nu_{1/2}$ = 3.2 Hz, 6H, CH_3, o–CH_3–aryl, Mes), 1.88 (dd, J = 14.0, 7.0 Hz, 6H, CH_3, P–CHMe_2), 1.61 (dd, J = 17.0, 10.0 Hz, 6H, CH_3, P–CHMe_2), 1.18 (dd, J = 9.0, 2.5 Hz, 6H, CH_3, P–CHMe_2), 0.75 (dd, J = 15.0, 8.5 Hz, 6H, CH_3, P–CHMe_2), 0.68 (sept, J = 7.0 Hz, 2H, C–H, P–CHMe$_2$). **^{1}H{^{31}P} NMR** (500 MHz, benzene–d_6, 298 K): δ 7.26 (d, J = 2.5 Hz, 2H, C–H, m–aryl, Ar), 7.10 (d, J = 2.0 Hz, 2H, C–H, m–aryl, Mes), 6.92 (dd, J = 8.5, 2.5 Hz, 2H, C–H, m–aryl, Ar), 6.85 (d, J = 2.0 Hz, 2H, C–H, m–aryl, Mes), 6.21 (d, J = 8.5 Hz, 2H, C–H, o–aryl, Ar), 3.60 (s, $\Delta\nu_{1/2}$ = 3.8 Hz, 6H, CH_3, o–CH_3–aryl, Mes), 3.07 (s, $\Delta\nu_{1/2}$ = 4.4 Hz, 24H, CH_2, O–CH_2–CH_2–O, 18-C-6), 2.79 (sept, J = 7.0 Hz, 2H, C–H, P–CHMe$_2$), 2.29 (s, $\Delta\nu_{1/2}$ = 4.3 Hz, 6H, CH_3, p–CH_3–aryl, Mes), 2.28 (s, $\Delta\nu_{1/2}$ = 5.4 Hz, 6H, CH_3, p–CH_3–aryl, Ar), 2.06 (s, $\Delta\nu_{1/2}$ = 4.5 Hz, 6H, CH_3, o–CH_3–aryl, Mes), 1.88 (d, J = 7.0 Hz, 6H, CH_3, P–CHMe_2), 1.61 (d, J = 7.0 Hz, 6H, CH_3, P–CHMe_2), 1.18 (d, J = 7.0 Hz, 6H, CH_3, P–CHMe_2), 0.75 (d, J = 6.5 Hz, 6H, CH_3, P–CHMe_2), 0.69 (sept, J = 7.0 Hz, 2H, C–H, P–CHMe$_2$). **^{13}C{^{1}H} NMR** (125.8 MHz, benzene–d_6, 298 K): δ 161.0 (t, J = 11.3 Hz, 2C, C–aryl, Ar), 145.4 (t, J = 1.8 Hz, 2C, C–aryl, Mes), 139.4 (s, $\Delta\nu_{1/2}$ = 2.1 Hz, 2C, o–C–aryl, Mes), 138.8 (s, $\Delta\nu_{1/2}$ = 2.1 Hz, 2C, o–C–aryl, Mes), 133.3 (s, $\Delta\nu_{1/2}$ = 3.8 Hz, 2C, C–H, m–C–aryl, Ar), 132.6 (s, $\Delta\nu_{1/2}$ = 3.4 Hz, 2C, p–C–aryl, Mes), 132.5 (s, $\Delta\nu_{1/2}$ = 2.1 Hz, 2C, C–H, m–C–aryl, Ar), 130.3 (s, $\Delta\nu_{1/2}$ = 2.8 Hz, 2C, C–H, m–C–aryl, Mes), 129.5 (s, $\Delta\nu_{1/2}$ = 2.8 Hz, 2C, C–H, m–C–aryl, Mes), 121.3 (t, J = 1.8 Hz, 2C, p–C–aryl, Ar), 112.6 (t, J = 4.3 Hz, 2C, C–H, o–C–aryl, Ar), 111.6 (dd, J = 10.1, 1.3 Hz, 2C, C–P, o–C–aryl, Ar), 69.9 (s, $\Delta\nu_{1/2}$ = 3.9 Hz, 12C, CH$_2$, O–CH$_2$–CH$_2$–O, 18-C-6), 23.7 (s, $\Delta\nu_{1/2}$ = 2.0 Hz, 2C, CH$_3$, o–CH$_3$–aryl, Mes), 21.9 (t, J = 8.6 Hz, 2C, C–H, P–CHMe$_2$, merge with P–CHMe_2), 21.8 (dd, J = 8.6, 7.8 Hz, 2C, CH$_3$, P–CHMe_2, merge with P–CHMe$_2$),

21.2 (s, $\Delta\nu_{1/2}$ = 2.6 Hz, 2C, *C*H_3, P–CH*Me*$_2$), 21.1 (s, $\Delta\nu_{1/2}$ = 2.1 Hz, 2C, *C*H_3, *p*–*C*H_3–aryl, Mes), 20.9 (s, $\Delta\nu_{1/2}$ = 2.0 Hz, 2C, *C*H_3, *p*–*C*H_3–aryl, Ar), 20.5 (s, $\Delta\nu_{1/2}$ = 1.6 Hz, 2C, *C*H_3, *o*–*C*H_3–aryl, Mes), 19.4 (dd, J = 10.4, 0.6 Hz, 2C, *C*–H, P–*C*HMe$_2$), 19.2 (t, J = 6.4 Hz, 2C, *C*H_3, P–CH*Me*$_2$), 16.5 (dd, J = 3.3, 0.6 Hz, 2C, *C*H_3, P–CH*Me*$_2$). **$^{31}P\{^1H\}$ NMR** (202.5 MHz, benzene–d_6, 298 K): δ 31.21 (s, $\Delta\nu_{1/2}$ = 3.4 Hz, 2P, *P*(^{i}Pr)$_2$). **^{31}P NMR** (202.5 MHz, benzene–d_6, 298 K): δ 31.18 (s, $\Delta\nu_{1/2}$ = 52.0 Hz, 2P, *P*(^{i}Pr)$_2$). **UV-*vis***, toluene, λ [nm, ε (max/sh, $M^{-1}cm^{-1}$)]: 319 (max, 1790), 378 (max, 627), 478 (sh, 102), 539 (sh, 91).

3.3 The Heavier Pnictide Complexes of Titanium

3.3.1 [K(crypt)][(PN)$_2$Ti≡P] (6)

Inside the N_2 filled Glove box, [K(crypt)][(PN)$_2$TiCl] (**5**) (10 mg, 0.008 mmol, 1 equiv.) was dissolved in THF–h_8 (0.4 mL) in an J-Young NMR tube. Then THF–h_8 (0.4 mL) solution of $NaPH_2$ (1.5 mg, 0.026 mmol, 3 equiv.) was added into the J-Young NMR tube at room temperature. The color of the reaction mixture changed from dark brown to pinkish red upon heating to 60 °C for 30 min. $^{31}P\{^1H\}$ NMR spectroscopy was recorded, which revealed the clean formation of [K(crypt)][(PN)$_2$Ti≡P] (**6**). Workup of the reaction mixture resulted in quantitative yield (10.2 mg, 0.008 mmol, 99% yield).

3.3.2 [K(crypt)][(PN)$_2$Ti≡As] (7)

Inside the N_2 filled Glove box, [K(crypt)][(PN)$_2$TiCl] (**5**) (4.5 mg, 0.0038 mmol, 1 equiv.) was dissolved in benzene–d_6 (0.4 mL) in an J-Young NMR tube. Then K(18-C-6)(THF)AsH_2 (1.9 mg, 0.0038 mmol, 1 equiv.) was dissolved in benzene–d_6 (0.2 mL) and transferred to the brown color solution of **5** into the J-Young NMR tube at room temperature. The color of the reaction mixture immidiately changed from dark brown to pink-red, and monitoring the NMR (^1H and ^{31}P) spectroscopy resulted similar complex reaction mixture as **6** with the evolution of molecular hydrogen, affording clean formation of [K(crypt)][(PN)$_2$Ti≡As] (**7**). Yield (4.3 mg, 0.0035 mmol, 92% yield).

3.3.3 Reaction of 5 with K(18-C-6)(THF)SbH_2

Inside the N_2 filled Glove box, [K(crypt)][$(PN)_2$TiCl] (**5**) (16 mg, 0.014 mmol, 1 equiv.) was dissolved in benzene–d_6 (0.5 mL) in an J-Young NMR tube. Then K(18-C-6)(THF)SbH_2 (7.5 mg, 0.014 mmol, 1 equiv.) was taken into separate vial and dissolved with benzene–d_6 (0.3 mL) and transferred to the solution of **5** into the J-Young NMR tube at room temperature. The color of the reaction mixture changed from dark brown to dark green along with evolution of molecular hydrogen. NMR (^{1}H and ^{31}P) spectroscopy study revealed formation of new diamagnetic complex. Subsequent workup and recrystalization of the THF soluble green product at -35 °C always afforded **10** marred with **5** regardless of conditions.

Thus, we sought to prepare more soluble Ti^{II}-chloro ate complex using 18-C-6 ether instead of cryptand and the procedure is described below.

3.4 [K(18-C-6)][$(PN)_2$TiCl] (8)

The titanium(III) chloro complex [$(PN)_2$TiCl] (118.8 mg, 0.155 mmol, 1 equiv.) was dissolved in 5 mL toluene in a 20 mL vial and one magnetic stirrer bar was added to it inside the N_2 filled Glove box. Then KC_8 (27.3 mg, 0.202 mmol, 1.3 equiv.) was taken into a separate vial and 2 mL toluene was added to it. In another vial, the 18-C-6 (41.0 mg, 0.155 mmol, 1 equiv.) was dissolved in 2 mL toluene and transferred it to the suspension of KC_8 in toluene. The resulting yellow suspension was immediately transferred to the dark red color toluene solution of [$(PN)_2$TiCl]. Then the reaction mixture was stirred at room temperature and immediate color change was observed from dark red to greenish-brown with concomitant graphite deposition. Stirring the reaction for 12 h at room temperature afforded a greenish solution that was filtered through a Celite plug to remove the graphite and washed with toluene (3 x 3 mL). All the volatiles were removed *in vacuo* and the remaining green solid was washed with pentane (3 x 3 mL) and dried to afford crude, analytically pure **8**. Yield: (150 mg,

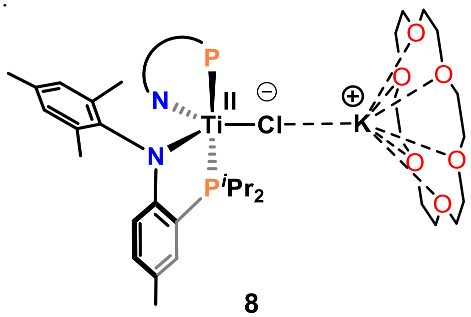

8

0.141 mmol, 90%). Crystals suitible for a scXRD study was obtained by concentrating ~20 mg of **8** in 1 mL toluene in a small 4 mL vial and placing in a 20 mL scintilation vial containing 5 mL pentane at -35 ˚C for 3 days as green blocks.

^{1}H NMR (500 MHz, benzene–d_6, 298 K): δ 15.54 (brs, $\Delta\nu_{1/2}$ = 428.5.5 Hz), 14.17 (brs, $\Delta\nu_{1/2}$ = 360.9 Hz), 5.12 (brs, $\Delta\nu_{1/2}$ = 22.4 Hz), 2.99 (brs, $\Delta\nu_{1/2}$ = 37.30 Hz, O–CH_2–CH_2–O, 18-C-6), 1.46 (brs, $\Delta\nu_{1/2}$ = 20.8 Hz), 0.29 (brs, $\Delta\nu_{1/2}$ = 6.6 Hz). **UV-*vis***, toluene λ [nm, ε (max/sh, $M^{-1}cm^{-1}$)]: 290 (max, 8664), 329 (max, 12265), 385 (max, 6029), 667 (sh, 620). **Magnetic moment** (Evans' method, benzene–d_6, 298 K): 2.61 μ_B.

Note: The same reaction when conducted in THF instead of toluene, a discrete salt formed which mentioned below-

*The titanium(III) chloro complex [(PN)$_2$TiCl] (60.0 mg, 0.0785 mmol, 1 equiv.) was dissolved in 3 mL THF in a 20 mL vial inside the N_2 filled Glove box. Then 3 mL THF was added to the mixture of KC_8 (11.2 mg, 0.08244 mmol, 1.05 equiv.) and 18-C-6 (20.8 mg, 0.0785 mmol, 1 equiv.) and the light blue color solution immediately transferred to the red color solution of [(PN)$_2$TiCl] at room temperature. Further, the reaction mixture was stirred at room temperature for overnight and dried in vacuo resulting brown color sticky residue. The residue was washed with pentane (2 x 5 mL) followed by extraction with thf (ca. 5 mL). The resulting brown color solution was concentrated under reduced pressure to ca. 2 mL and stored at -35 ˚C for 7 days, affording deposition of plate shaped dark-brown crystals which suitable for a scXRD. An scXRD study revealed the connectivity as well formation of discrete salt, [K(18-C-6)(THF)$_2$][(PN)$_2$TiCl] (**8-THF$_2$**). However, **8-THF$_2$** is not so stable, always formed sticky liquid and thus inhibits spectroscopic characterization and reliable yield.*

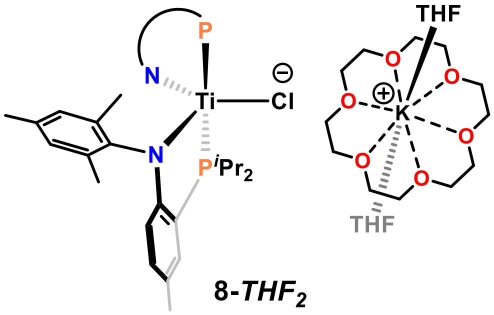

8-*THF$_2$*

3.5 [K(18-C-6)][(PN)$_2$Ti≡Sb] (9)

The contact pair of Ti^{II}-ate complex, [K(18-C-6)][(PN)$_2$TiCl] (**8**) (47.0 mg, 0.0445 mmol, 1 equiv.) was dissolved in 3 mL benzene in a 20 mL vial containing one stir bar inside the N_2 filled Glove box. Then K(18-C-6)(THF)SbH$_2$ (27.1 mg, 0.0489 mmol, 1.1 equiv.) was dissolved in benzene (3 mL) and the resulting clear solution was added dropwise to the stirring solution of **8** at room temperature. Within a few minutes, a color change was observed from greenish-brown to dark green. The reaction was allowed to stir at room temperature for 1 h. After which, the solution was filtered through Celite and the volatiles removed *in vacuo*, affording a dark green colored solid. Next, the green color solid was washed with pentane (3 x 3 mL) and extracted with toluene (*ca.* 2 mL) followed by filtration over Celite into a small 4 mL vial which was placed in a 20 mL scintilliation vial containing 10 mL pentane and stored at -35 ˚C for 3 days which afforded block shaped green colored crystals on the walls of the vial suitable for a scXRD study. These crystals were isolated by decanting the mother liquor followed by washing with cold pentane (2 x 1 mL) and drying *in vacuo*. The remaining mother liquor was stored at -35 ˚C for another 5 days, affording green colored crystals. Decantation of the mother liquor and drying the precipitate *in vacuo* resulted in the isolation of the analytically pure, close contact ion pair of the titanium stibide, [K(18-C-6)][(PN)$_2$Ti≡Sb] (**9**). Overall Yield: (46.8 mg, 0.0407 mmol, 92% yield). ^{1}**H NMR** (500 MHz, benzene–d_6, 298 K) of **9**: δ 7.47 (dd, J = 5.9, 3.0 Hz, 2H, C–H, m–aryl, Ar), 7.20 (d, J = 2.0 Hz, 2H, C–H, m–aryl, Mes), 7.00 (dd, J = 8.5, 2.0 Hz, 2H, C–H, m–aryl, Ar), 6.76 (d, J = 2.5 Hz, 2H, C–H, m–aryl, Mes), 6.26 (dd, J = 8.5, 4.0 Hz, 2H, C–H, o–aryl, Ar), 3.71 (s, $\Delta\nu_{1/2}$ = 3.2 Hz, 6H, CH_3, o–CH_3–aryl, Mes), 3.27 (s, $\Delta\nu_{1/2}$ = 8.0 Hz, 24H, CH_2, O–CH_2–CH_2–O, 18-C-6), 3.17 (sept, J = 7.0 Hz, 2H, C–H, P–CHMe$_2$), 2.34 (s, $\Delta\nu_{1/2}$ = 3.3 Hz, 6H, CH_3, p–CH_3–aryl, Ar), 2.33 (s, $\Delta\nu_{1/2}$ = 3.4 Hz, 6H, CH_3, p–CH_3–aryl, Mes), 1.91 (dd, J = 7.0, 5.5 Hz, 6H, CH_3, P–CHMe_2, merge with another P–CHMe_2), 1.88 (dd, J = 11.0, 6.5 Hz, 6H, CH_3, P–CHMe_2, merge with other P–CHMe_2), 1.58 (s, $\Delta\nu_{1/2}$ = 3.4 Hz, 6H, CH_3,

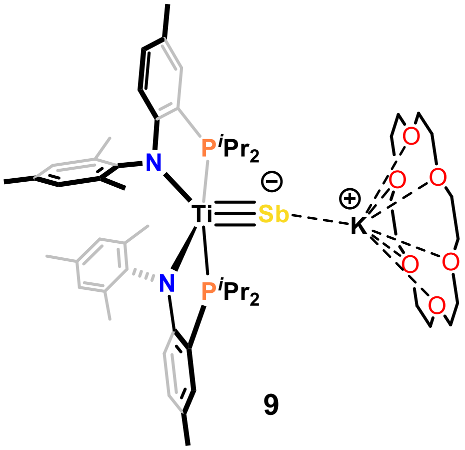

9

o–CH_3–aryl, Mes), 1.33 (t, *J* = 7.0 Hz, 6H, CH_3, P–CH*Me*$_2$), 0.68 (sept, *J* = 7.0 Hz, 2H, C–*H*, P–C*H*Me$_2$), 0.44 (dd, *J* = 8.0, 7.0 Hz, 6H, CH_3, P–CH*Me*$_2$). **^{13}C{^{1}H} NMR** (125.8 MHz, benzene–d_6, 298 K): δ 159.4 (t, *J* = 11.8 Hz, 2C, *C*–aryl, Ar), 148.8 (t, *J* = 2.1 Hz, 2C, *C*–aryl, Mes), 138.5 (s, $\Delta\nu_{1/2}$ = 2.0 Hz, 2C, *o*–*C*–aryl, Mes), 137.9 (s, $\Delta\nu_{1/2}$ = 2.2 Hz, 2C, *o*–*C*–aryl, Mes), 133.3 (s, $\Delta\nu_{1/2}$ = 4.1 Hz, 2C, *C*–H, *m*–*C*–aryl, Ar), 132.7 (s, $\Delta\nu_{1/2}$ = 3.2 Hz, 2C, *C*–H, *m*–*C*–aryl, Ar), 132.4 (s, $\Delta\nu_{1/2}$ = 2.2 Hz, 2C, *C*–Ar, *p*–*C*–aryl, Mes), 130.4 (s, $\Delta\nu_{1/2}$ = 3.0 Hz, 2C, *C*–H, *m*–*C*–aryl, Mes), 129.3 (s, $\Delta\nu_{1/2}$ = 3.3 Hz, 2C, *C*–H, *m*–*C*–aryl, Mes), 122.1 (s, $\Delta\nu_{1/2}$ = 4.2 Hz, 2C, *C*–Ar, *p*–*C*–aryl, Ar), 112.5 (t, *J* = 4.0 Hz, 2C, *C*–H, *o*–*C*–aryl, Ar), 111.0 (dd, *J* = 9.1, 3.5 Hz, 2C, *C*–Ar, *C*–P, *o*–*C*–aryl, Ar), 70.6 (s, $\Delta\nu_{1/2}$ = 3.9 Hz, 12C, *C*H$_2$, O–*C*H$_2$–*C*H$_2$–O, 18-C-6), 27.3 (s, $\Delta\nu_{1/2}$ = 2.4 Hz, 2C, *C*H$_3$, *o*–*C*H$_3$–aryl, Mes), 26.9 (t, *J* = 2.6 Hz, 2C, *C*H$_3$, P–CH*Me*$_2$), 23.6 (s, $\Delta\nu_{1/2}$ = 3.0 Hz, 2C, *C*–H, P–*C*HMe$_2$), 21.9 (t, *J* = 3.6 Hz, 2C, *C*H$_3$, P–CH*Me*$_2$), 21.2 (s, $\Delta\nu_{1/2}$ = 2.2 Hz, 2C, *C*H$_3$, *p*–*C*H$_3$–aryl, Mes), 21.1 (s, $\Delta\nu_{1/2}$ = 2.1 Hz, 2C, *C*H$_3$, *p*–*C*H$_3$–aryl, Ar), 20.5 (t, *J* = 6.4 Hz, 2C, *C*H$_3$, P–CH*Me*$_2$), 20.3 (s, $\Delta\nu_{1/2}$ = 1.8 Hz, 2C, *C*H$_3$, *o*–*C*H$_3$–aryl, Mes), 20.2 (dd, *J* = 9.1, 3.3 Hz, 2C, *C*–H, P–*C*HMe$_2$), 17.6 (t, *J* = 3.6 Hz, 2C, *C*H$_3$, P–CH*Me*$_2$). **^{31}P{^{1}H} NMR** (202.5 MHz, benzene–d_6, 298 K): δ 58.4 (s, $\Delta\nu_{1/2}$ = 8.9 Hz, 2P, *P*(^{i}Pr)$_2$).

*Note: The close contact ion pair of titanium stibide, [K(18-C-6)][(PN)$_2$Ti≡Sb] (**9**) is stabilized in dimeric form where the contact ion pairing of partially encapsulated Sb…K(18-C-6) is interacted with "O6" atom of another K(18-C-6)…Sb unit, as shown in Figure S59 (K1…O6 = 2.898(2) Å).*

3.6 [K(crypt)][(PN)$_2$Ti≡Sb] (10)

The one coordinate terminal titanium stibide i.e, discreate salt [K(crypt)][(PN)$_2$Ti≡Sb] (**10**) can be prepared either from the complex **8** in presence of K(18-C-6)(THF)SbH_2 and cryptand, or, from the reaction of complex **9** and cryptand.

3.6.1 Reaction of 8 with K(18-C-6)(THF)SbH$_2$ and cryptand.

The contact pair of Ti^{II}-ate complex, [K(18-C-6)][(PN)$_2$TiCl] (**8**) (40.0 mg, 0.037 mmol, 1 equiv.) was dissolved in 3 mL benzene in a 20 mL vial containing one stir bar inside the N_2 filled Glove box. Then K(18-C-6)(THF)SbH_2 (18 mg, 0.037 mmol, 1.0 equiv.) was dissolved in benzene (3 mL) and the resulting faint yellow suspension was added dropwise to the stirring solution of **3** at room temperature. Within a few minutes, a color change was observed from greenish-brown to dark green. The reaction was allowed to stir at room temperature for 1.5 h. Then, to the following benzene solution was added cryptand (12.4 mg, 0.037, 1.0 equiv.) in THF (3 mL) and the green solution was allowed to stir for 1.5 hr where no visible color change was observed. After, all the volatiles were removed *in vacuo*, affording a dark green colored solid. Next, the green color solid was washed with pentane (4 x 3 mL) and extracted with thf (*ca.* 10 mL) followed by filtration over Celite, maintaining the dark green colored solution. The thf solution was concentrated to *ca.* 2 mL and filtered over Celite into a small 4 mL vial which was placed in a 20 mL scintillation vial that contained pentane (10 mL) and stored at -35 ˚C for 4 days, afforded deposition of needle shaped dark green colored crystals on the walls of the vial suitable for a scXRD. It is important to note if green colored blocks are obtained, this will contain **5** in the unit cell. These needle shaped crystals were isolated by decanting the mother liquor, washed with cold pentane (3x 2 mL) followed by drying *in vacuo*. The remaining mother liquor was stored at -35 ˚C for another 2 days, affording more crystals. Decantation of the mother liquor and drying the precipitate *in vacuo* resulted in the isolation of the analytically and spectroscopically pure discrete salt of one coordinated terminal titanium stibide,

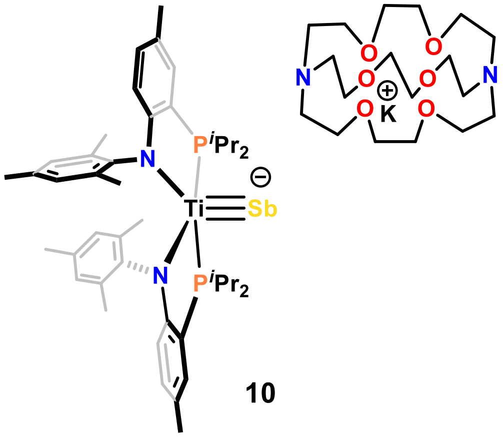

10

[K(crypt)][(PN)$_2$Ti≡Sb] (**10**). Overall Yield: (41.1 mg, 0.033 mmol, 86% yield). **^{1}H NMR** (500 MHz, THF–d_8 298 K) of **10**: δ 7.10 (dd, J = 6.0, 3.0 Hz, 2H, C–*H*, *m*–aryl, Ar), 6.89 (s, $\Delta\nu_{1/2}$ = 2.5 Hz,, 2H, C–*H*, *m*–aryl, Mes), 6.60 (dd, J = 9.0, 2.0 Hz, 2H, C–*H*, *m*–aryl, Ar), 6.48 (d, J = 2.4 Hz, 2H, C–*H*, *m*–aryl, Mes), 5.58 (dd, J = 8.5, 4.0 Hz, 2H, C–*H*, *o*–aryl, Ar), 3.55 (s, $\Delta\nu_{1/2}$ = 4.1 Hz, 12H, C*H$_2$*, O–C*H$_2$*–C*H$_2$*–O, 222Krypt), 3.50 (t, J = 4.5 Hz, 12H, C*H$_2$*, O–C*H$_2$*–CH$_2$–O, 222Krypt), 3.29 (s, $\Delta\nu_{1/2}$ = 2.6 Hz, 6H, C*H$_3$*, *o*–C*H$_3$*–aryl, Mes), 2.85 (sept, J = 7.0 Hz, 2H, C–*H*, P–C*H*Me$_2$), 2.51 (t, J = 4.5 Hz, 12H, C*H$_2$*, O–CH$_2$–C*H$_2$*–N, 222Krypt), 2.21 (s, $\Delta\nu_{1/2}$ = 2.6 Hz, 6H, C*H$_3$*, *p*–C*H$_3$*–aryl, Mes), 2.18 (s, $\Delta\nu_{1/2}$ = 2.4 Hz, 6H, C*H$_3$*, *p*–C*H$_3$*–aryl, Ar), 1.61 (dd, J = 12.0, 5.0 Hz, 6H, C*H$_3$*, P–CH*Me$_2$*, merge with another P–CH*Me$_2$* at 1.57 ppm), 1.57 (dd, J = 16.0, 9.0 Hz, 6H, C*H$_3$*, P–CH*Me$_2$*, merge with other P–CH*Me$_2$* at 1.61 ppm), 1.01 (s, $\Delta\nu_{1/2}$ = 2.8 Hz, 6H, C*H$_3$*, *o*–C*H$_3$*–aryl, Mes), 0.99 (dd, J = 4.5, 2.0 Hz, 6H, C*H$_3$*, P–CH*Me$_2$*), 0.27 (sept, J = 7.0 Hz, 2H, C–*H*, P–C*H*Me$_2$), 0.12 (dd, J = 14.0, 7.0 Hz, 6H, C*H$_3$*, P–CH*Me$_2$*). **^{13}C{^{1}H} NMR** (125.8 MHz, THF–d_8, 298 K) δ 159.3 (t, J = 12.6 Hz, , 2C, *C*–aryl, Ar), 149.7 (t, J = 2.1 Hz, 2C, *C*–aryl, Mes), 138.7 (s, $\Delta\nu_{1/2}$ = 2.7 Hz, 2C, *o*–*C*–aryl, Mes), 138.4 (s, $\Delta\nu_{1/2}$ = 2.0 Hz, 2C, *o*–*C*–aryl, Mes), 133.8 (s, $\Delta\nu_{1/2}$ = 3.7 Hz, 2C, *m*–*C*–aryl, Ar), 131.8 (s, $\Delta\nu_{1/2}$ = 3.6 Hz, 2C, *C*–H, *m*–*C*–aryl, Ar), 130.6 (s, $\Delta\nu_{1/2}$ = 2.7 Hz, 2C, *C*–Ar, *m*–*C*–aryl, Mes), 129.7 (s, $\Delta\nu_{1/2}$ = 2.7 Hz, 2C, *p*–*C*–aryl, Mes), 128.8 (s, $\Delta\nu_{1/2}$ = 3.2 Hz, 2C, *C*–H, *m*–*C*–aryl, Mes), 120.9 (t, J = 2.5 Hz, 2C, *p*–*C*–aryl, Ar), 112.1 (t, J = 3.8 Hz, 2C, *C*–H, *o*–*C*–aryl, Ar), 111.4 (dd, J = 3.8, 2.5 Hz, 2C, *C*–P, *o*–*C*–aryl, Ar), 71.5 (s, $\Delta\nu_{1/2}$ = 3.9 Hz, 6C, *C*H$_2$, O–*C*H$_2$–*C*H$_2$–O, 222Krypt), 68.7 (s, $\Delta\nu_{1/2}$ = 3.9 Hz, 6C, *C*H$_2$, O–*C*H$_2$–CH$_2$–N, 222Krypt), 55.0 (s, $\Delta\nu_{1/2}$ = 3.9 Hz, 6C, *C*H$_2$, O–CH$_2$–*C*H$_2$–N, 222Krypt), 27.4 (s, $\Delta\nu_{1/2}$ = 3.0 Hz, *C*H$_3$, *o*–*C*H$_3$–aryl, Mes), 26.4 (s, $\Delta\nu_{1/2}$ = 2.2 Hz, 2C, *C*H$_3$, P–CH*Me$_2$*), 23.9 (s, $\Delta\nu_{1/2}$ = 2.7 Hz, 2C, *C*–H, P–*C*HMe$_2$), 22.0 (t, J = 3.3 Hz, 2C, *C*H$_3$, P–CH*Me$_2$*), 21.1 (s, $\Delta\nu_{1/2}$ = 2.7 Hz, 2C, *C*H$_3$, *p*–*C*H$_3$–aryl, Mes), 21.0 (s, $\Delta\nu_{1/2}$ = 2.7 Hz, 2C, *C*H$_3$, *p*–*C*H$_3$–aryl, Ar), 20.7 (s, $\Delta\nu_{1/2}$ = 2.6 Hz, 2C, *C*–H, P–*C*HMe$_2$), 20.6 (s, $\Delta\nu_{1/2}$ = 3.2 Hz, 2C, *C*H$_3$, P–CH*Me$_2$*), 19.5 (s, $\Delta\nu_{1/2}$ = 3.6 Hz, 2C, *C*H$_3$, P–CH*Me$_2$*), 17.7 (t, J = 3.8 Hz, 2C, *C*H$_3$, *o*–*C*H$_3$–aryl, Mes). **^{31}P{^{1}H} NMR** (202.5 MHz, THF–d_8, 298 K): δ 57.0 (s, $\Delta\nu_{1/2}$ = 5.7 Hz, 2P, *P*(^{i}Pr)$_2$). **UV-*vis***, THF, λ [nm, ε (max/sh, M^{-1}cm^{-1})]: 332 (max, 971), 388 (max, 320), 420 (max, 161) 537 (sh, 92), 615 (sh, 109), 727 (sh, 73).

3.6.2 Reaction of 4 with cryptand.

Inside the N_2 filled Glove box, [K(18-C-6)][$(PN)_2$Ti≡Sb] (**9**) (10.2 mg, 0.008 mmol, 1 equiv.) was dissolved in 1 mL toluene and cryptand (2.9 mg, 0.008, 1 equiv.) in 3 mL thf was added to the green colored solution of **9** at room temperature. No discernable color change was observed immediate after addition of cryptand. After 3 hr stirring at room temperature no precipitate or color change was observed after which all volatiles were removed *in vacuo*, and the green residue was washed with pentane (3 x 3 mL). The green solid was extracted in 2 mL thf and filtered over Celite into a small 4 mL vial and placed in a 20 mL scintillation vial containing 5 mL pentane and stored at -35 °C for 4 days yielding dark green needle crystals. The mother liquor was decanted, crystals washed with cold pentane (2 x 1 mL) and dried *in vacuo*. The mother liquor can be pumped down and washed with cold pentane to yield spectroscopically pure **10** (^{1}H and ^{31}P NMR). Combined Yield: (10 mg, 0.008, ~99% yield)

3.7 Reaction of [(PN)(PN')Zr(H)] (1) with $NaNH_2$ and 18-C-6

In order to show the versatile nature of PnH_2^- (Pn = N, P, As, and Sb) for the installation of terminal pnictides having Pn^{3-} moiety, after successful synthesis of terminal zirconium pnictide salts, Zr≡Pn^{3-} (Pn = P, As, and Sb), we focused to synthesize terminal Zr≡N^{3-}. However, performing the similar reaction like other Zr≡Pn^{3-} (Pn = P, As, and Sb), reaction of [(PN)(PN')Zr(H)] (**1**) with 3 equiv. $NaNH_2$ and 3 equiv. 18-C-6, afforded parent terminal imide, $(PN)_2$Zr=NH (**11**) in 35% yield. *It should be noted that the same reaction using 1 equiv. $NaNH_2$ and 1 equiv. 18-C-6 was performed and monitoring the NMR spectroscopy (^{1}H and ^{31}P{^{1}H}) indicates no though full conversion of **1** for the isolation of parent zirconium imide, **11** (Figures S45 and S46).* The reaction procedure is described below-

Inside the N_2 filled Glove box, [(PN)(PN')Zr(H)] (**1**) (16.3 mg, 0.0203 mmol, 1 equiv.) was dissolved in benzene–d_6 (0.5 mL) in an J-Young NMR tube. Then 18-C-6 (16.1 mg, 0.0609 mmol, 3 equiv.) was dissolved in benzene–d_6 (0.2 mL) and the resultant clear solution was transferred to the another vial containing $NaNH_2$ (2.4 mg, 0.0609 mmol, 3 equiv.) at room temperature. The resulting clear suspension was added to the J-Young NMR tube containing **1** in benzene–d_6 at room temperature. The color of the reaction mixture did not change upon immediate addition.

However, the color of the reaction mixture changed to yellow upon heating at 60 °C for overnight. Monitoring the $^{31}P\{^{1}H\}$ NMR spectoscopy, clearly indicates the formation of terminal zirconium imides, **11** along with minor PN-H and PN-Na and also, minute amount of unidentified byproducts. The reaction mixture was brought inside N_2 filled glovebox, and the volatiles were removed *in vacuo*. The yellow residue was extracted in pentane (3 mL) and filtered through Celite into a small 4 mL vial and placed into a 20 mL scintillation vial containing toluene (2 mL) as sorbent to yield yellow crystals after 4 days at -35 °C. Yield (5.8 mg, 0.0072 mmol, 35%).

4. NMR Spectroscopy

4.1 NMR Spectral Data for [K(18-C-6)(THF)$_2$][(PN)$_2$Zr≡Sb] (2)

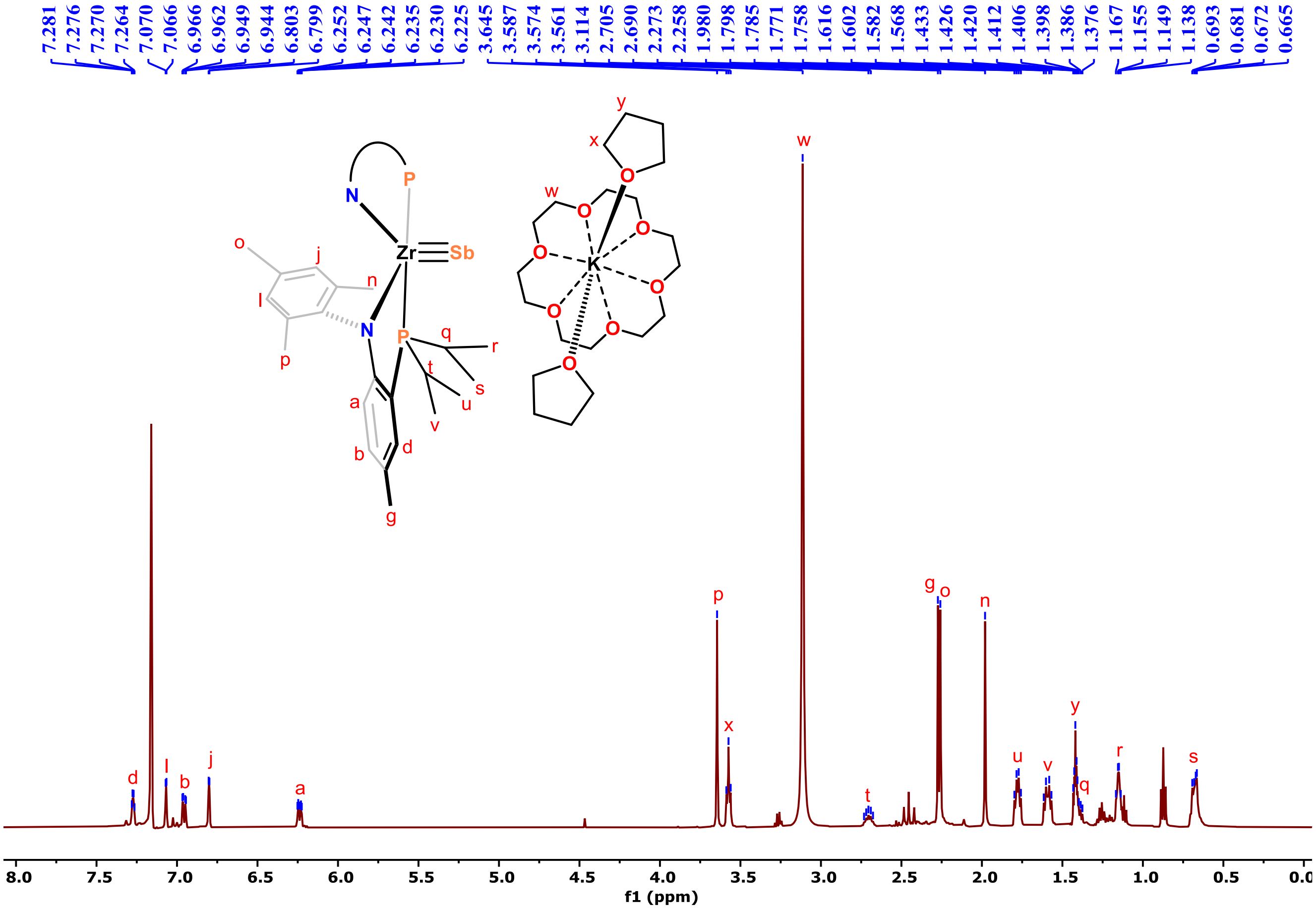

Figure S1: ^{1}H NMR spectrum of **2** in benzene–d_6, 500 MHz, 298 K. The resonances at 7.32, and 7.23 ppm corresponds as satellite peaks for the resonance at 7.27 ppm. Furthermore, the resonances at 7.13, 7.02, and 2.11 (toluene) ppm; 4.47 (dihydrogen) ppm; 3.27, 1.12 (OEt_2) ppm; 1.25, and 0.87 (pentane) ppm corresponds to residual solvent.[24] The resonances at 6.99–6.98, 6.21–6.19, 2.46, 1.39–1.38, and 1.22–1.19 ppm correspond to trace amount of unidentified impurities.

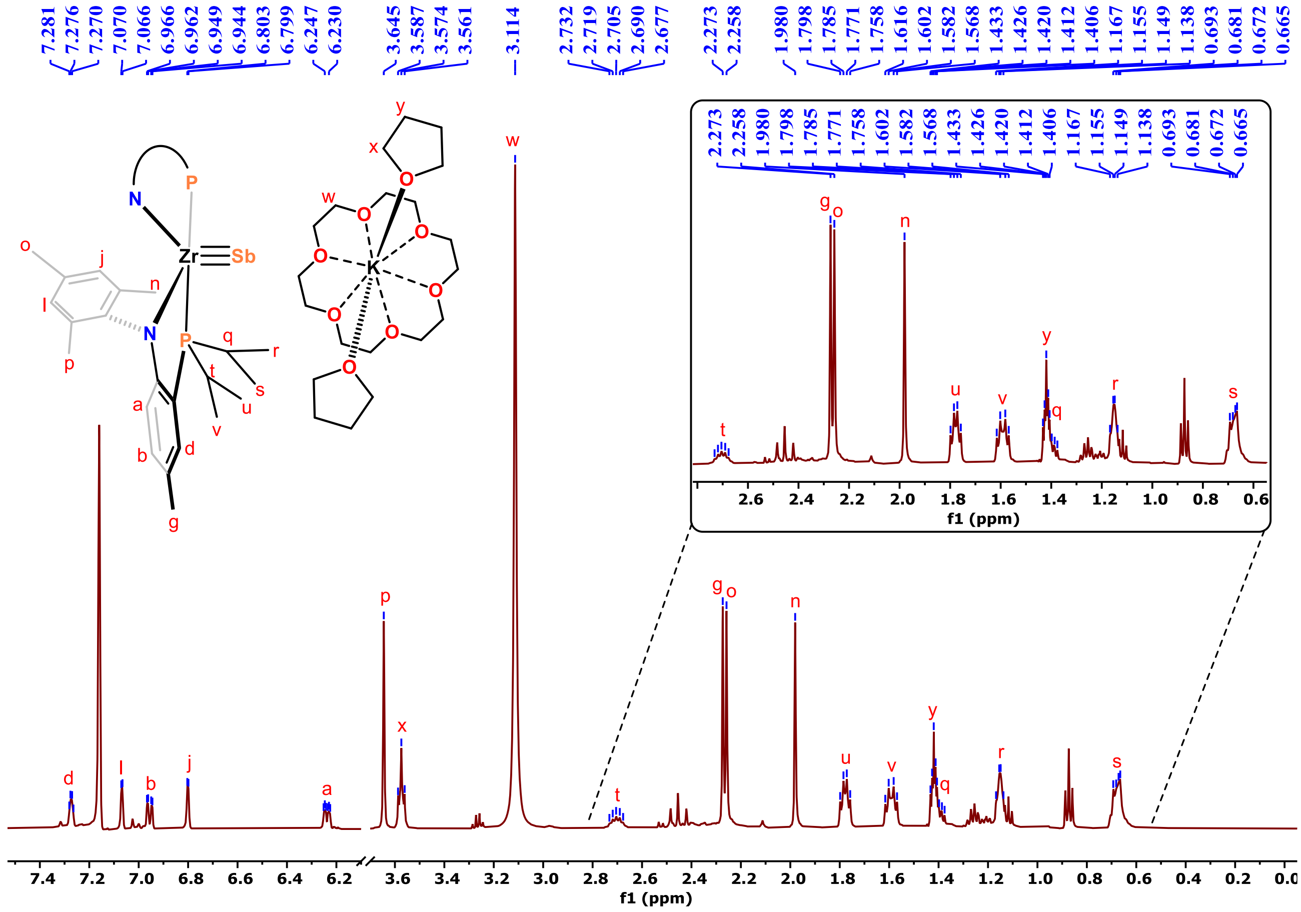

Figure S2: ^{1}H NMR spectrum of **2** in benzene–d_6, 500 MHz, 298 K. Inset: zoom region from 2.80–0.60 ppm for clear assignments of all the salient proton resonances. The resonances at 7.32, and 7.23 ppm corresponds as satellite peaks for the resonance at 7.27 ppm. Furthermore, the resonances at 7.13, 7.02, and 2.11 (toluene) ppm; 3.27, 1.12 (OEt_2) ppm; 1.25, and 0.87 (pentane) ppm corresponds to residual solvent.[24] The resonances at 6.99–6.98, 6.21–6.19, 2.46, 1.39–1.38, and 1.22–1.19 ppm correspond to trace amount of unidentified impurities.

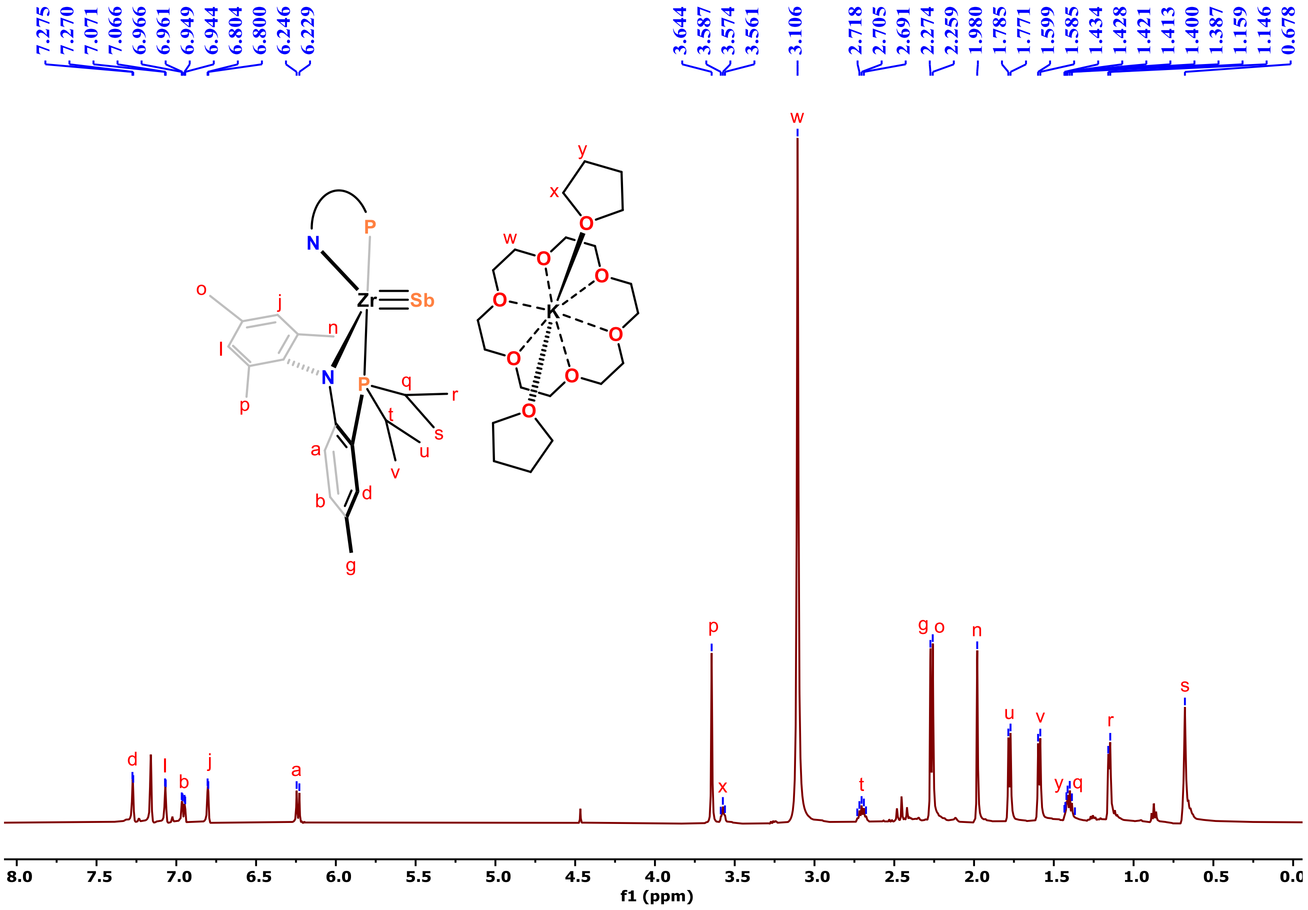

Figure S3: $^{1}H\{^{31}P\}$ NMR spectrum of **2** in benzene–d_6, 500 MHz, 298 K. The resonances at 7.31, and 7.23 ppm corresponds as satellite peaks for the resonance at 7.27 ppm. Moreover, the resonances at 7.12, 7.02, and 2.12 (toluene) ppm; 4.47 (dihydrogen) ppm; 3.27, 1.12 (OEt_2) ppm; 1.25, and 0.87 (pentane) ppm corresponds to residual solvent.[24] The resonances at 6.21–6.19, 2.45, and 1.22–1.19 ppm correspond to trace amount of unidentified impurities.

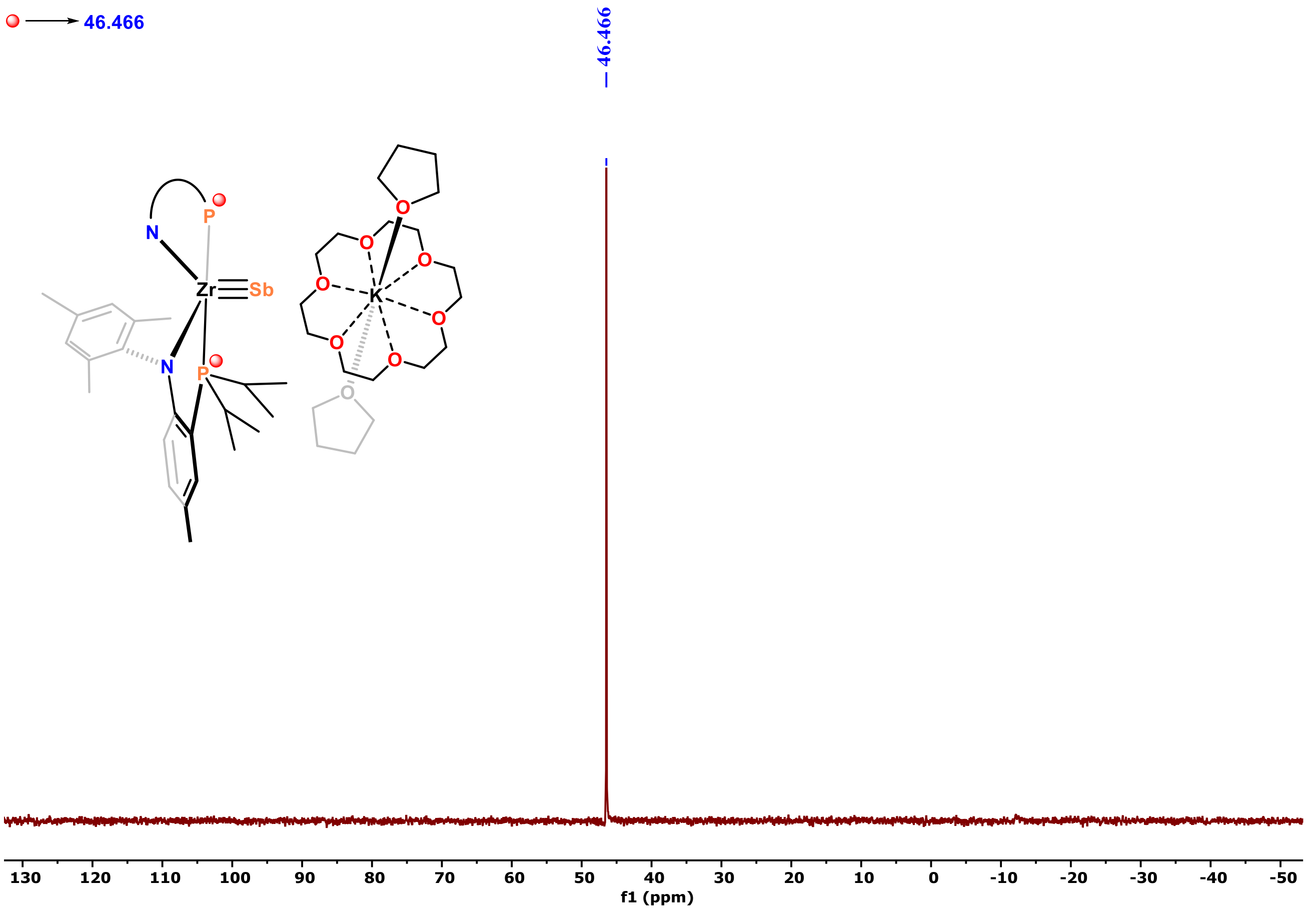

Figure S4: $^{31}P\{^{1}H\}$ NMR spectrum of **2** in benzene–d_6, 202.5 MHz, 298 K.

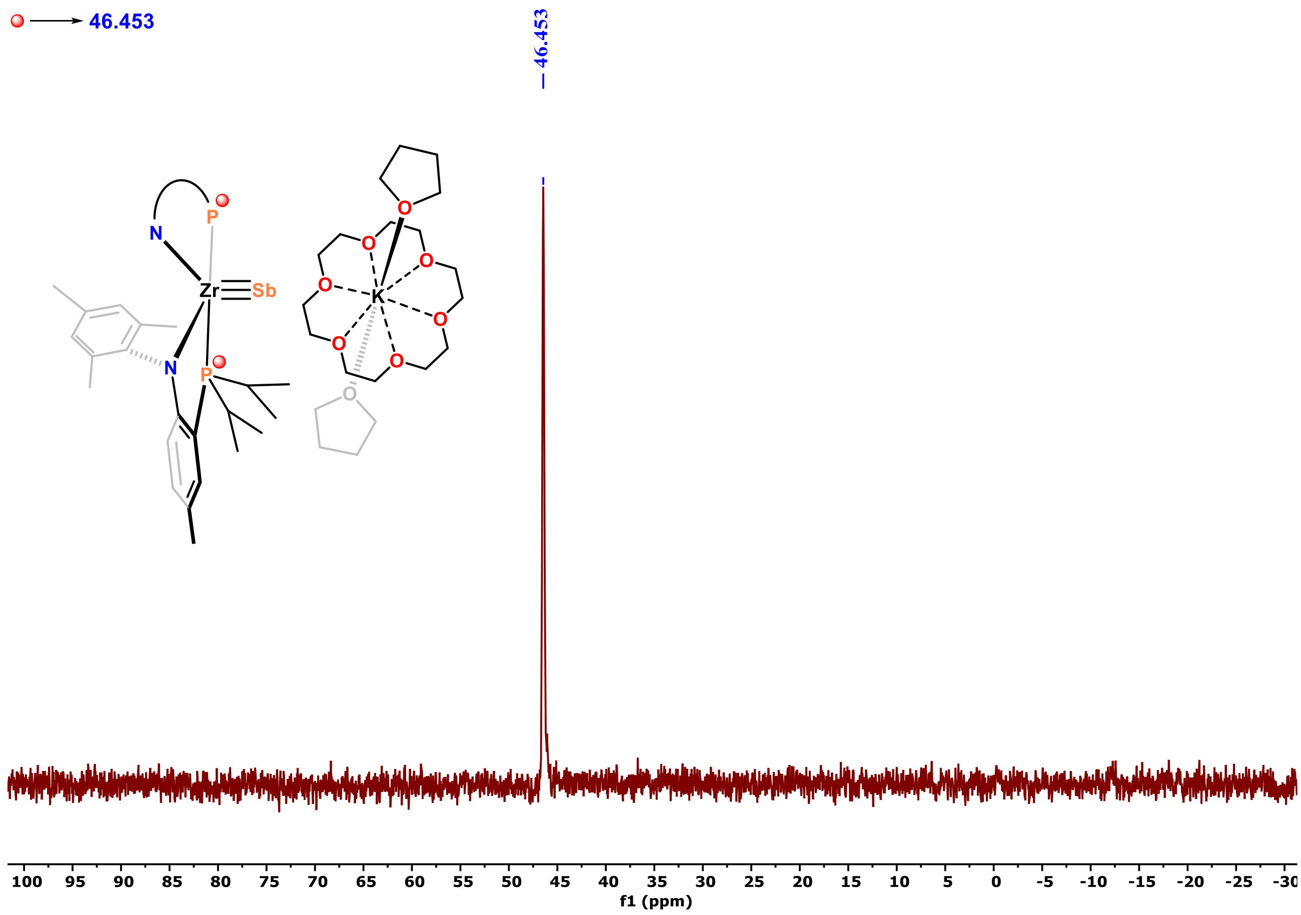

Figure S5: ^{31}P NMR spectrum of **2** in benzene–d_6, 202.5 MHz, 298 K.

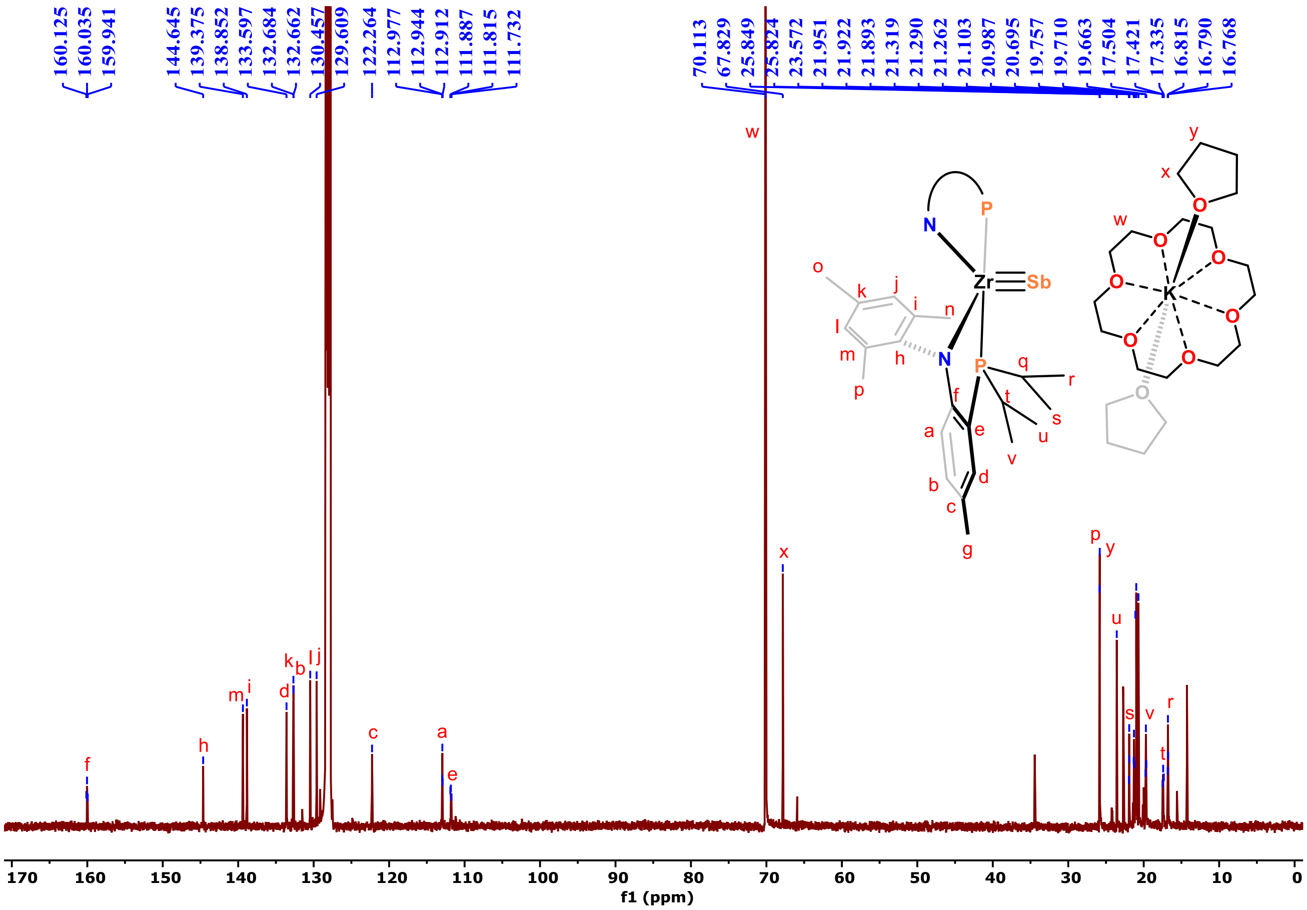

Figure S6: $^{13}C\{^{1}H\}$ NMR spectrum of **2** in benzene–d_6, 125.8 MHz, 298 K. The resonances at 65.9, and 15.6 (OEt_2) ppm; 34.4, 22.7, and 14.3 (pentane) ppm corresponds to residual solvent molecules.[24] Also, the resonances at 131.5, 129.1, 127.5, 24.2, 24.1, 21.4, 20.2, 20.1, and 20.0 ppm indicate trace amount of unidentified impurities.

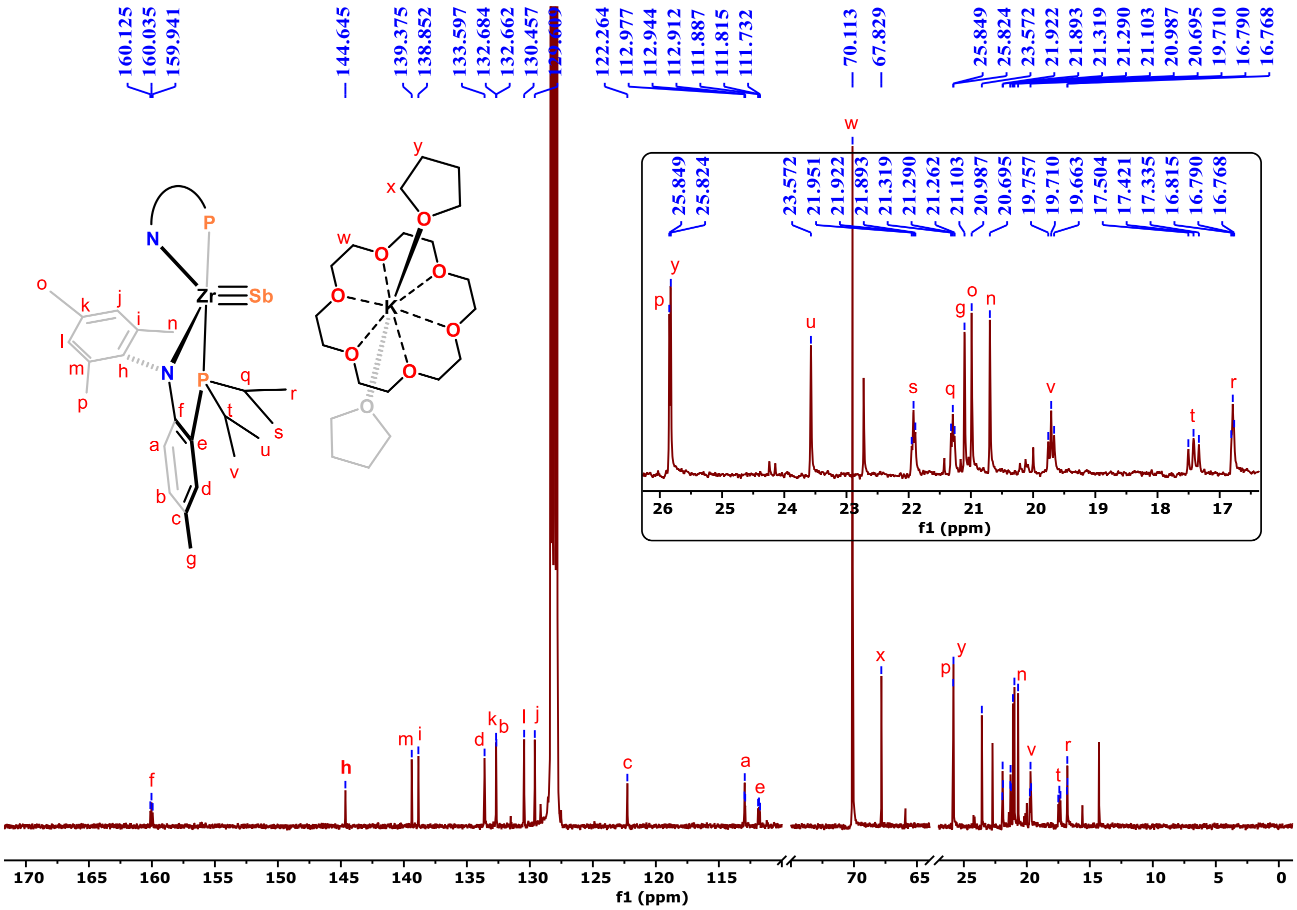

Figure S7: $^{13}C\{^{1}H\}$ NMR spectrum of **2** in benzene–d_6, 125.8 MHz, 298 K from the region of 170.0–110.0, 80.0–65.0, and 27.5–0.0 ppm. Inset: zoom region from 26.0–16.5 ppm for clear assignments of all the salient carbon resonances. The resonances at 65.9, and 15.6 (OEt_2) ppm; 22.7, and 14.3 (pentane) ppm corresponds to residual solvent molecules.[24] Also, the resonances at 131.5, 129.1, 127.5, 24.2, 24.1, 21.4, 20.2, 20.1, and 20.0 ppm indicate trace amount of unidentified impurities.

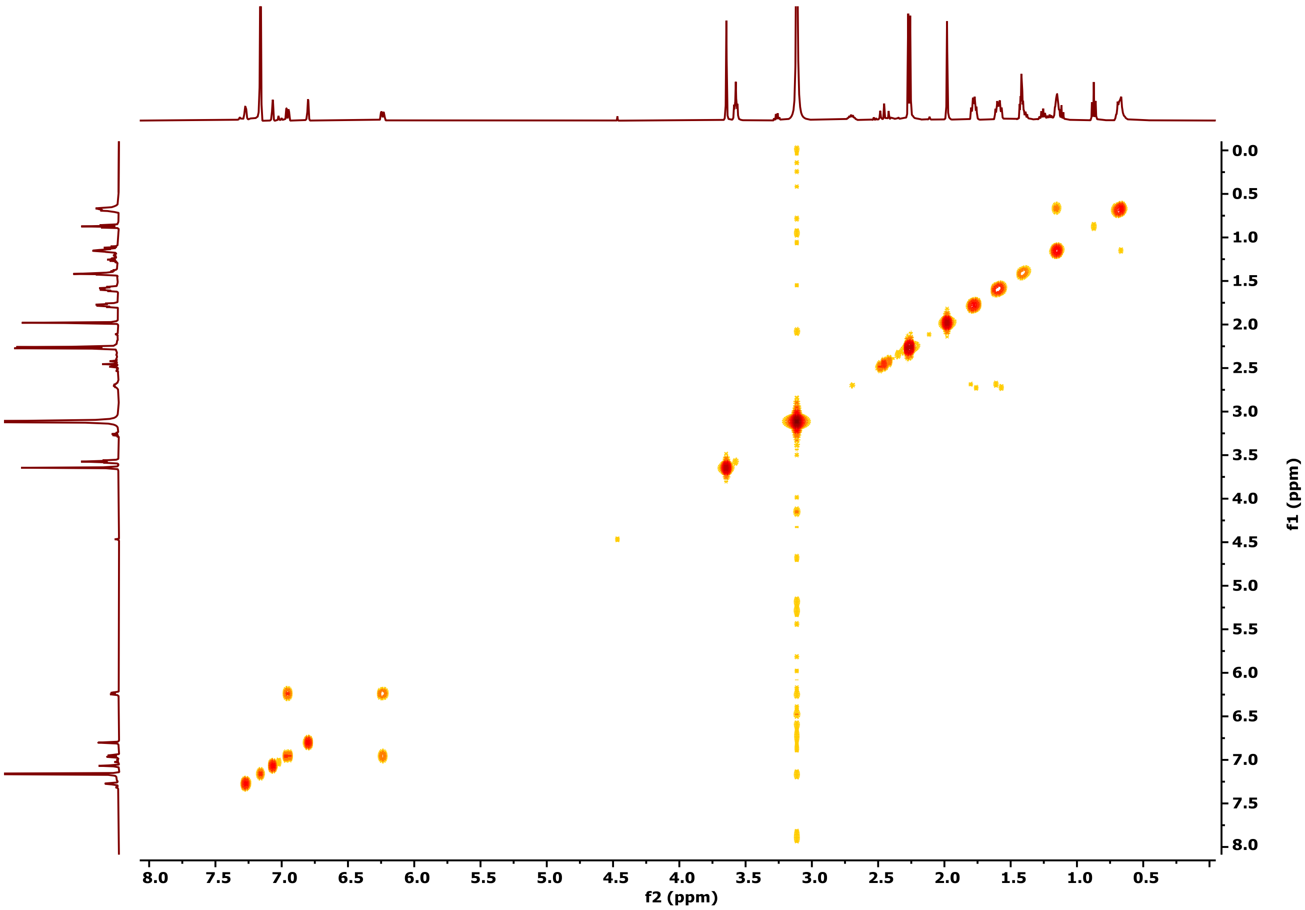

Figure S8: ^{1}H-^{1}H COSY NMR spectrum of **2** in benzene–d_6 (298 K).

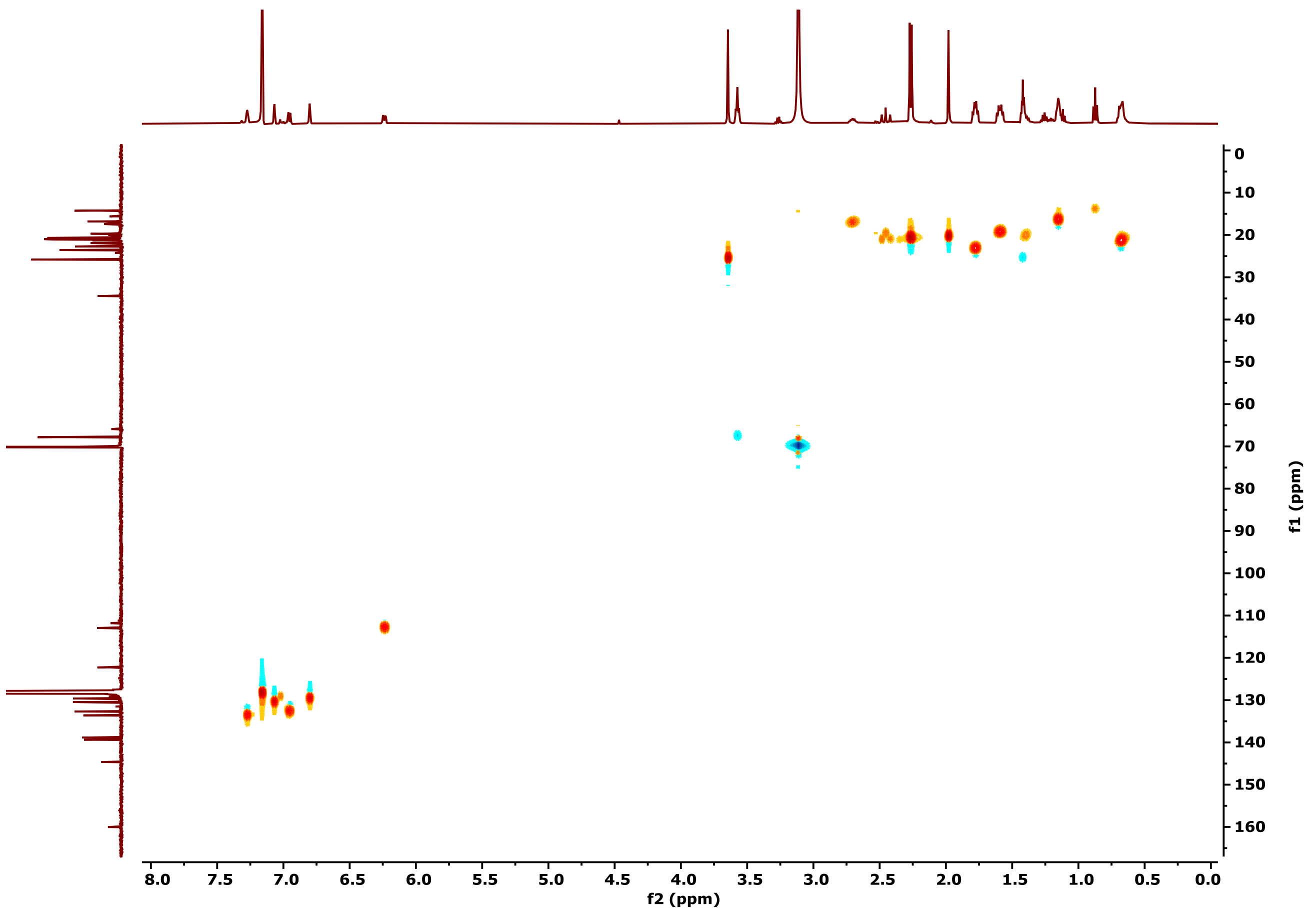

Figure S9: ^{1}H-^{13}C HSQC NMR spectrum of **2** in benzene–d_6 (298 K).

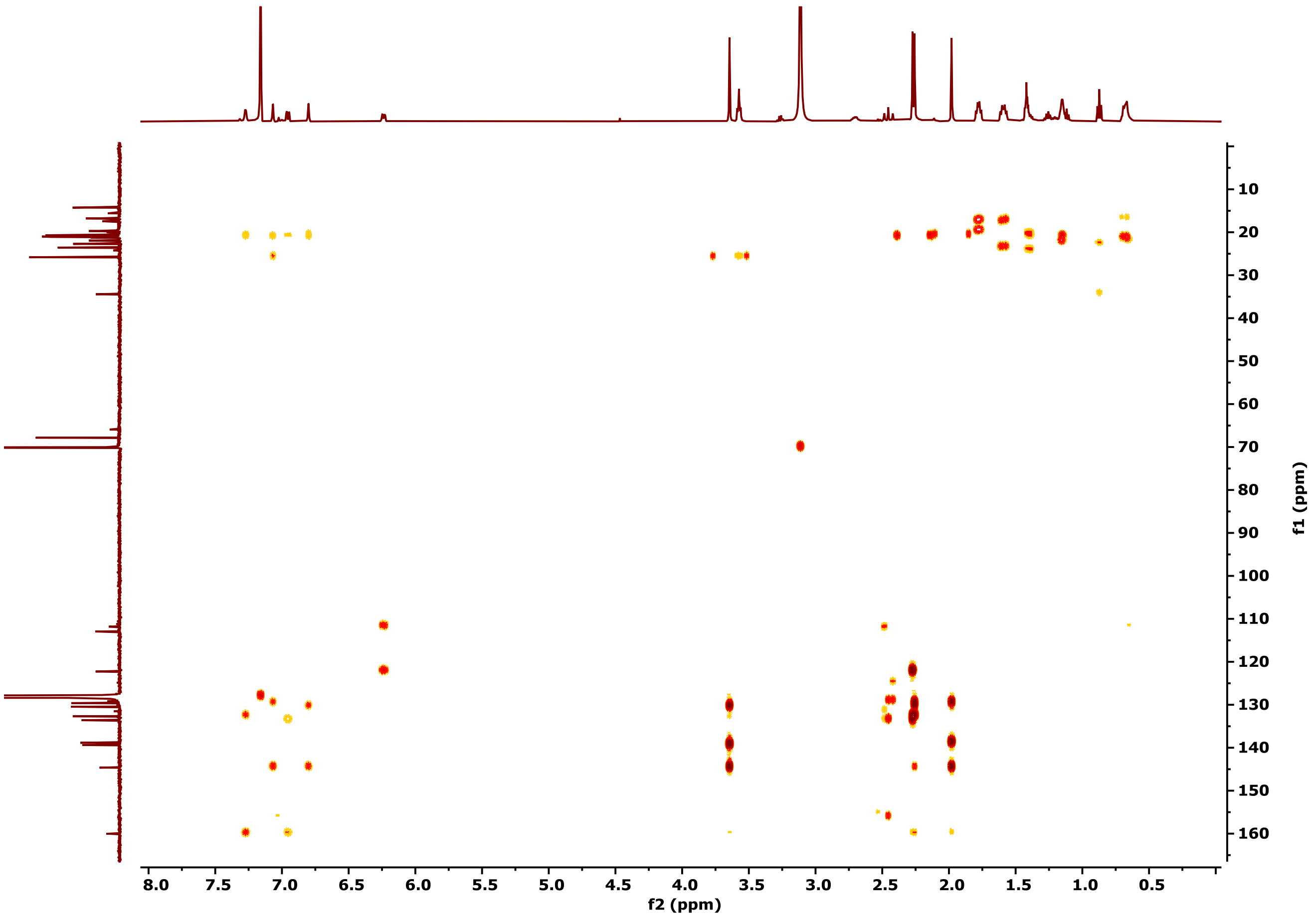

Figure S10: ^{1}H-^{13}C HMBC NMR spectrum of **2** in benzene–d_6 (298 K).

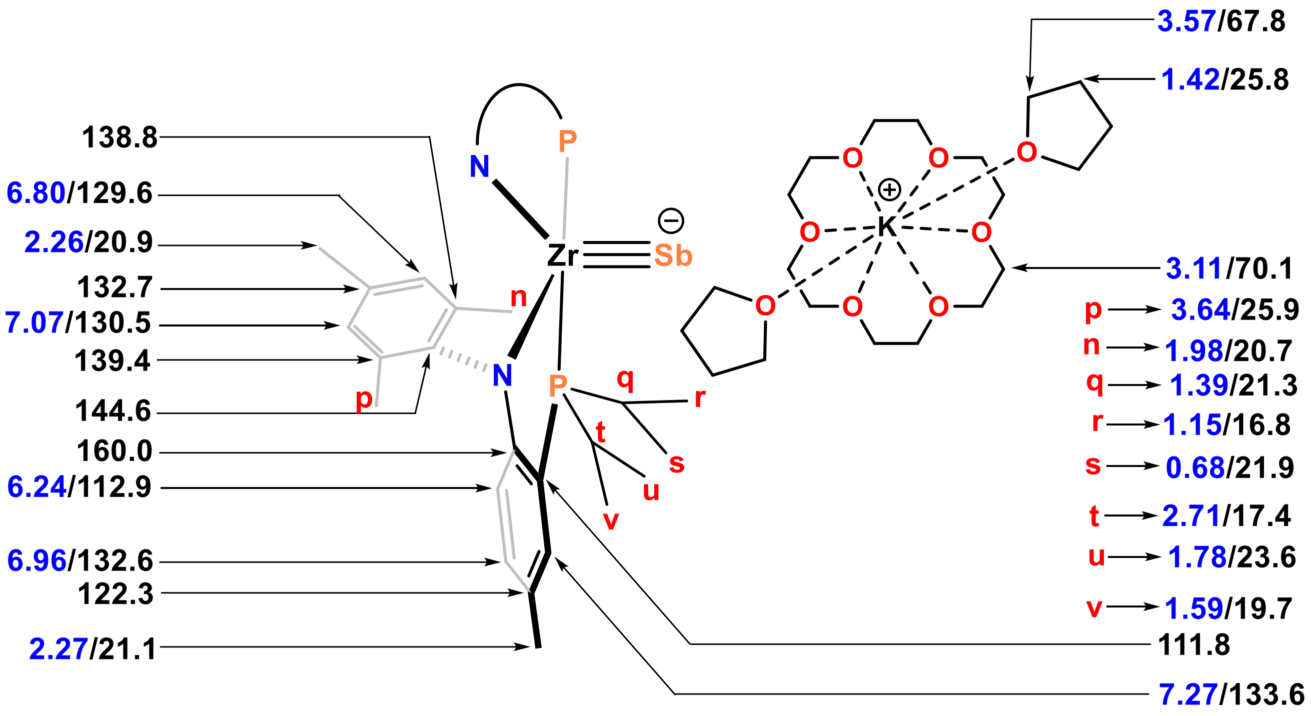

Figure S11: ^{1}H (blue), and $^{13}C\{^{1}H\}$ (black) NMR spectral assignments for **2**.

4.2 NMR Spectral Data for [K(18-C-6)(THF)$_2$][(PN)$_2$Zr≡As] (3)

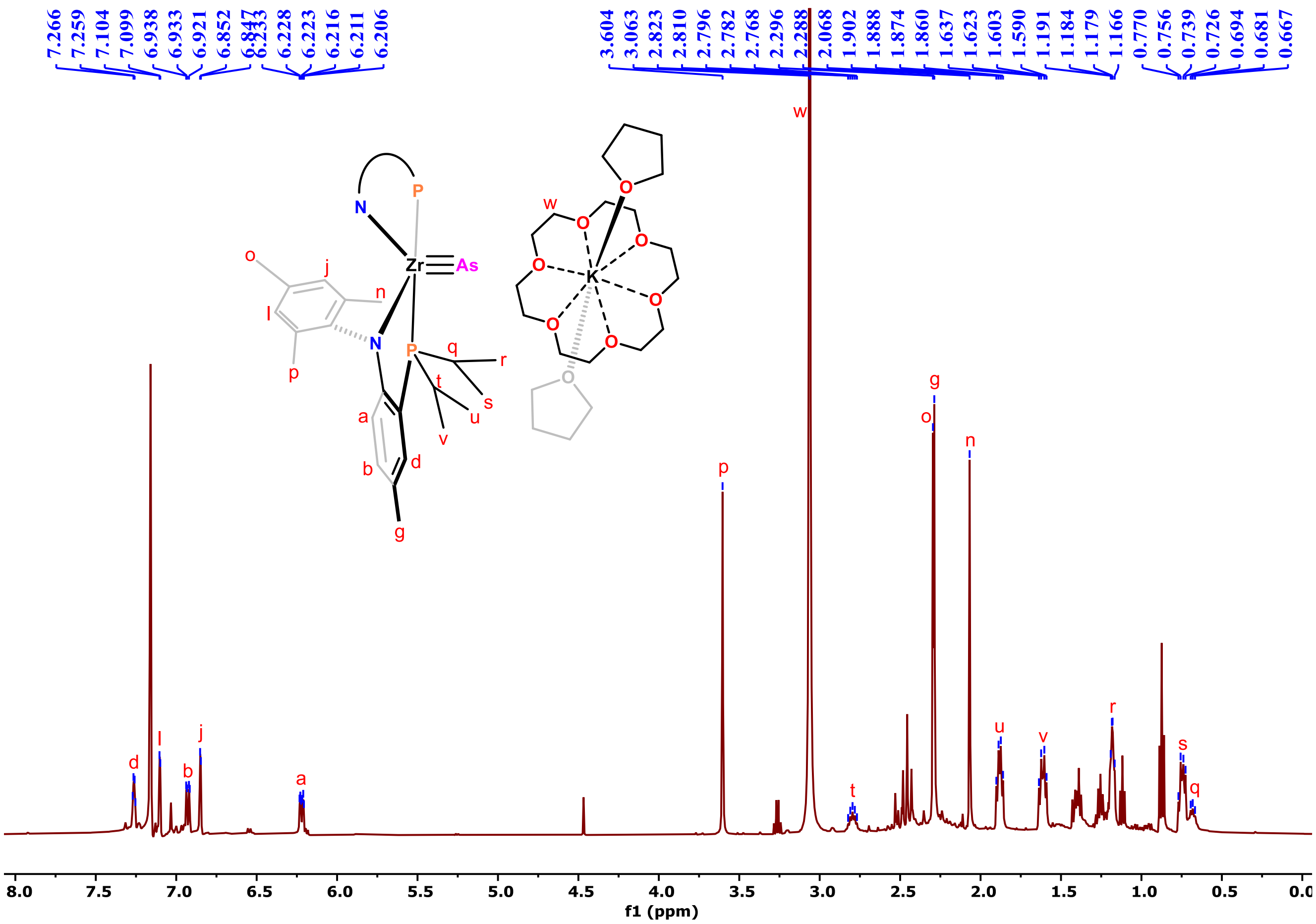

Figure S12: ^{1}H NMR spectrum of **3** in benzene–d_6, 500 MHz, 298 K. The resonances at 7.32, and 7.23 ppm corresponds as satellite peaks for the resonance at 7.26 ppm. Furthermore, the resonances at 7.13, 7.03, and 2.11 (toluene) ppm; 4.47 (dihydrogen) ppm; 3.26, 1.12 (OEt_2) ppm; 1.25, and 0.87 (pentane) ppm corresponds to residual solvent.[24] The resonances at 7.00–6.97, 6.55–6.54, 6.19–6.18, 2.53–2.43, and 1.43−1.37 ppm correspond to trace amount of unidentified impurities. The labile co-crystallized THF was not observed in ^{1}H NMR spectroscopy.

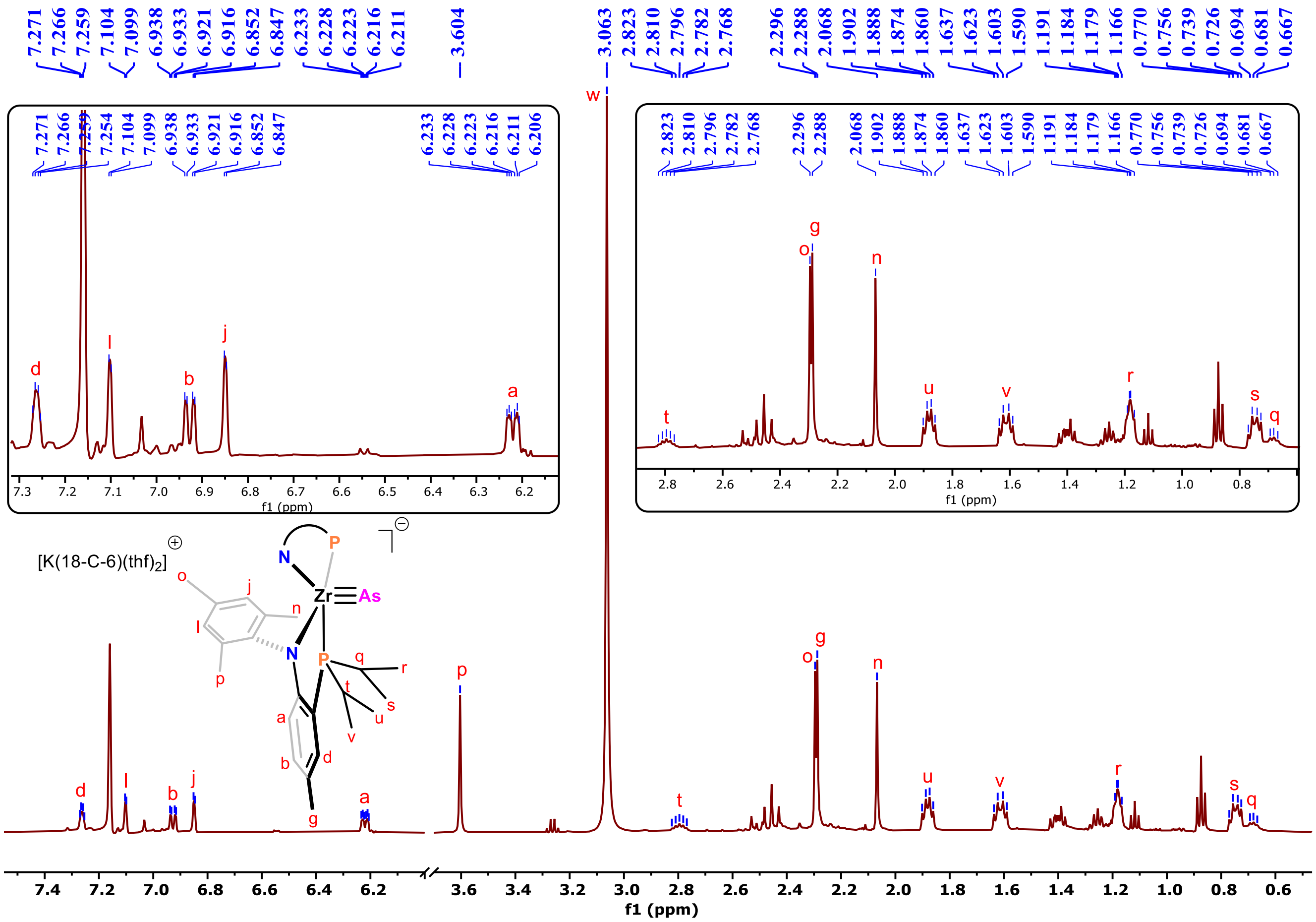

Figure S13: ^1H NMR spectrum of **3** in benzene–d_6, 500 MHz, 298 K, from region 7.5–6.0 and 3.7–0.5 ppm. Inset: zoom region from 7.30–6.15, and 2.80–0.60 ppm for clear assignments of all the salient proton resonances. The resonances at 7.32, and 7.23 ppm corresponds as satellite peaks for the resonance at 7.26 ppm. Also, the resonances at 7.13, 7.03, and 2.11 (toluene) ppm; 3.26, 1.12 (OEt_2) ppm; 1.25, and 0.87 (pentane) ppm corresponds to residual solvent.[24] The resonances at 7.00–6.97, 6.55–6.54, 6.19–6.18, 2.53–2.43, and 1.43–1.37 ppm correspond to trace amount of unidentified impurities. The labile co-crystallized THF was not observed in ^1H NMR spectroscopy.

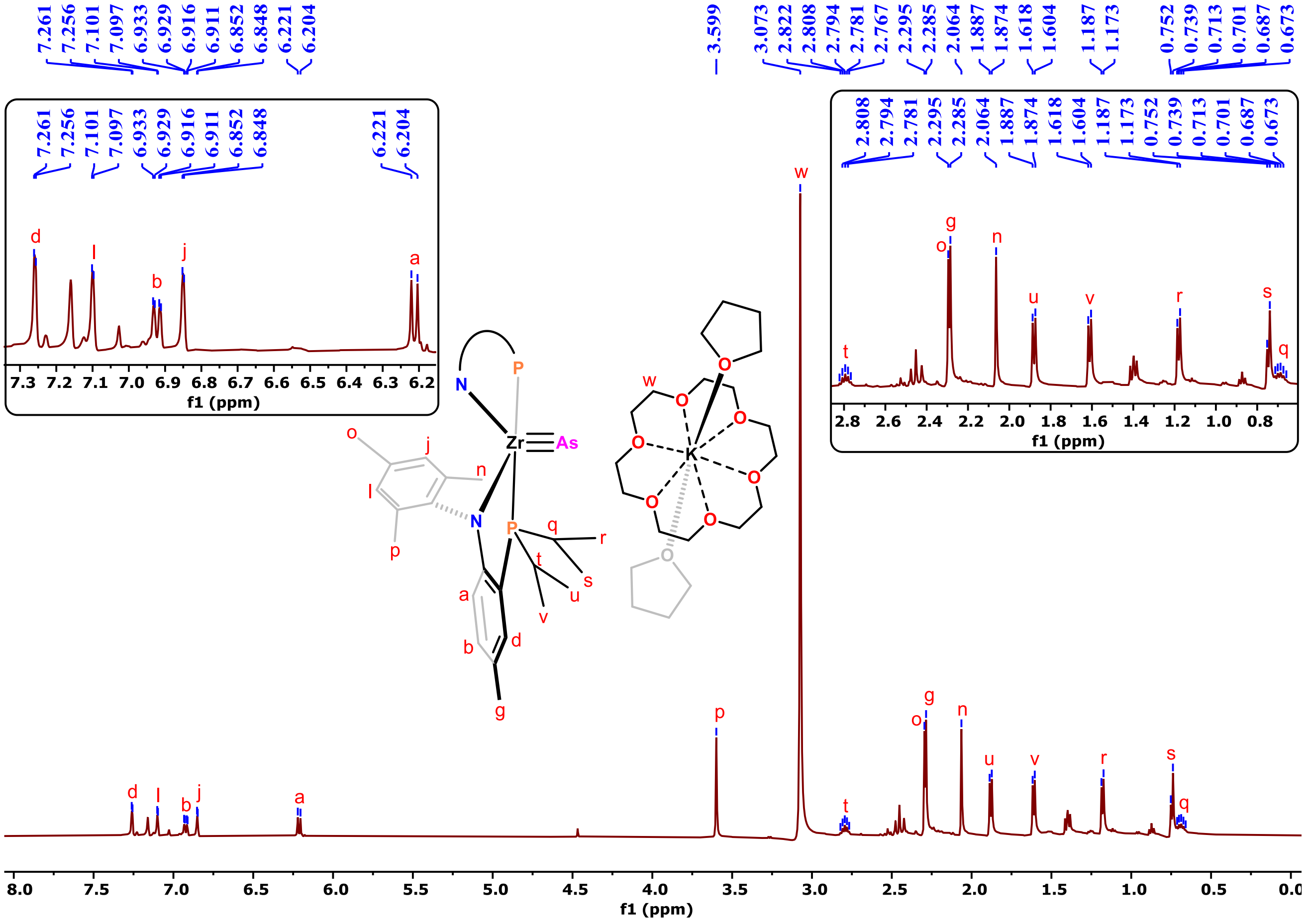

Figure S14: $^{1}H\{^{31}P\}$ NMR spectrum of **3** in benzene–*d*6, 500 MHz, 298 K. Inset: zoom region from 7.35–6.15, and 2.83–0.60 ppm for clear assignments of all the salient proton resonances. The resonances at 7.32, and 7.23 ppm corresponds as satellite peaks for the resonance at 7.26 ppm. Also, the resonances at 7.12, 7.03, and 2.11 (toluene) ppm; 4.47 (dihydrogen) ppm; 1.25, and 0.87 (pentane) ppm corresponds to residual solvent.[24] The resonances at 7.00–6.97, 6.55–6.53, 6.19–6.18, 2.53–2.42, and 1.40 ppm correspond to trace amount of unidentified impurities. The labile co-crystallized THF was not observed in $^{1}H\{^{31}P\}$ NMR spectroscopy.

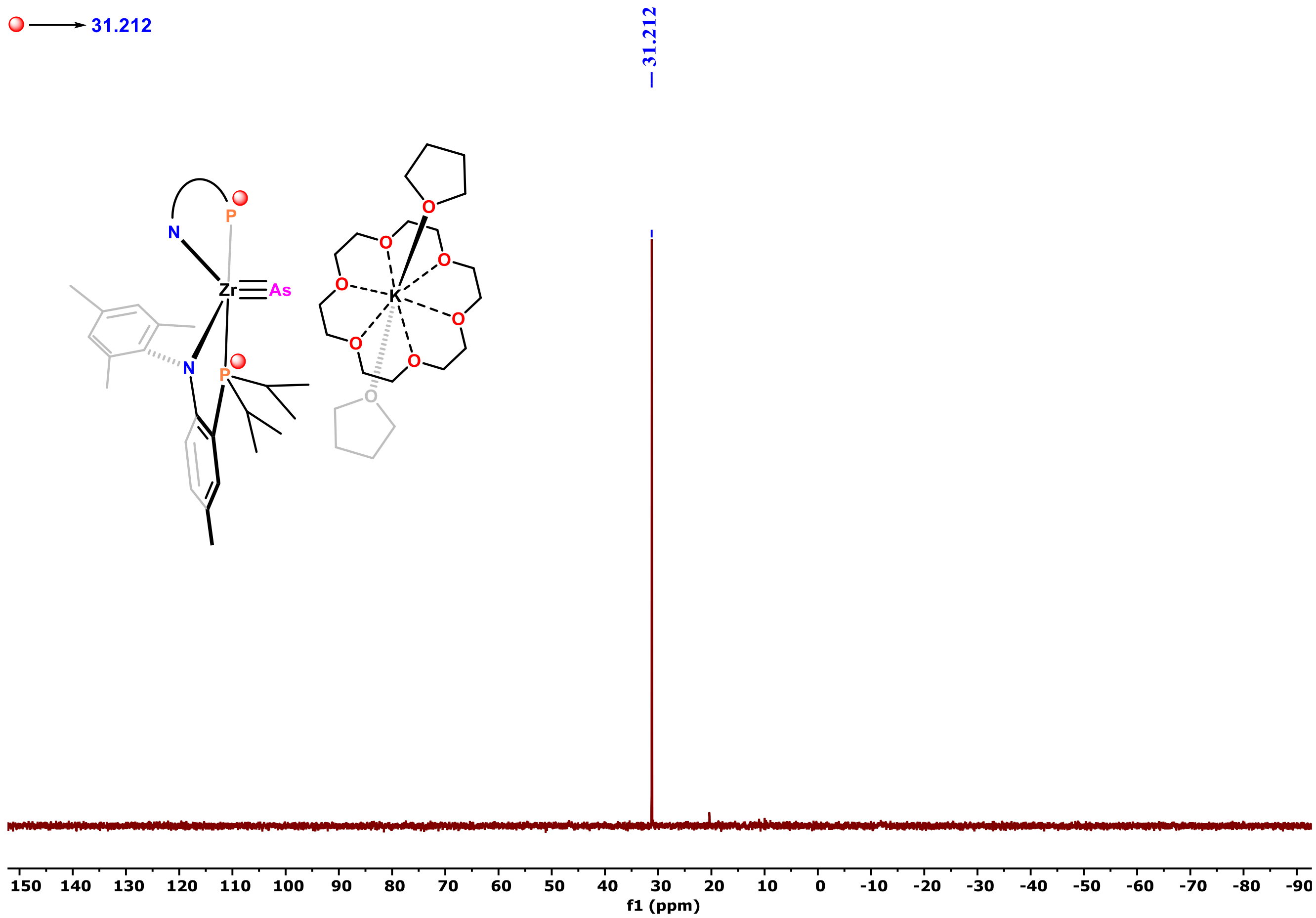

Figure S15: $^{31}P\{^1H\}$ NMR spectrum of **3** in benzene–d_6, 202.5 MHz, 298 K.

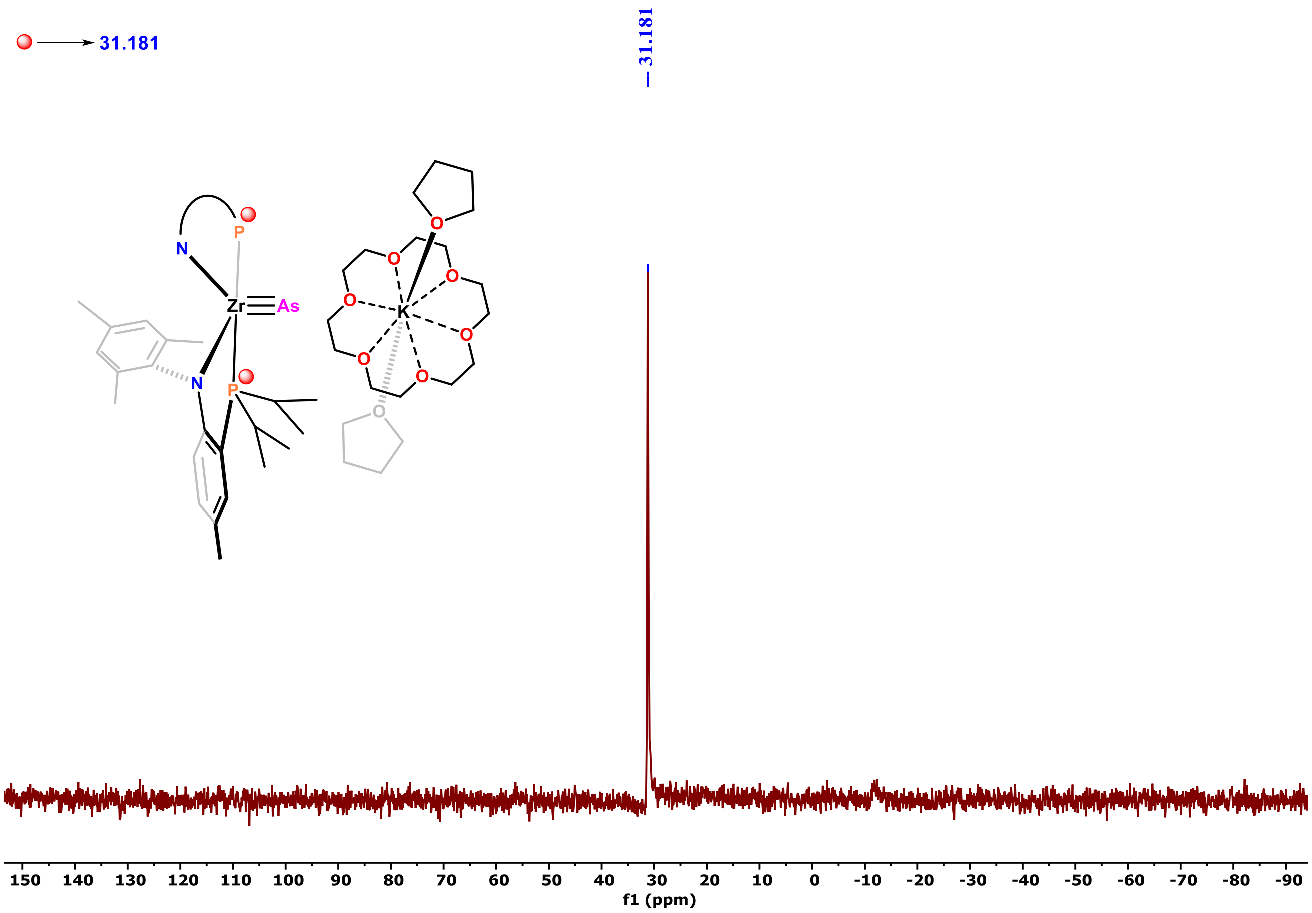

Figure S16: ^{31}P NMR spectrum of **3** in benzene–d_6, 202.5 MHz, 298 K.

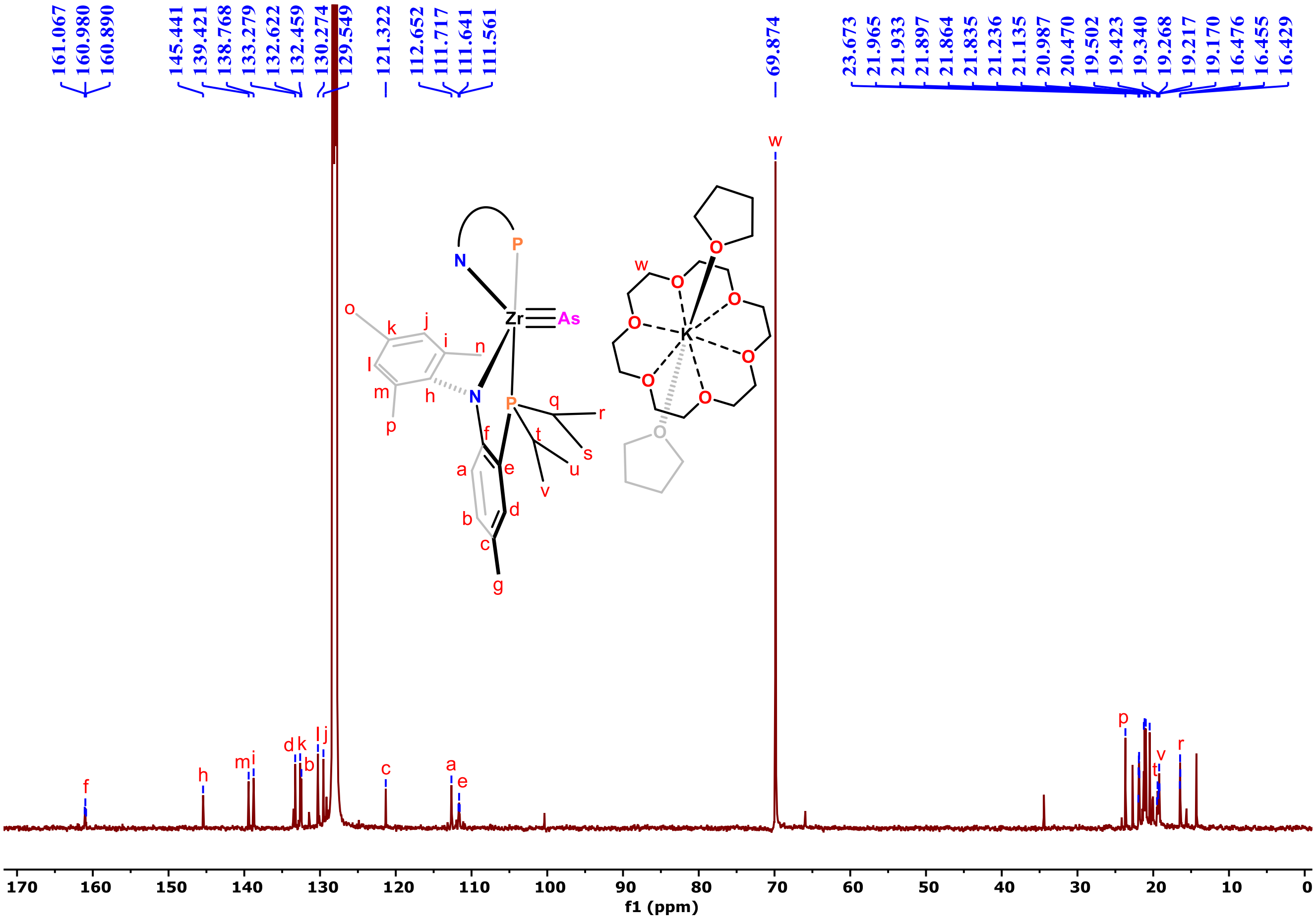

Figure S17: $^{13}C\{^{1}H\}$ NMR spectrum of **3** in benzene–d_6, 125.8 MHz, 298 K. The resonances at 137.7, 129.3, 128.6, 125.7, and 21.1 (toluene); 65.9, and 15.6 (OEt_2) ppm; 34.4, 22.7, and 14.3 (pentane) ppm corresponds to residual solvent molecules.[24] Also, the resonances at 133.5, 132.8, 131.4, 130.1, 129.8, 128.9, 127.5, 124.9, 100.3, 24.2, 21.5, 21.3, 21.0, 20.2–20.0 ppm indicate trace amount of unidentified impurities. The labile co-crystallized THF was not observed in $^{13}C\{^{1}H\}$ NMR spectroscopy.

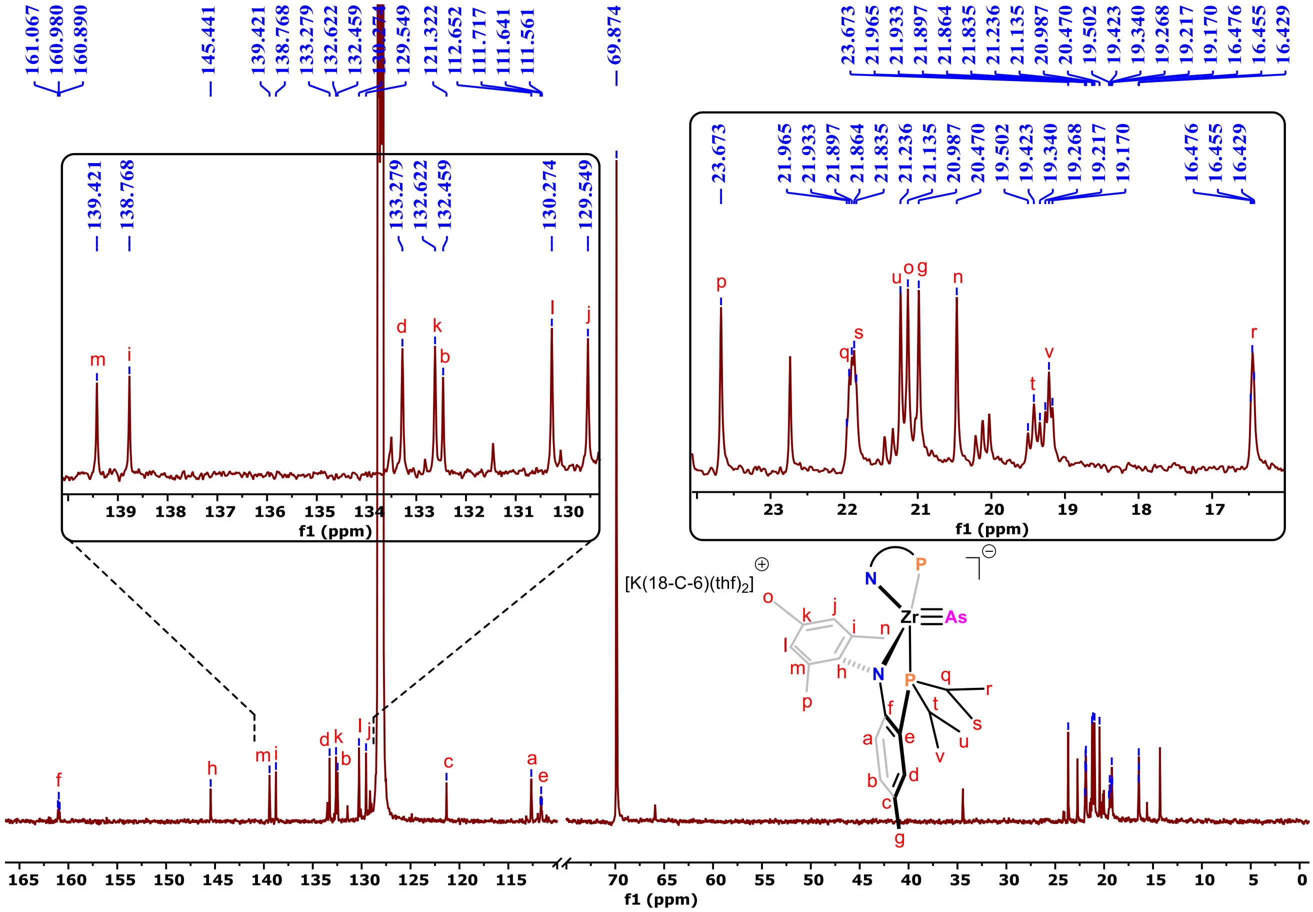

Figure S18: $^{13}C\{^{1}H\}$ NMR spectrum of **3** in benzene–d_6, 125.8 MHz, 298 K. Inset: zoom region from 140.0–129.5 and 24.0–16.0 ppm for clear assignments of all the salient carbon peaks. The resonances at 137.7, 129.3, 128.6, 125.7, and 21.1 (toluene); 65.9, and 15.6 (OEt_2) ppm; 34.4, 22.7, and 14.3 (pentane) ppm corresponds to residual solvent molecules.[24] Also, the resonances at 133.5, 132.8, 131.4, 130.1, 129.8, 128.9, 127.5, 124.9, 100.3, 24.2, 21.5, 21.3, 21.0, 20.2–20.0 ppm indicate trace amount of unidentified impurities. The labile co-crystallized THF was not observed in $^{13}C\{^{1}H\}$ NMR spectroscopy.

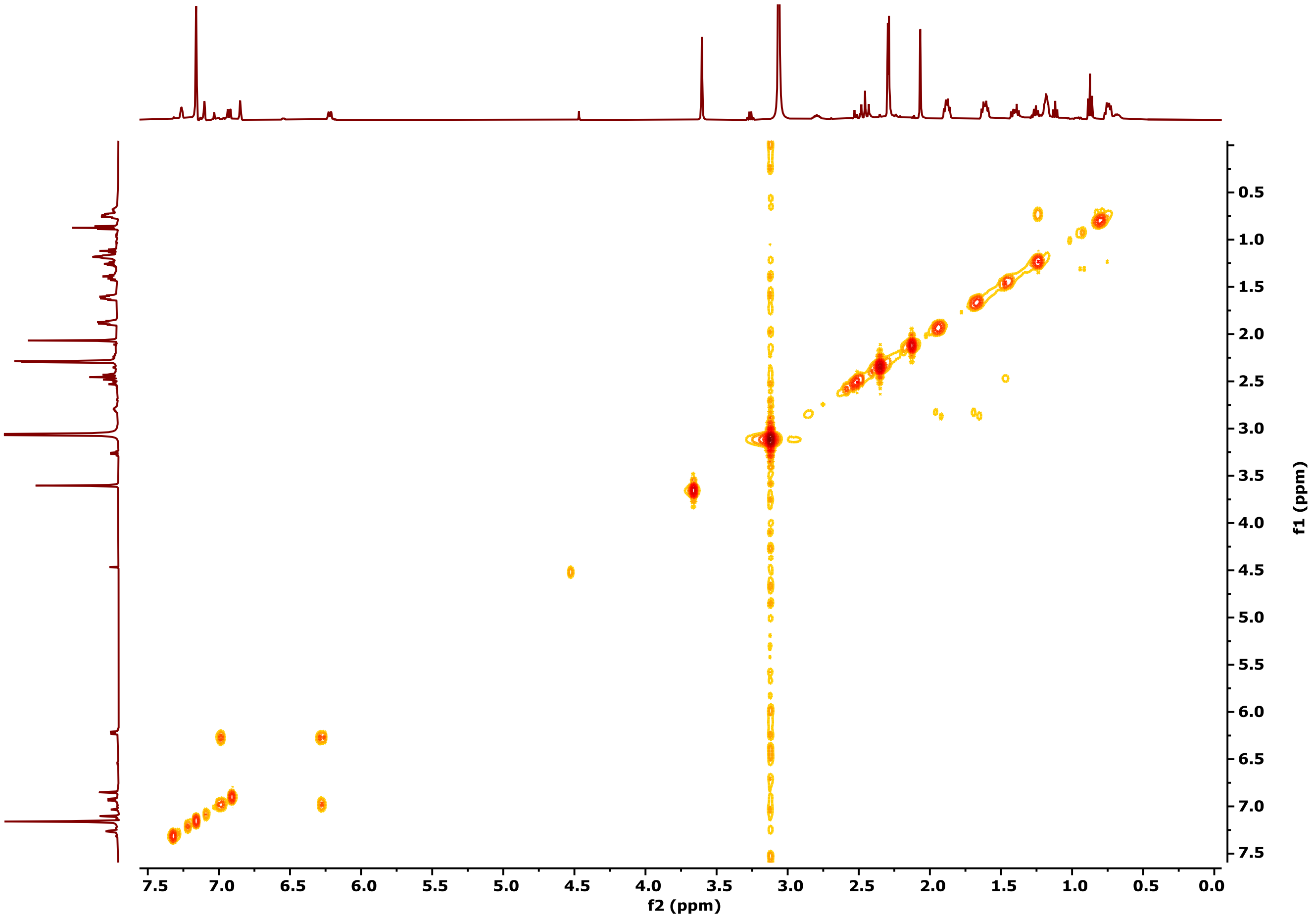

Figure S19: ^{1}H-^{1}H COSY NMR spectrum of **3** in benzene–d_6 (298 K).

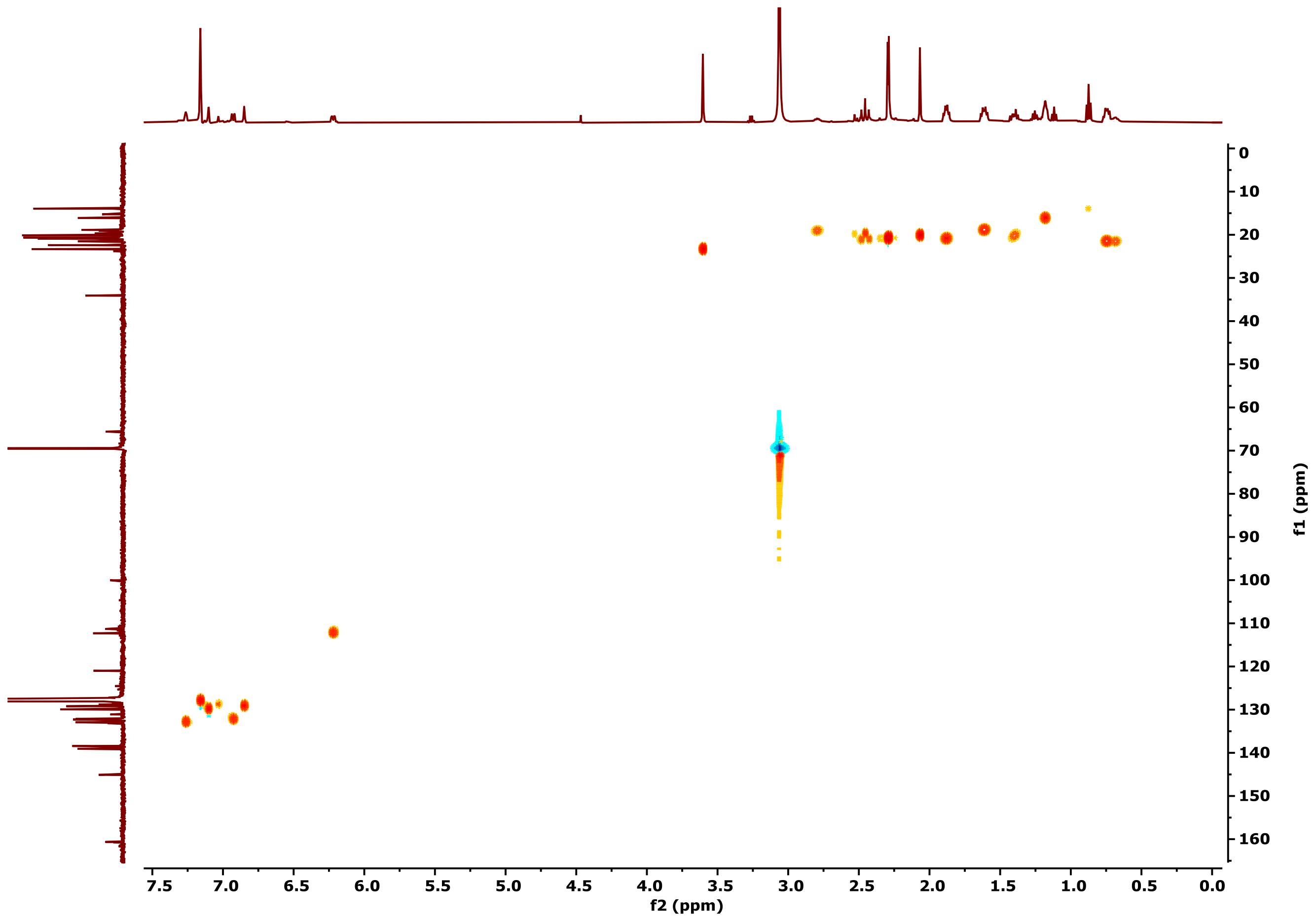

Figure S20: ^{1}H-^{13}C HSQC NMR spectrum of **3** in benzene–d_6 (298 K).

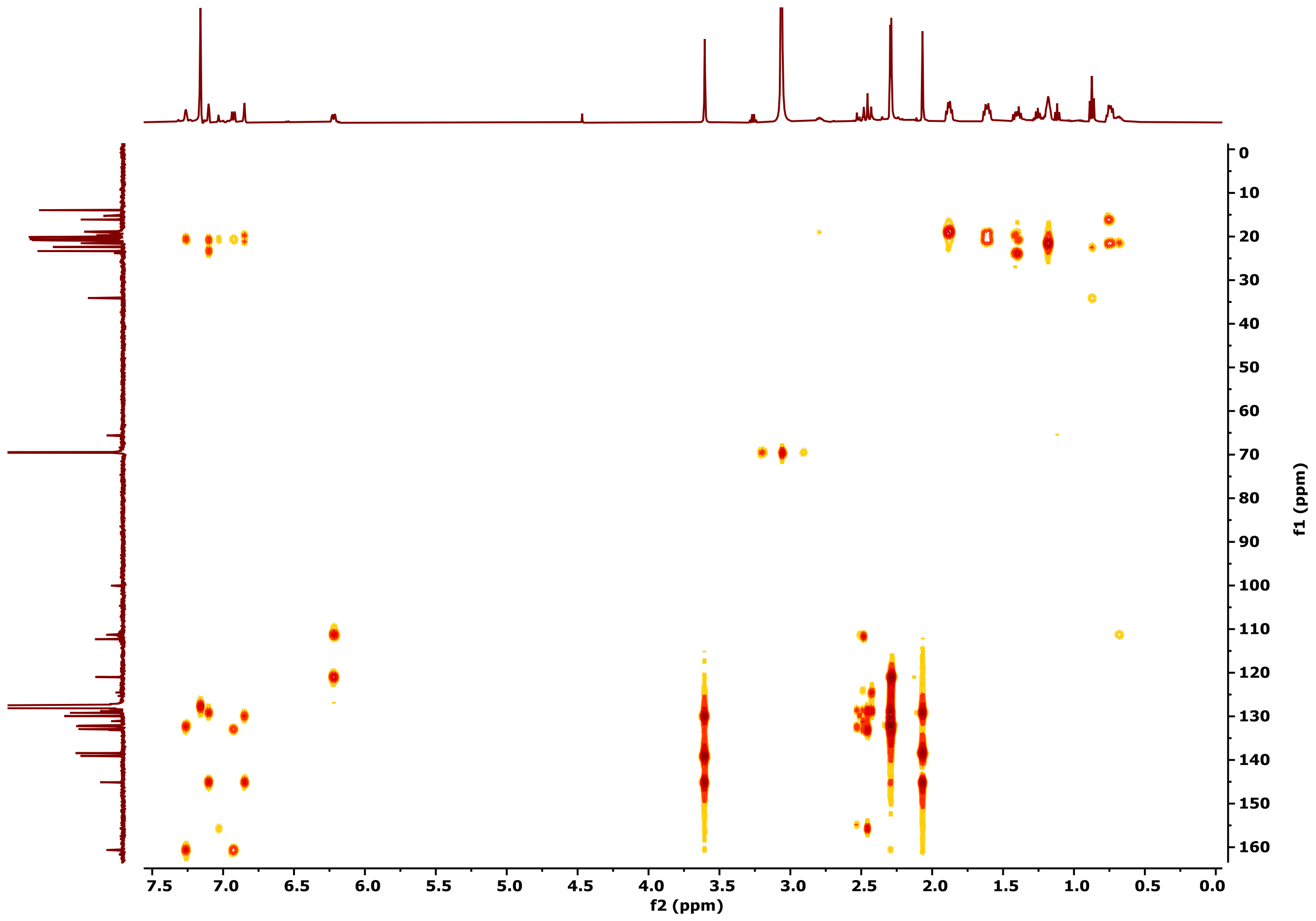

Figure S21: ^{1}H-^{13}C HMBC NMR spectrum of **3** in benzene-d_6 (298 K).

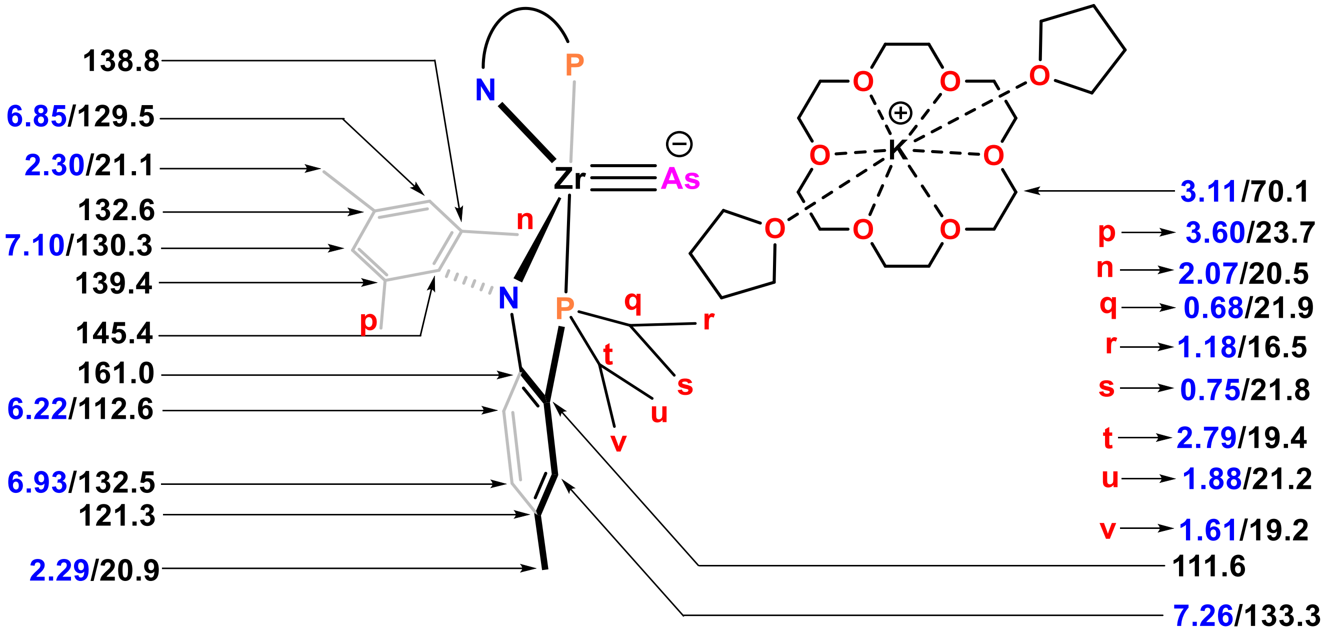

Figure S22: ^{1}H (blue), and $^{13}C\{^{1}H\}$ (black) NMR spectral assignments for **3**.

4.3 $^{31}P\{^1H\}$ NMR spectroscopy of [K(crypt)][(PN)$_2$Ti≡P] (6)

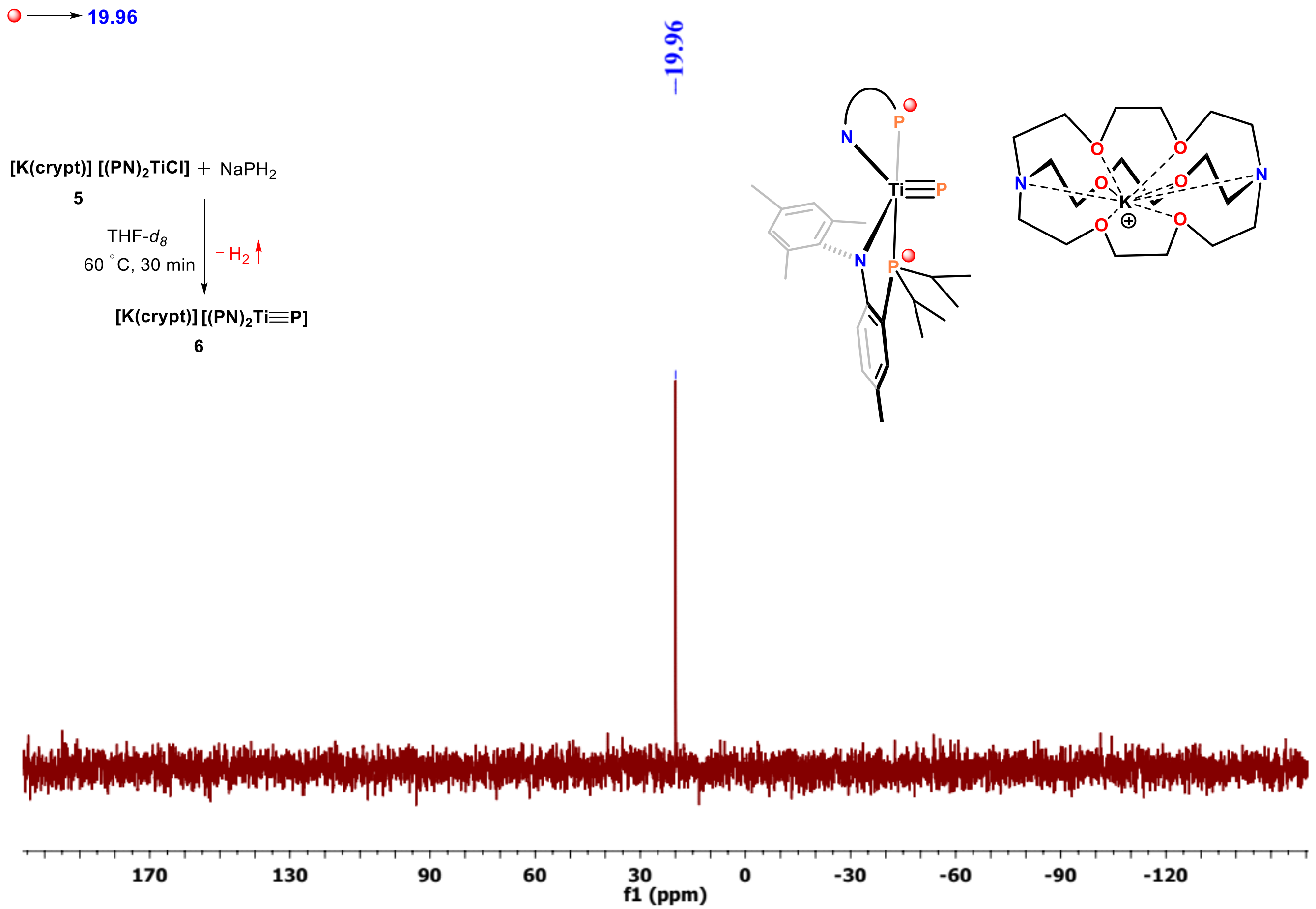

Figure S23: $^{31}P\{^1H\}$ NMR spectrum of **6** in THF−d_8, 202.5 MHz, 298 K.

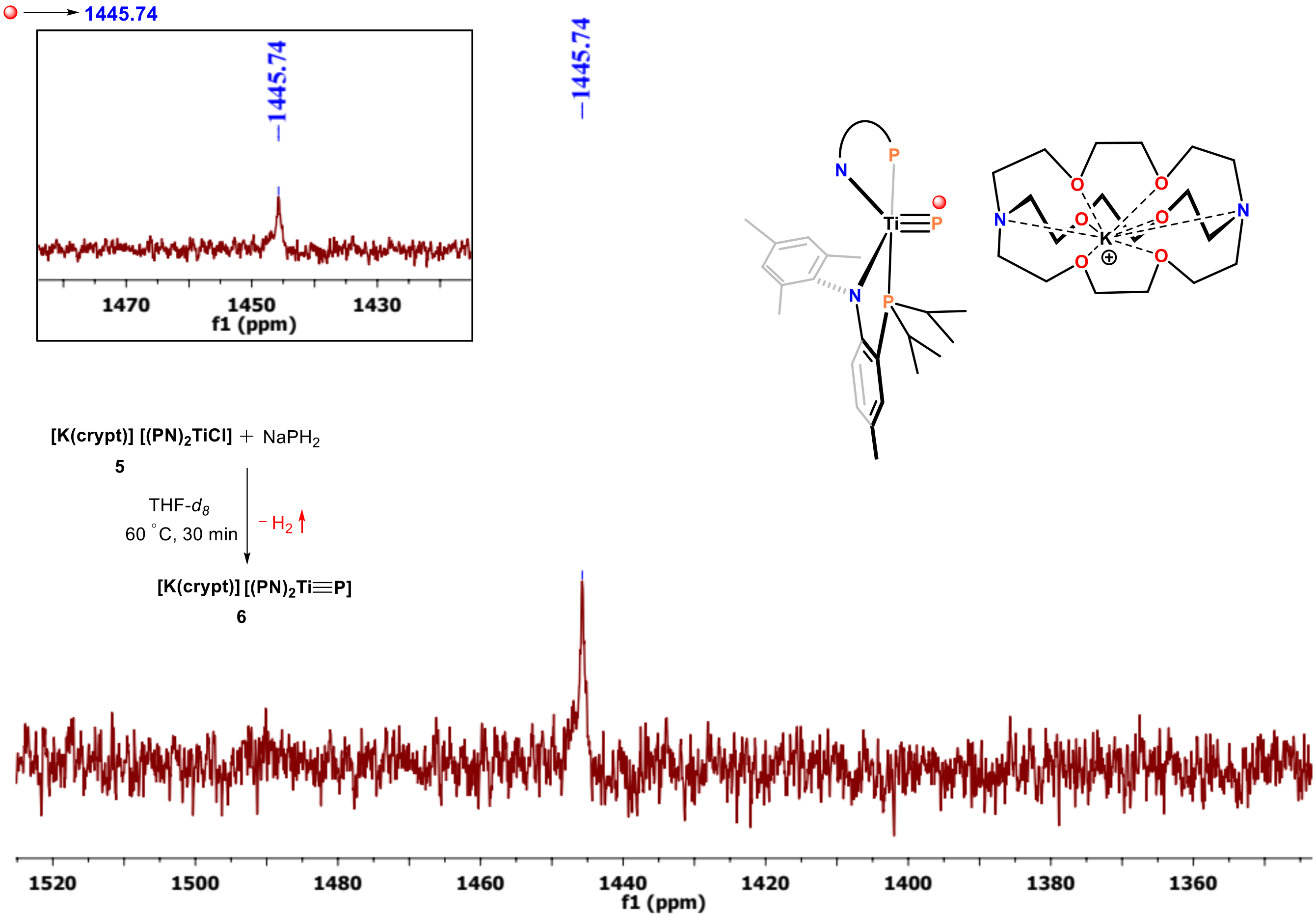

Figure S24: $^{31}P\{^{1}H\}$ NMR spectrum of **6** in THF–d_8, 202.5 MHz, 298 K. Inset: zoom region from 1415.0–1485.0 ppm to assign clearly the more downfield phosphide resonance exhibiting Ti≡P motif.

4.4 $^1H\{^{31}P\}$ and $^{31}P\{^1H\}$ NMR spectroscopy of [K(crypt)][(PN)$_2$Ti≡As] (7)

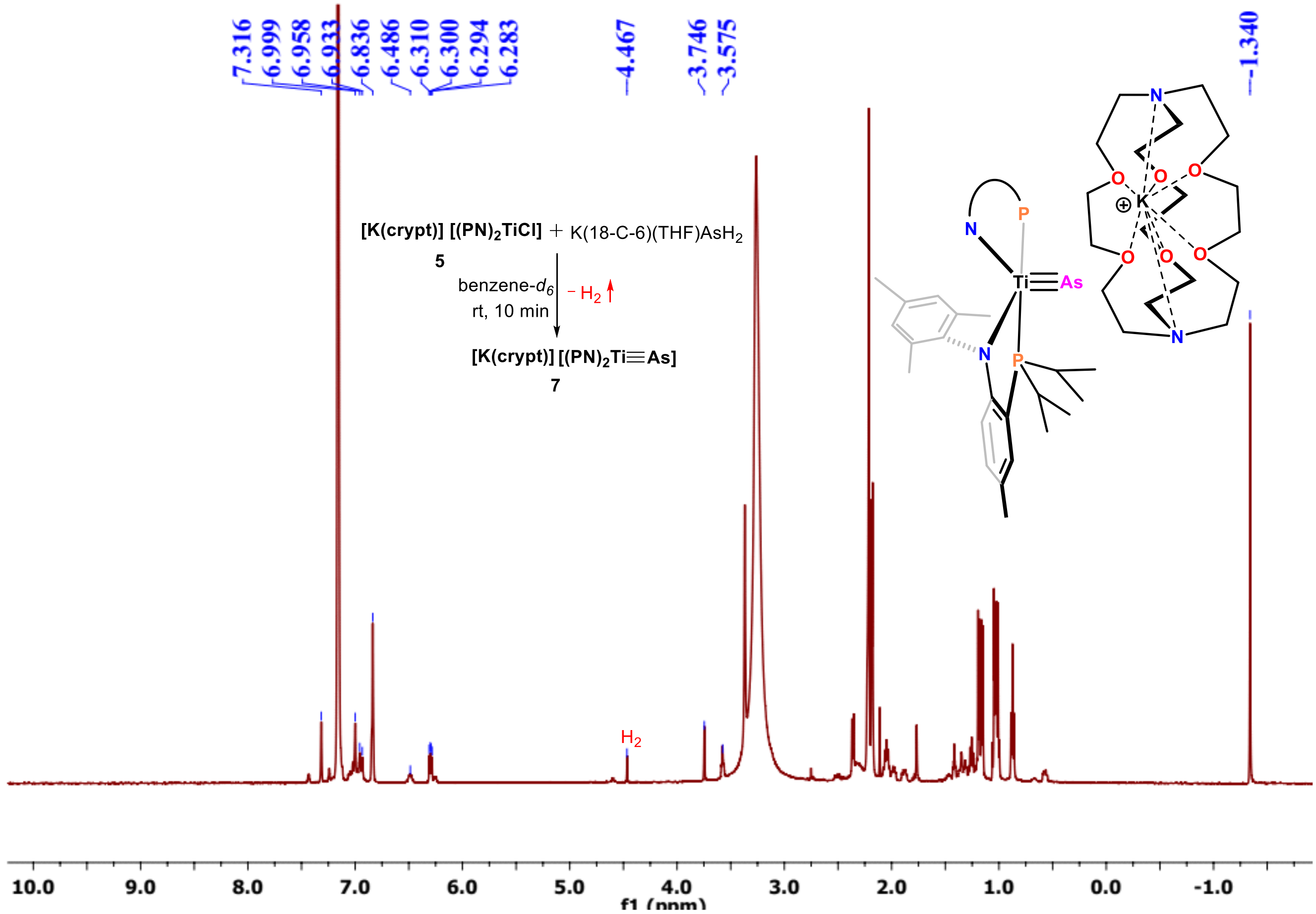

Figure S25: $^1H\{^{31}P\}$ NMR spectrum of **7** in benzene–d_6, 500 MHz, 298 K. The resonance at 4.47 ppm indicating concomitant formation of molecular hydrogen along with the product, **7**.

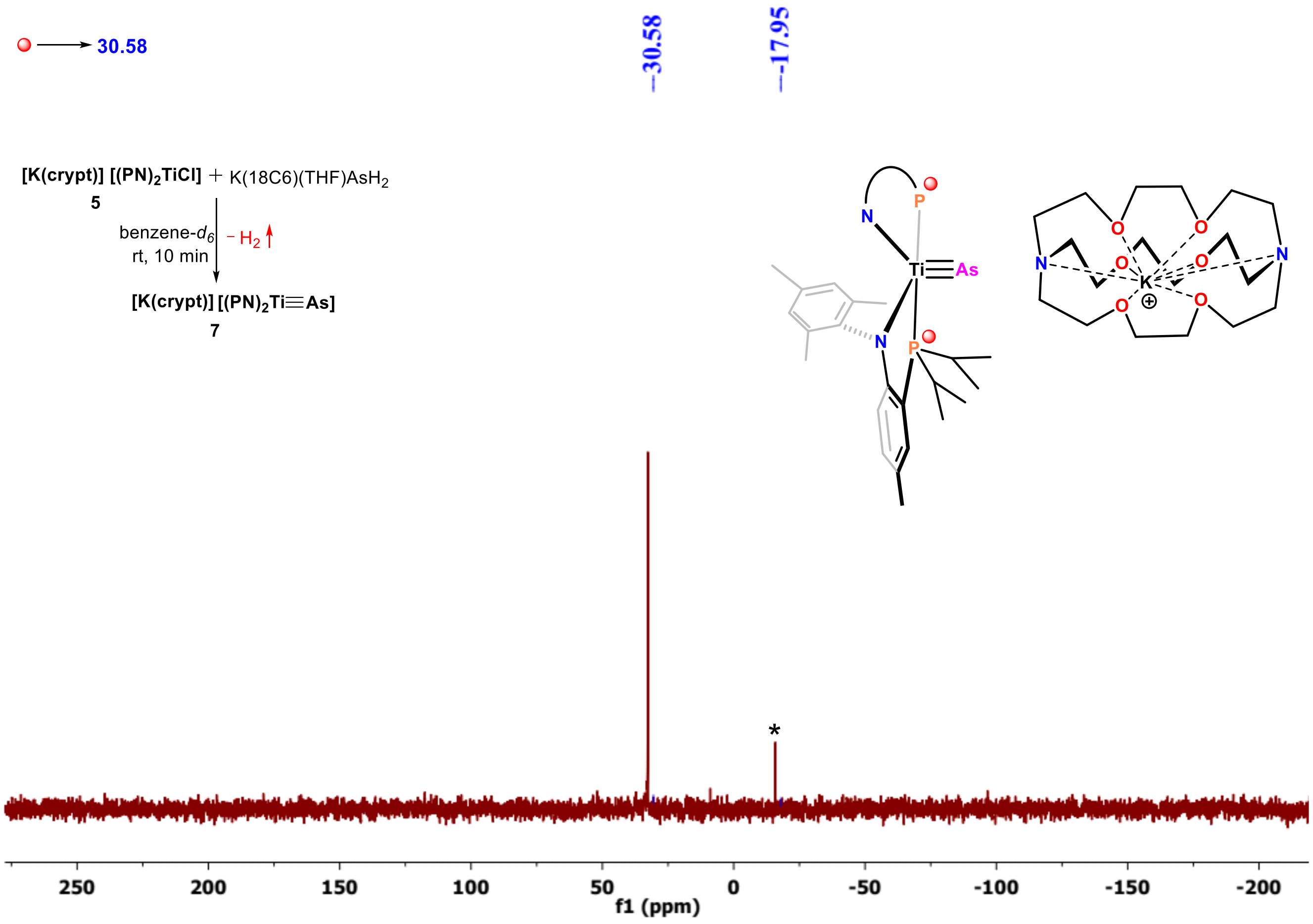

Figure S26: $^{31}P\{^1H\}$ NMR spectrum of **7** in benzene–d_6, 202.5 MHz, 298 K. The asterisks sign corresponds to decomposition product to ligand, HPN.

4.5 NMR spectral data for the reaction mixture of 5 with K(18-C-6)(THF)SbH$_2$

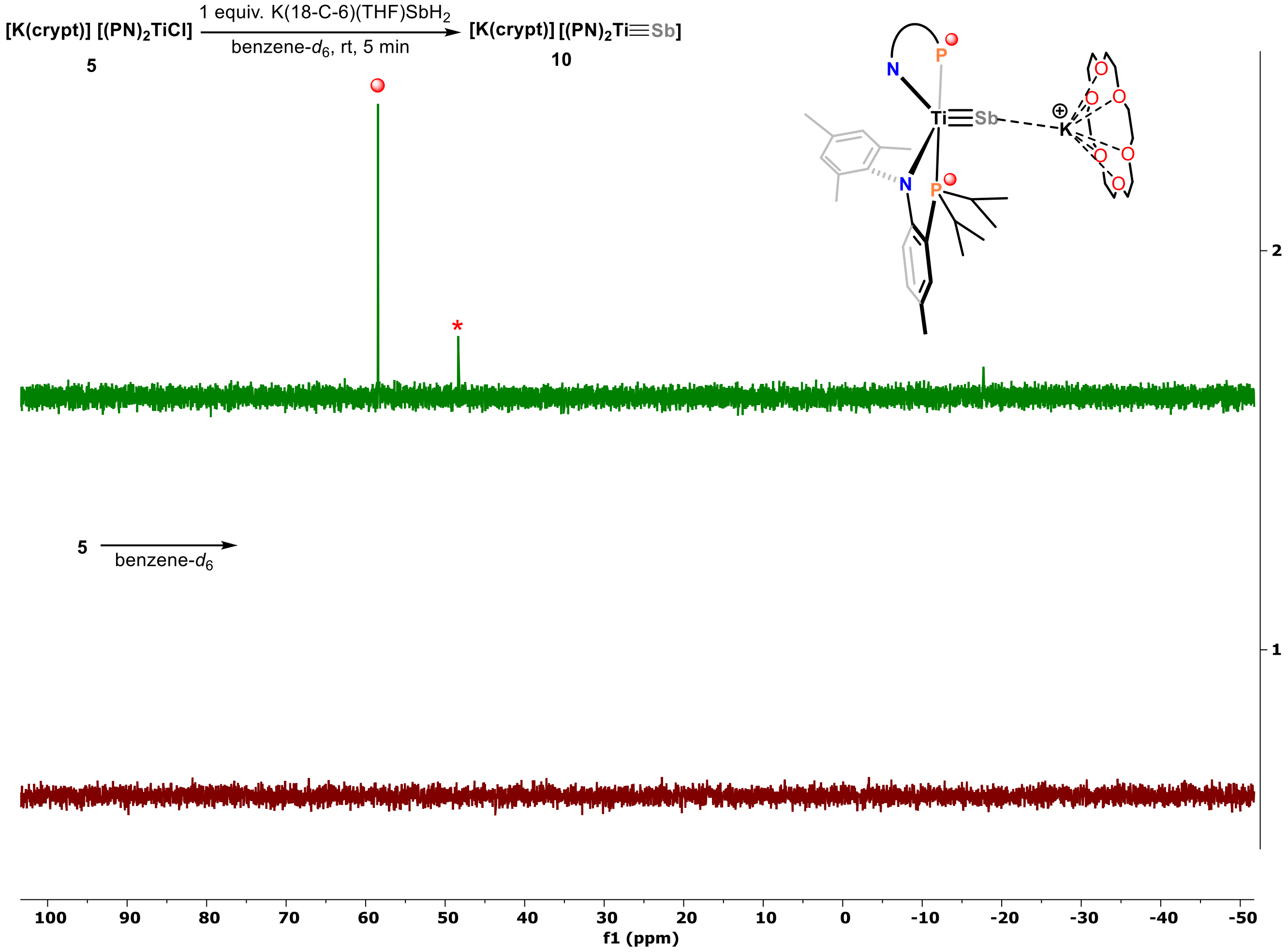

Figure S27: Stacked plot of $^{31}P\{^{1}H\}$ NMR spectroscopy for the reaction mixture of **5** and K(18-C-6)(THF)SbH$_2$ in benzene–d_6, 202.5 MHz, 298 K. The sign * corresponds to minor amount of unknown byproduct. Also, the resonance at -17.70 ppm indicates decomposition product to ligand.

4.6 $^{1}H\{^{31}P\}$ NMR spectroscopy of [K(18-C-6)][$(PN)_2TiCl$] (8)

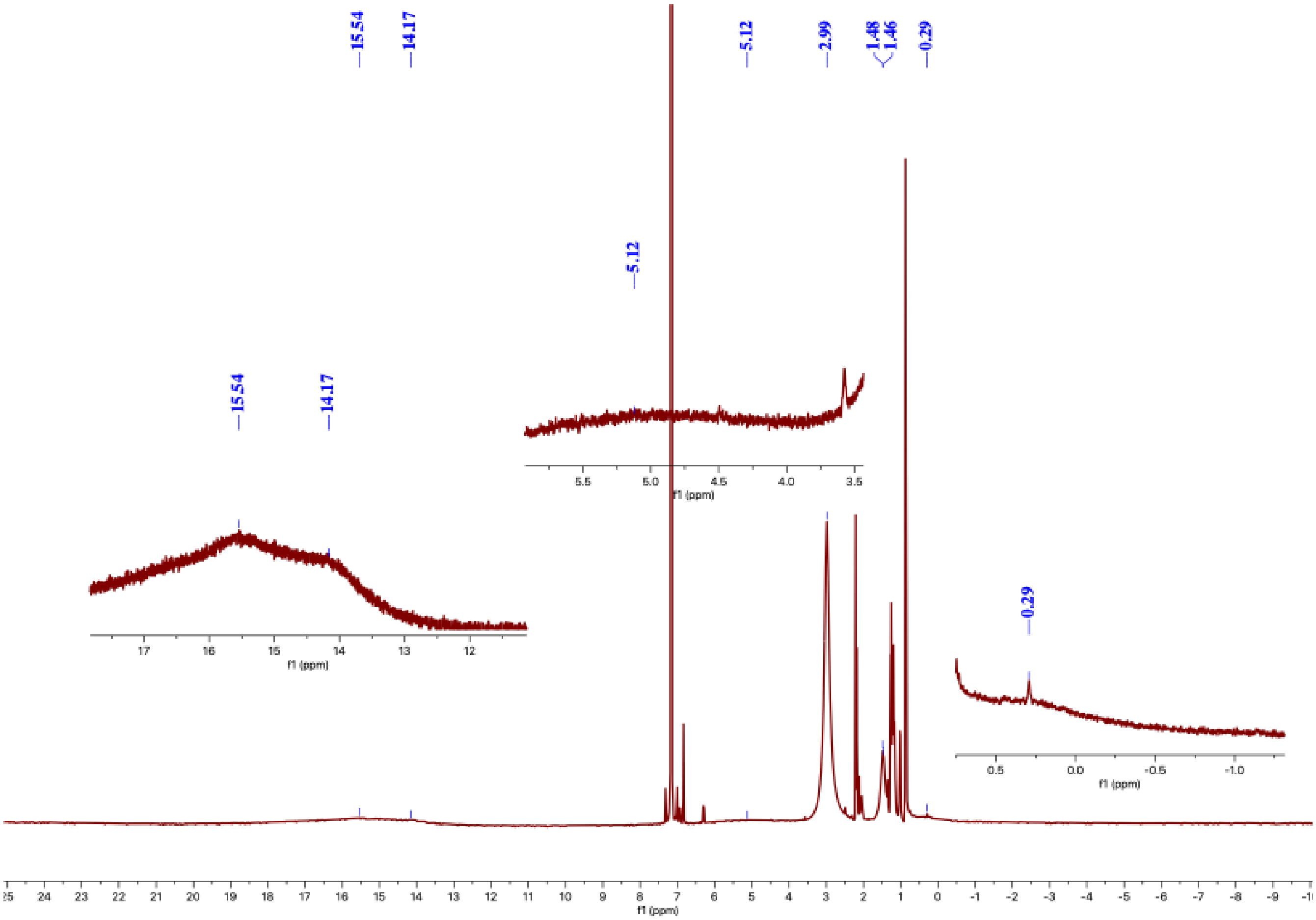

Figure S28: $^{1}H\{^{31}P\}$ NMR spectrum of [K(18-C-6)][$(PN)_2TiCl$] (**8**) in benzene–d_6, 500 MHz, 298 K. Inset: zoom region from 18.0–11.0, 6.5–3.5, and 0.75–(-)1.25 ppm for paramagnetic resonances of compound **8**.

4.7 NMR Spectral Data for [K(18-C-6)][(PN)$_2$Ti≡Sb] (9)

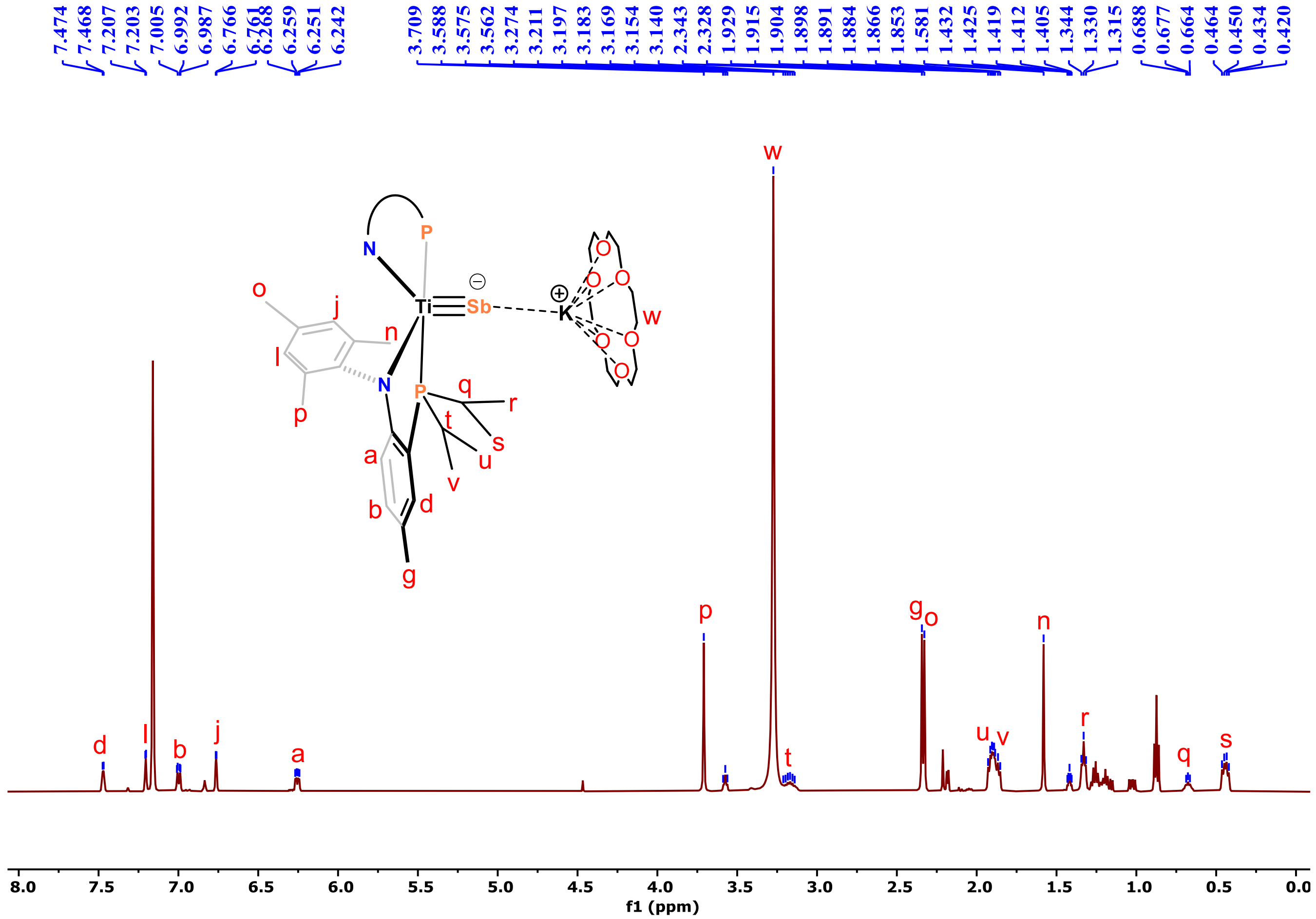

Figure S29: ^{1}H NMR spectrum of **9** in benzene–d_6, 500 MHz, 298 K. The resonances at 1.25, and 0.87 (pentane) ppm and 3.58, and 1.42 (THF) ppm corresponds to residual solvents and 4.47 ppm correspond to molecular dihydrogen.[24] Moreover, the resonances at 7.32, 6.93, 6.84, 6.31–6.28, 3.41, 2.21, 2.19, 2.18, 2.11–2.02, 1.22–1.15, and 1.04–1.01 ppm corresponds to trace amount of unidentified impurities.

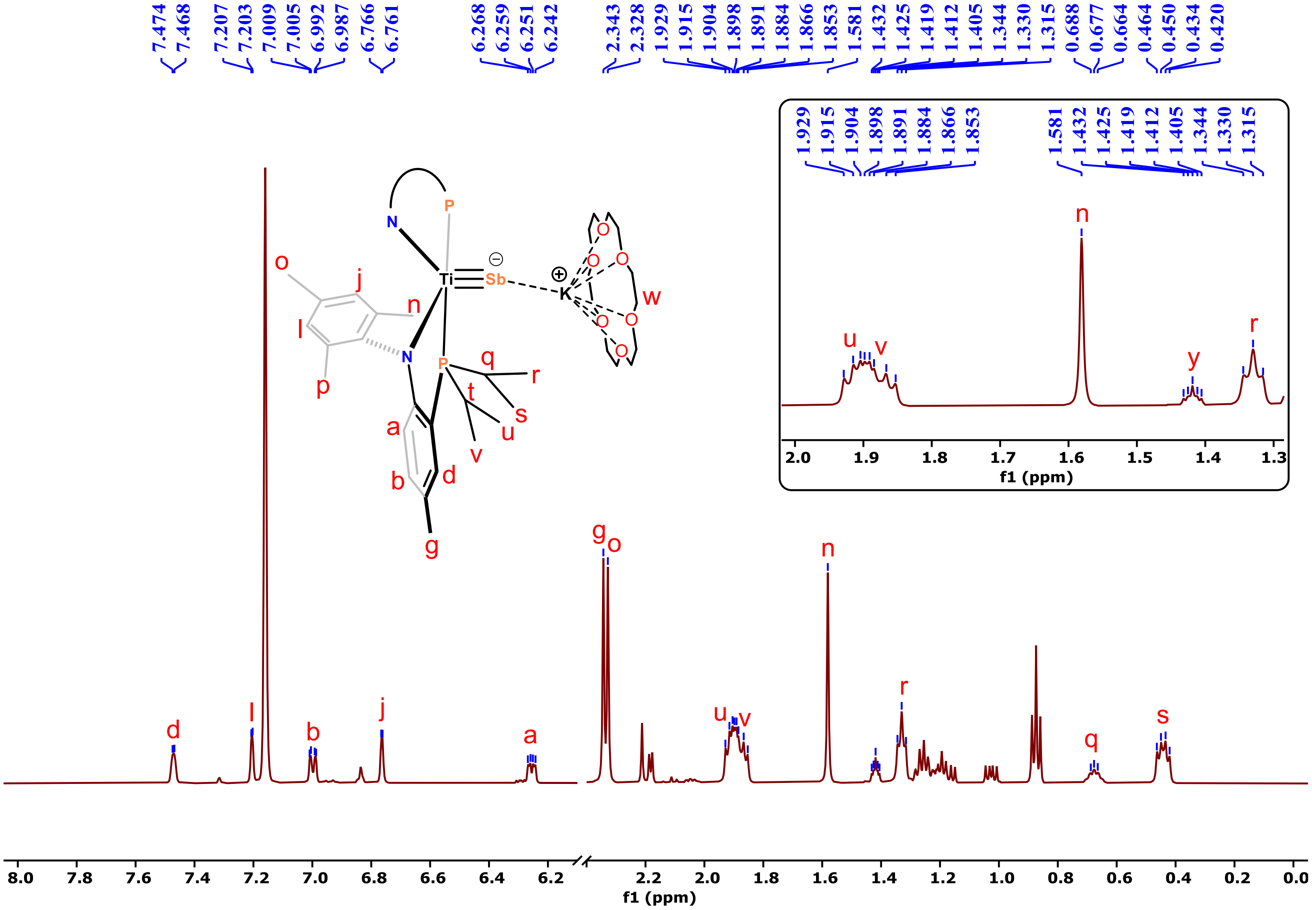

Figure S30: ^{1}H NMR spectrum of **9** in benzene–d_6, 500 MHz, 298 K from region 8.0–6.1 and 2.3–0.0 ppm. Inset: zoom region from 2.0–1.3 ppm for clear assignments of all the salient proton resonances. The resonances at 1.25, and 0.87 ppm corresponds to residual pentane.[24] Moreover, the resonances at 7.32, 6.93, 6.84, 6.31–6.28, 2.21, 2.19, 2.18, 2.11–2.02, 1.22–1.15, and 1.04–1.01 ppm corresponds to trace amount of unidentified impurities.

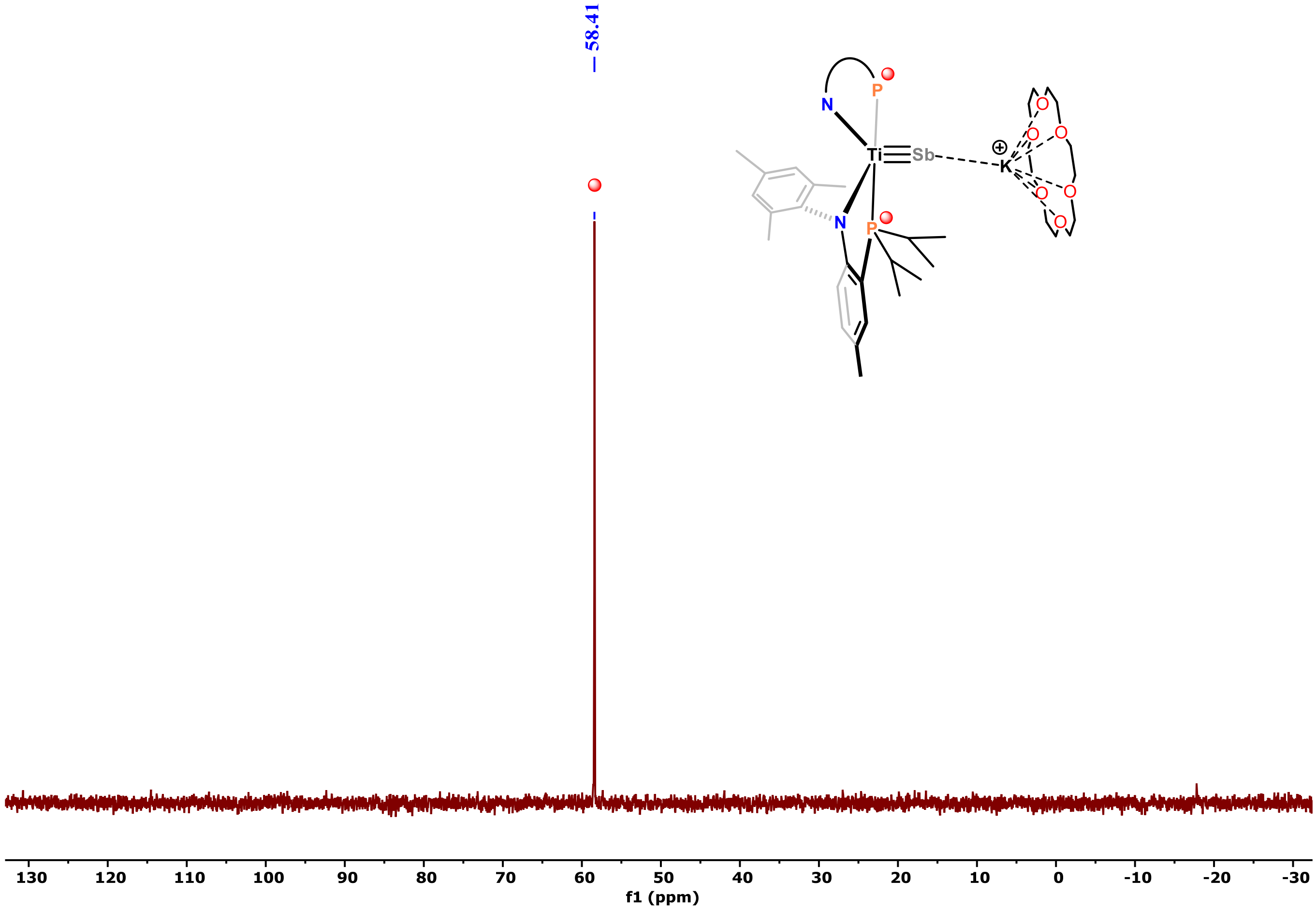

Figure S31: $^{31}P\{^{1}H\}$ NMR spectrum of **9** in benzene–d_6, 202.5 MHz, 298 K. The resonance at -17.79 ppm corresponds to minute amount of decomposition product to ligand.

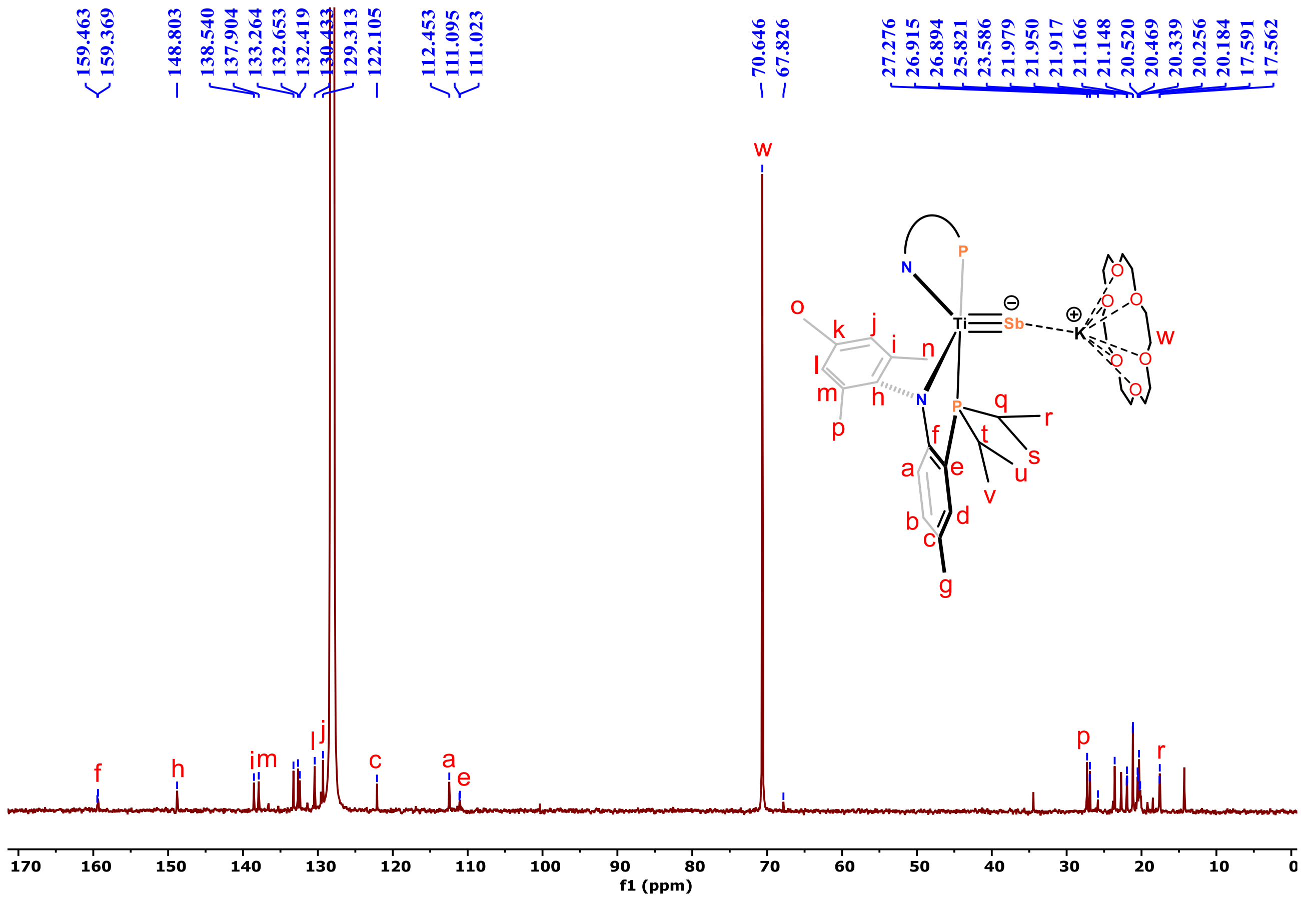

Figure S32: $^{13}C\{^1H\}$ NMR spectrum of **9** in benzene–d_6, 125.8 MHz, 298 K. The resonances at 34.4, 22.7, and 14.3 (pentane) ppm and 67.8, and 25.8 (THF) ppm corresponds to residual solvent molecules.[24] Also, the resonances at 136.6, 135.3, 131.4, 129.6, 100.4, 23.9, 23.8, 20.8, 20.1, 19.3, and 18.5 ppm indicate trace amount of unidentified impurities.

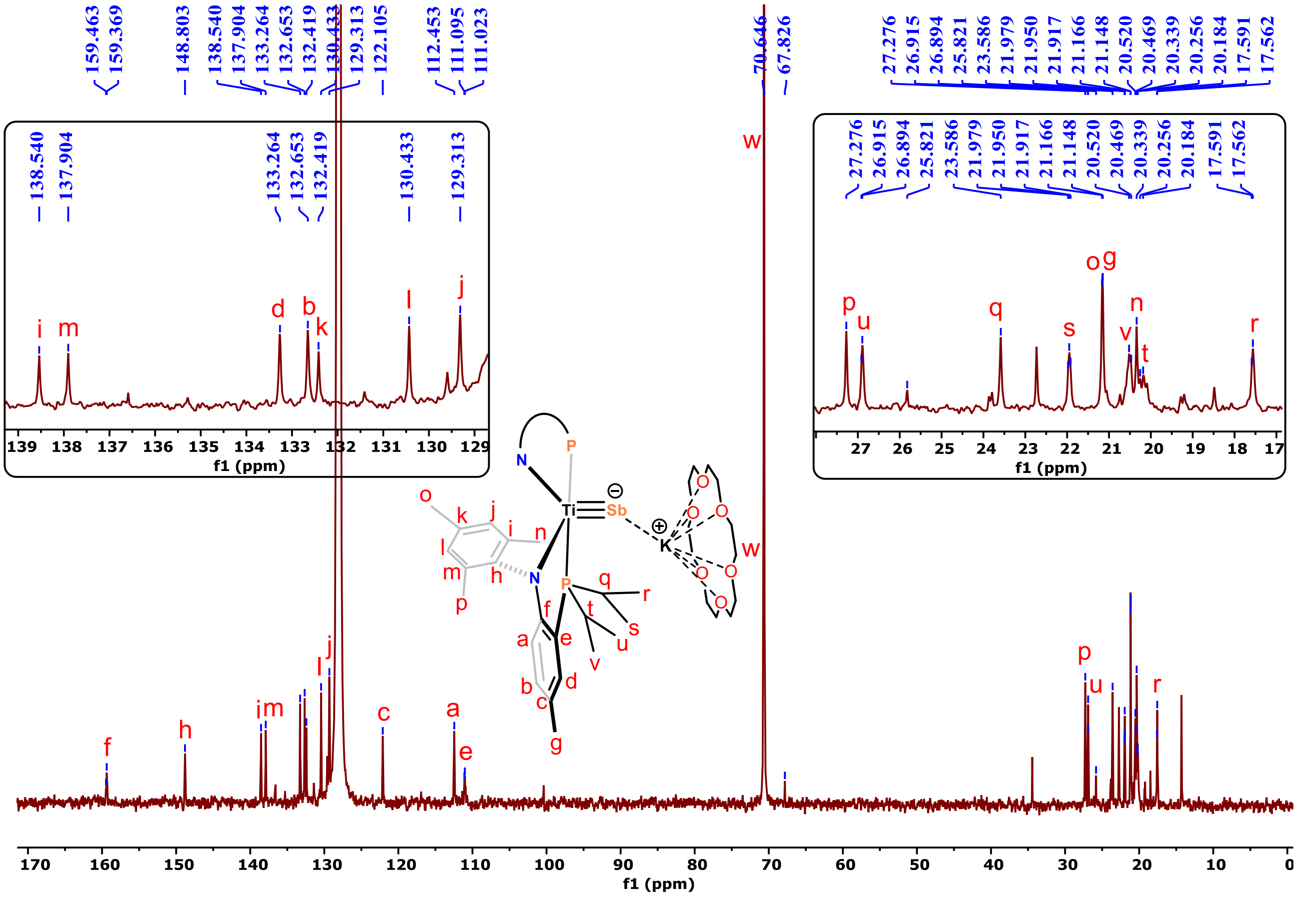

Figure S33: $^{13}C\{^{1}H\}$ NMR spectrum of **9** in benzene–d_6, 125.8 MHz, 298 K. Inset: zoom region from 139.0–129.0 and 28.0–17.0 ppm for clear assignments of all the salient carbon peaks. The resonances at 34.4, 22.7, and 14.3 (pentane) ppm and 67.8, and 25.8 (THF) ppm corresponds to residual solvent molecules.[24] Moreover, the resonances at 136.6, 135.3, 131.4, 129.6, 100.4, 23.9, 23.8, 20.8, 20.1, 19.3, and 18.5 ppm indicate trace amount of unidentified impurities.

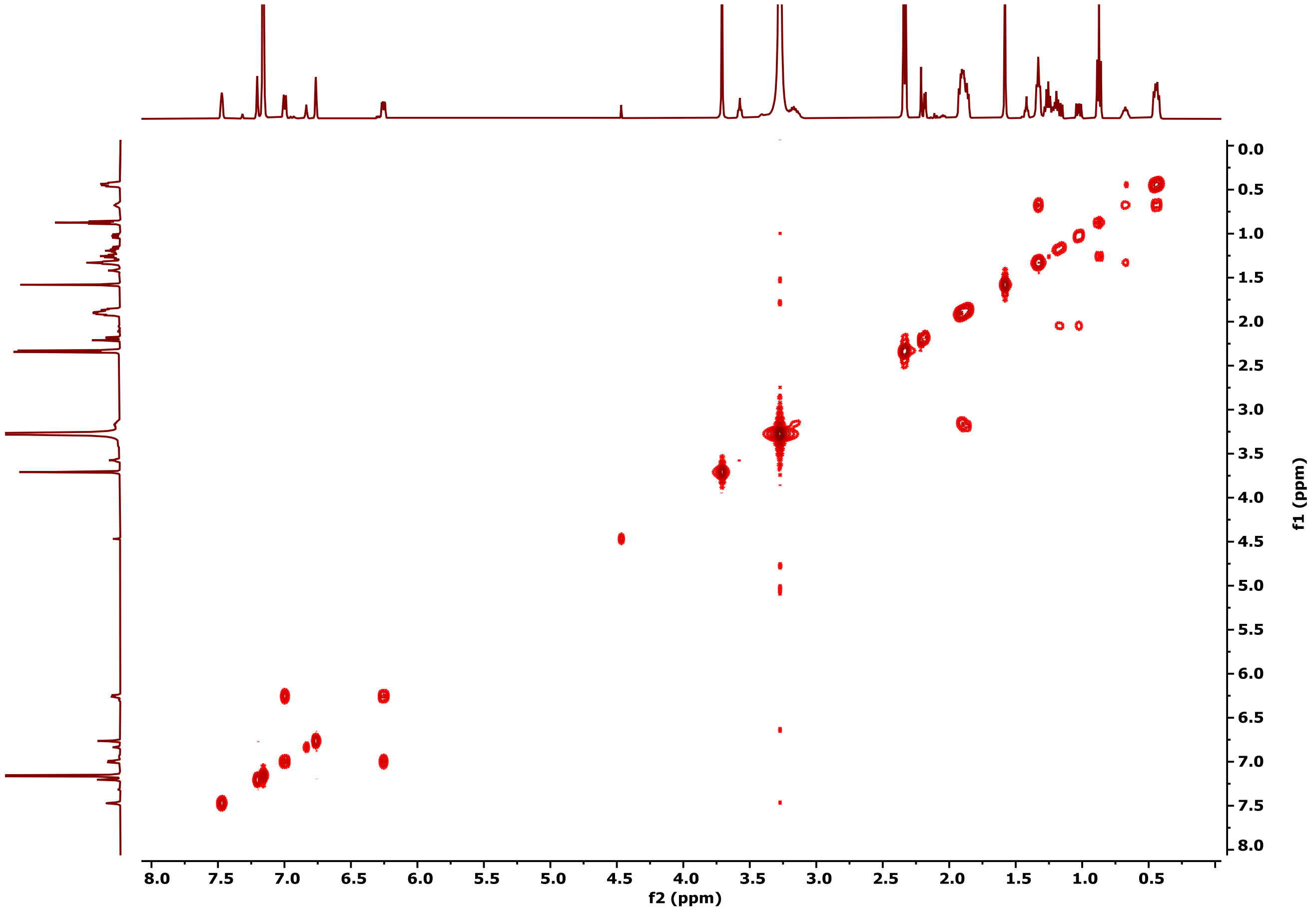

Figure S34: ^{1}H-^{1}H COSY NMR spectrum of **9** in benzene–d_6 (298 K).

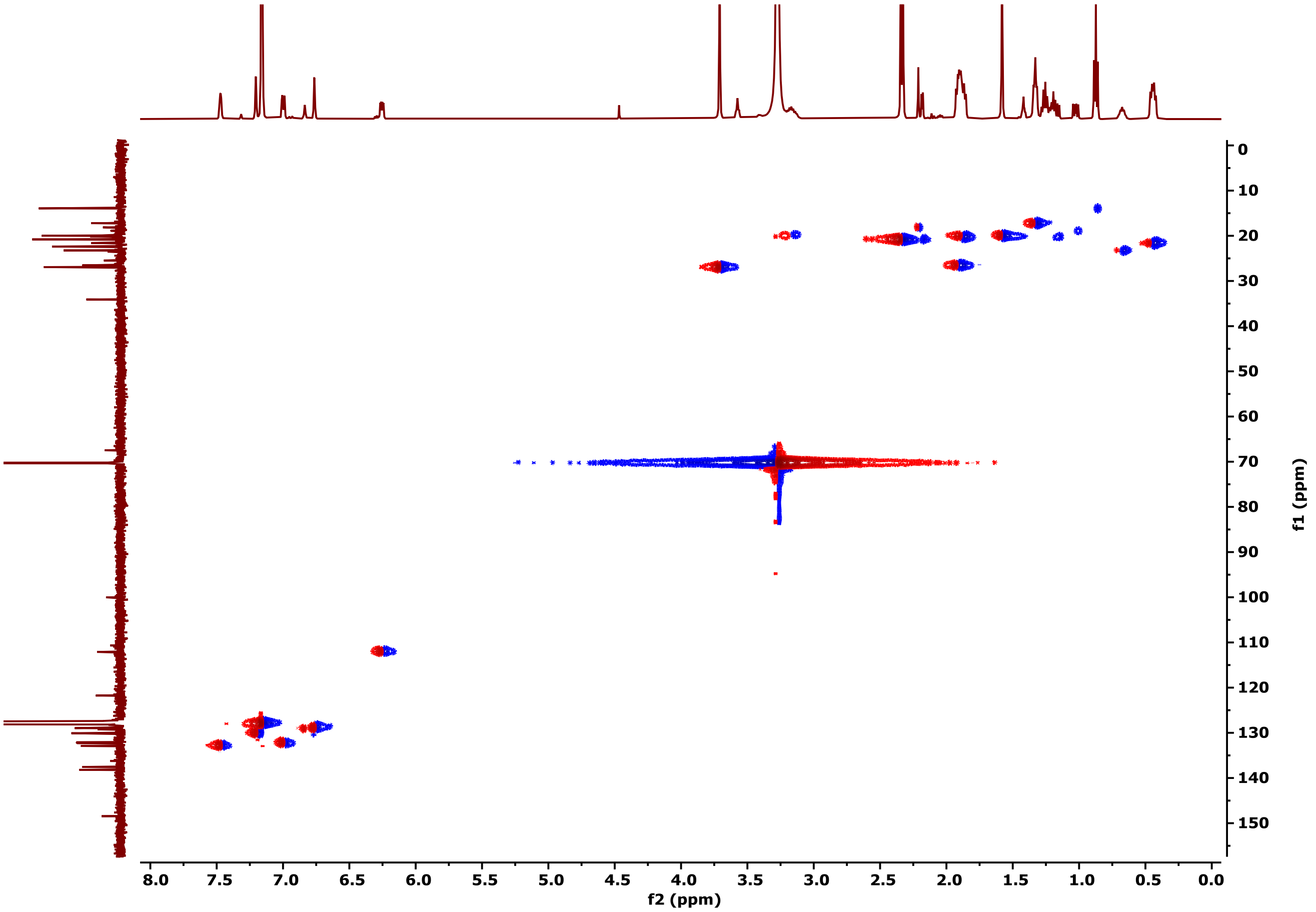

Figure S35: ^{1}H-^{13}C HSQC NMR spectrum of **9** in benzene–d_6 (298 K).

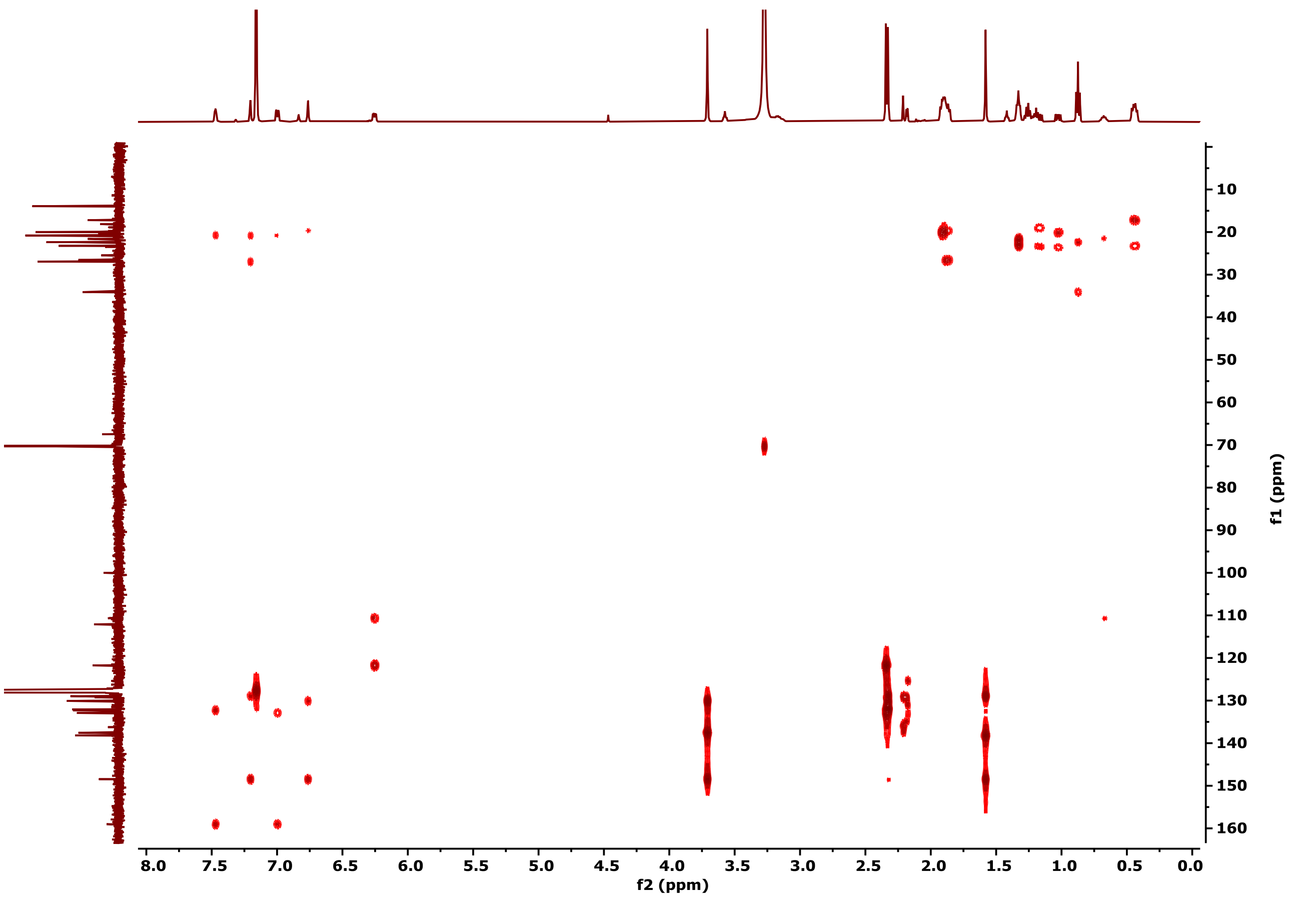

Figure S36: ^{1}H-^{13}C HMBC NMR spectrum of **9** in benzene–d_6 (298 K).

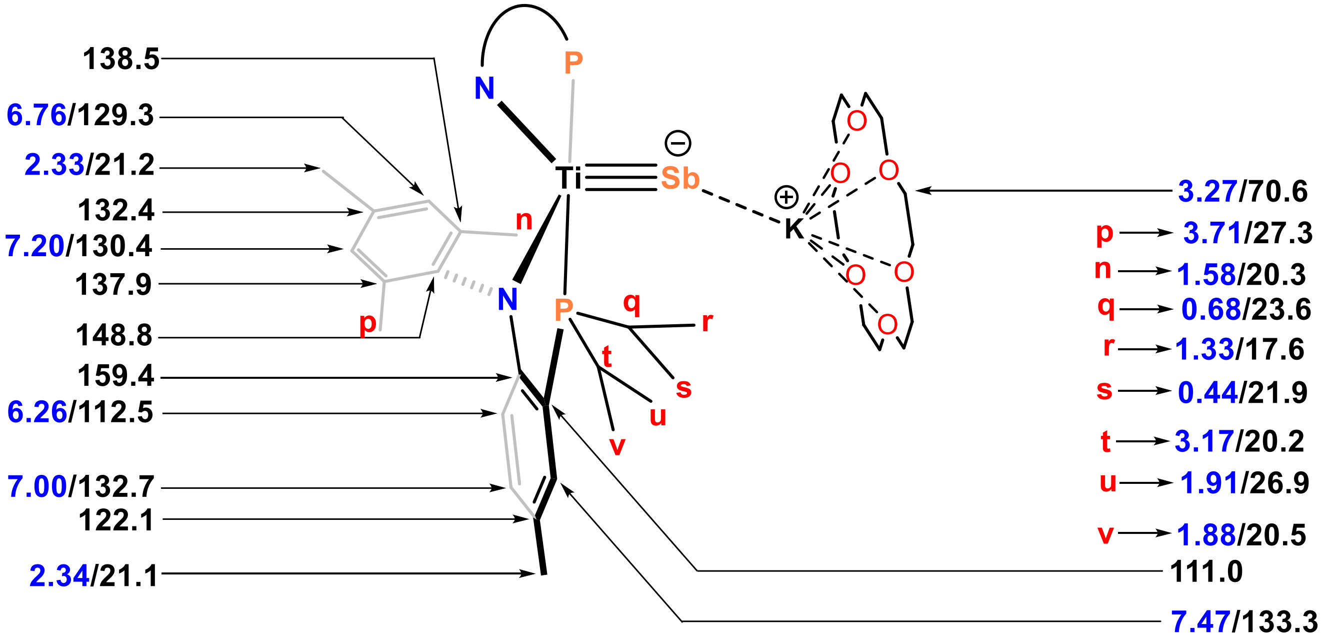

Figure S37: ^{1}H (blue), and ^{13}C{^{1}H} (black) NMR spectral assignments for **9**.

4.8 NMR Spectral Data for [K(crypt)][(PN)$_2$Ti≡Sb] (10)

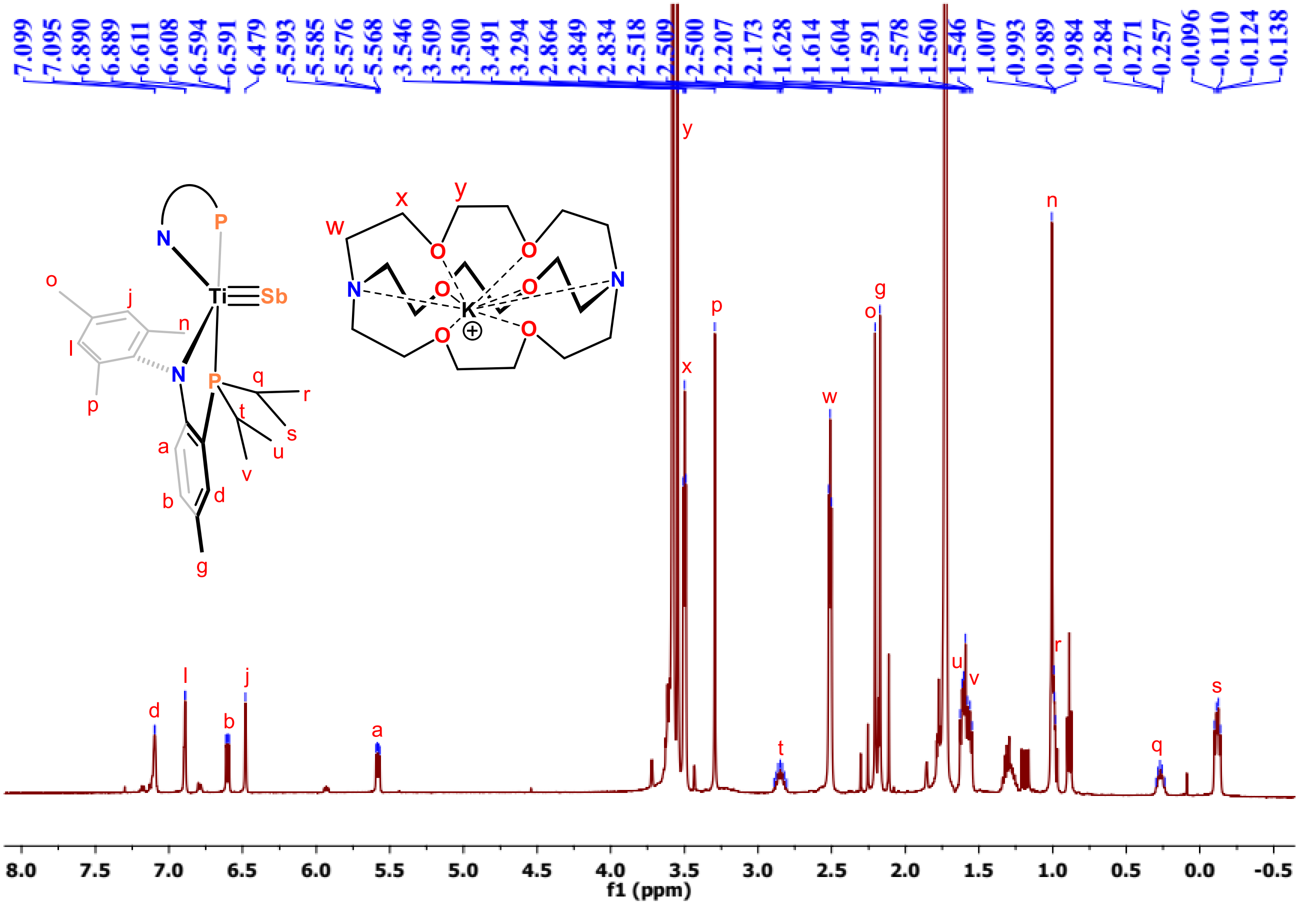

Figure S38: ^{1}H NMR spectrum of **10** in THF–d_8, 500 MHz, 298 K. The resonances at 1.31, and 0.89 (pentane) ppm and 7.19, 7.12, and 2.31 (toluene) ppm corresponds to residual solvents and 4.51 ppm correspond to molecular dihydrogen.[24] Moreover, the resonances at 6.80, 5.93, 3.72, 3.62, 3.43, 2.26, 2.19, 2.11, 1.86, 1.78, 1.34–1.26, and 1.21–1.17 ppm correspond to trace amount of unidentified impurities.

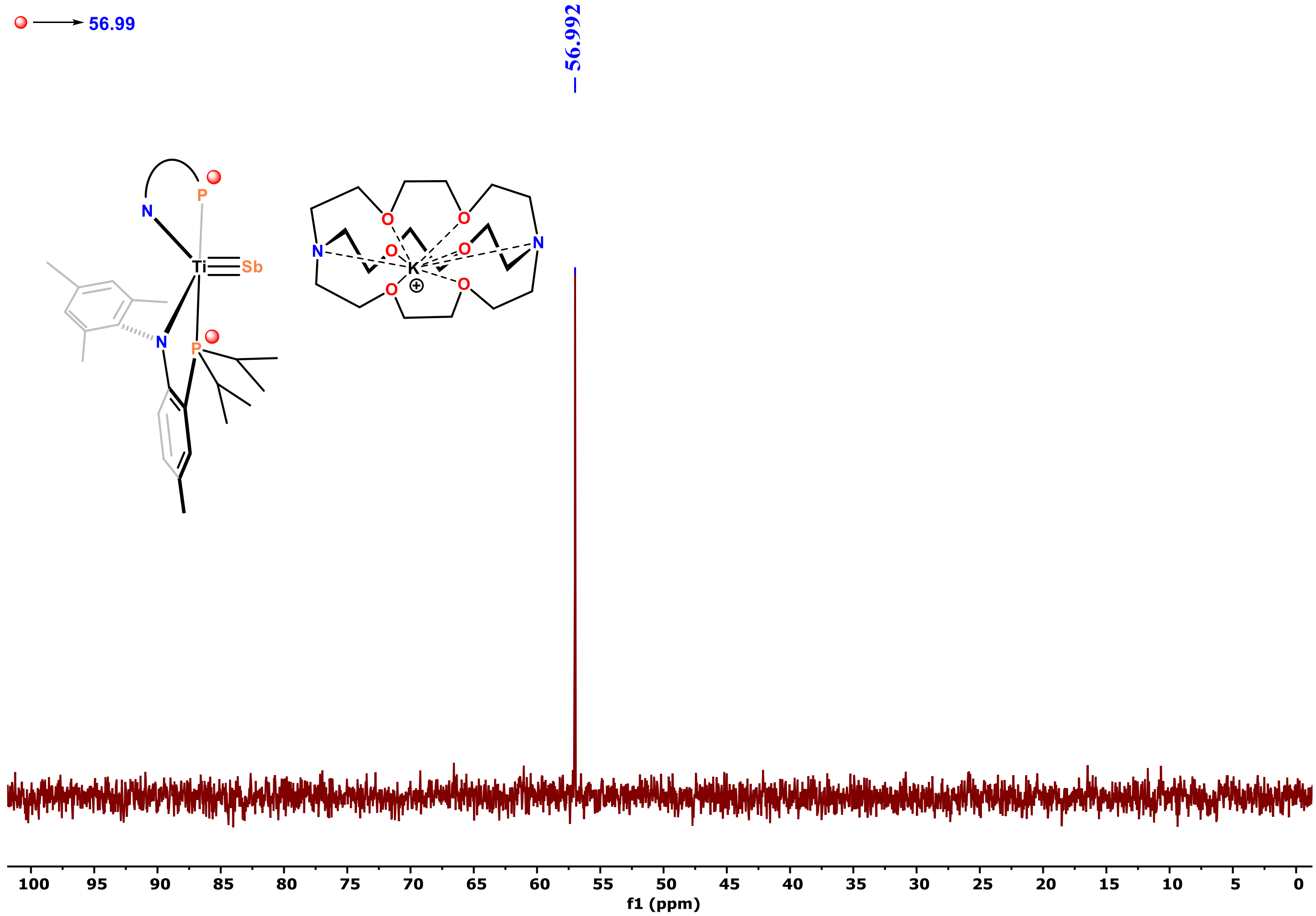

Figure S39: $^{31}P\{^{1}H\}$ NMR spectrum of **10** in THF–d_8, 202.5 MHz, 298 K.

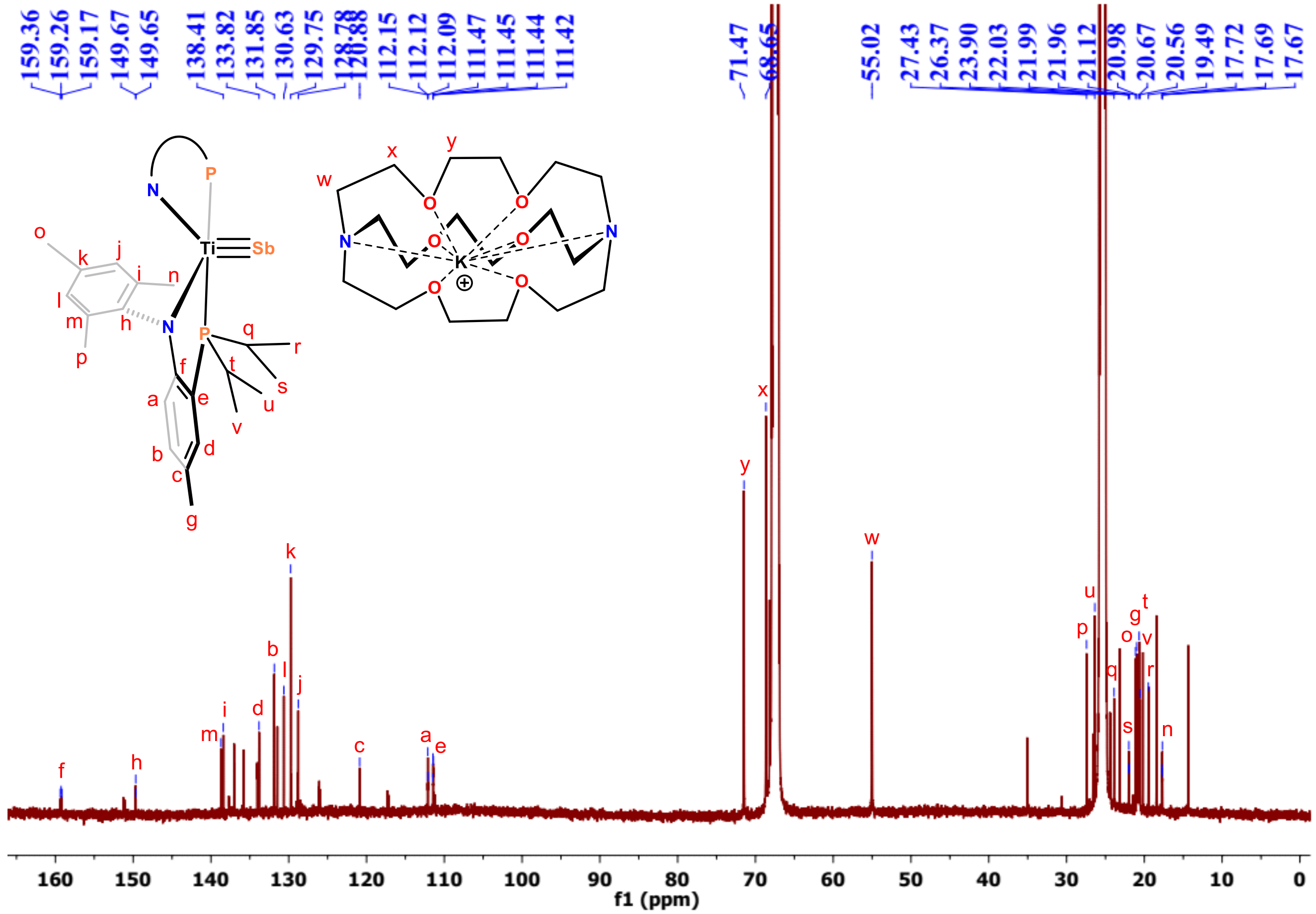

Figure S40: $^{13}C\{^1H\}$ NMR spectrum of **10** in THF–d_8, 125.8 MHz, 298 K. The resonances at 35.1, 23.2, and 14.3 (pentane) ppm and 138.3, 129.6, 128.9, 126.0, and 21.0 (toluene) ppm correspond to residual solvent molecules.[24] The resonances at 151.2, 151.1, 137.7, 137.6, 137.0, 135.8, 134.0, 131.5, 126.1, 117.3, 111.2, 26.6, 24.4, 20.8, 20.2, 19.4, and 18.5 ppm indicate trace amount of unidentified impurities.

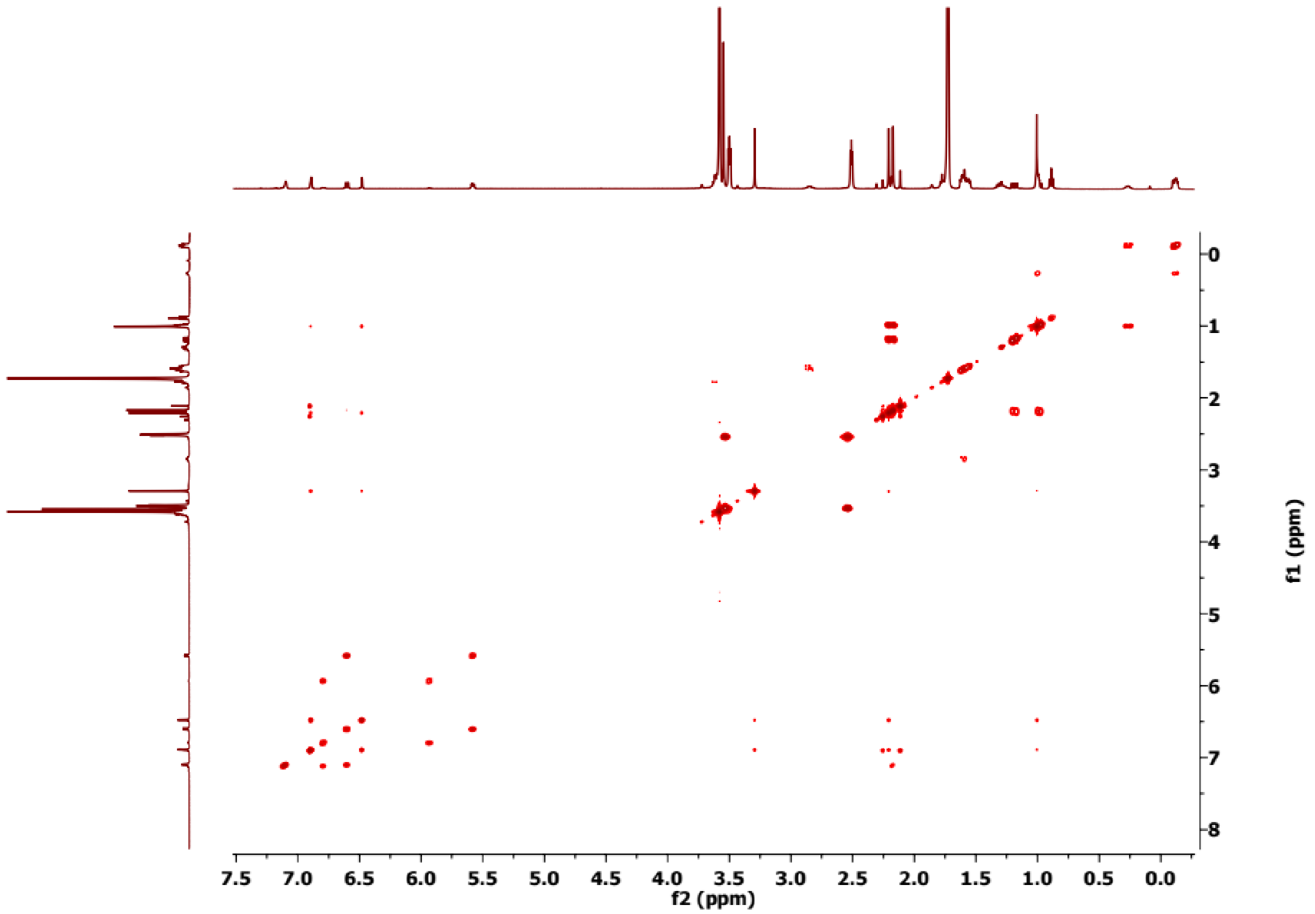

Figure S41: ^{1}H-^{1}H COSY NMR spectrum of **10** in THF–d_8 (298 K).

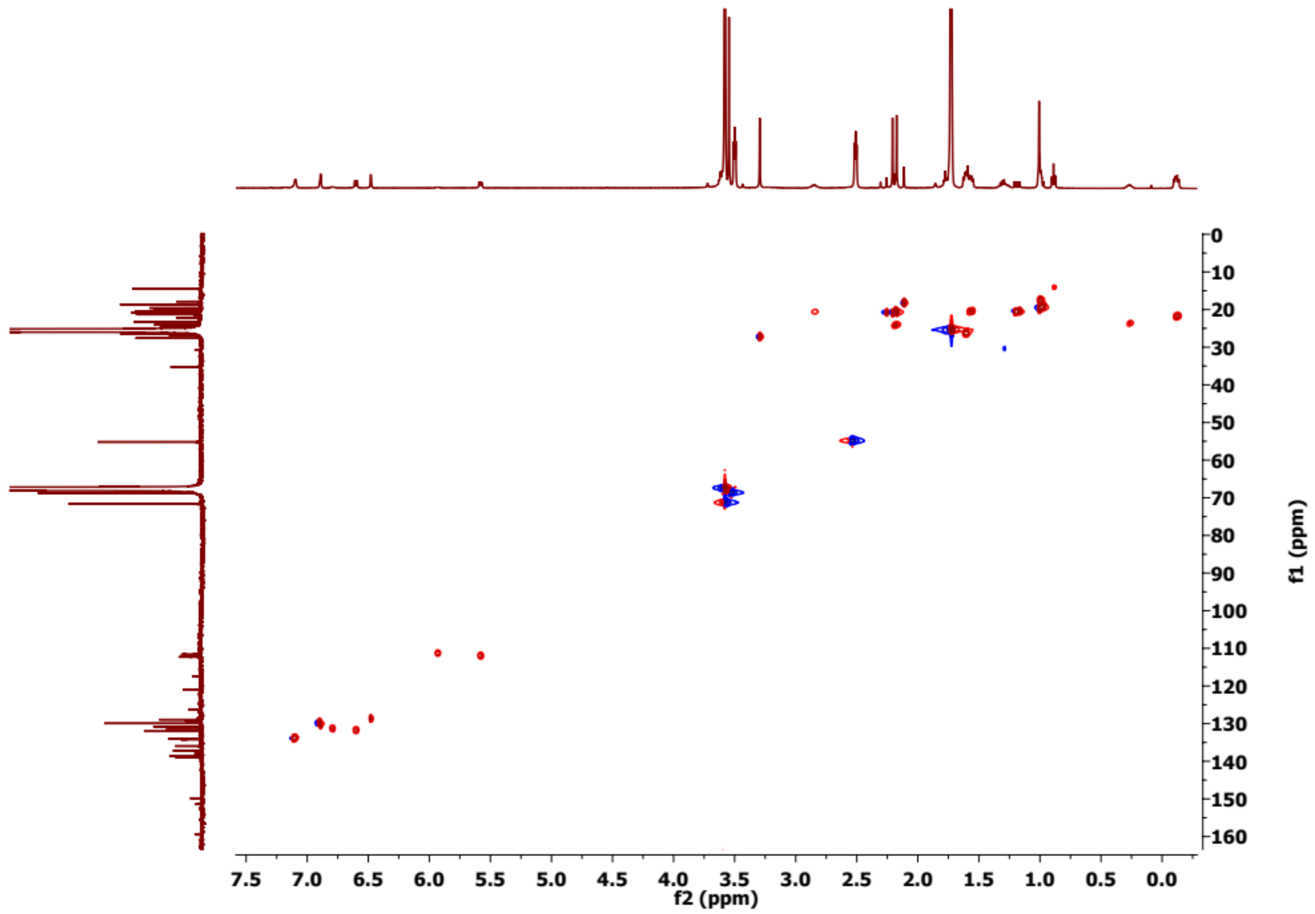

Figure S42: ^{1}H-^{13}C HSQC NMR spectrum of **10** in THF–d_8 (298 K).

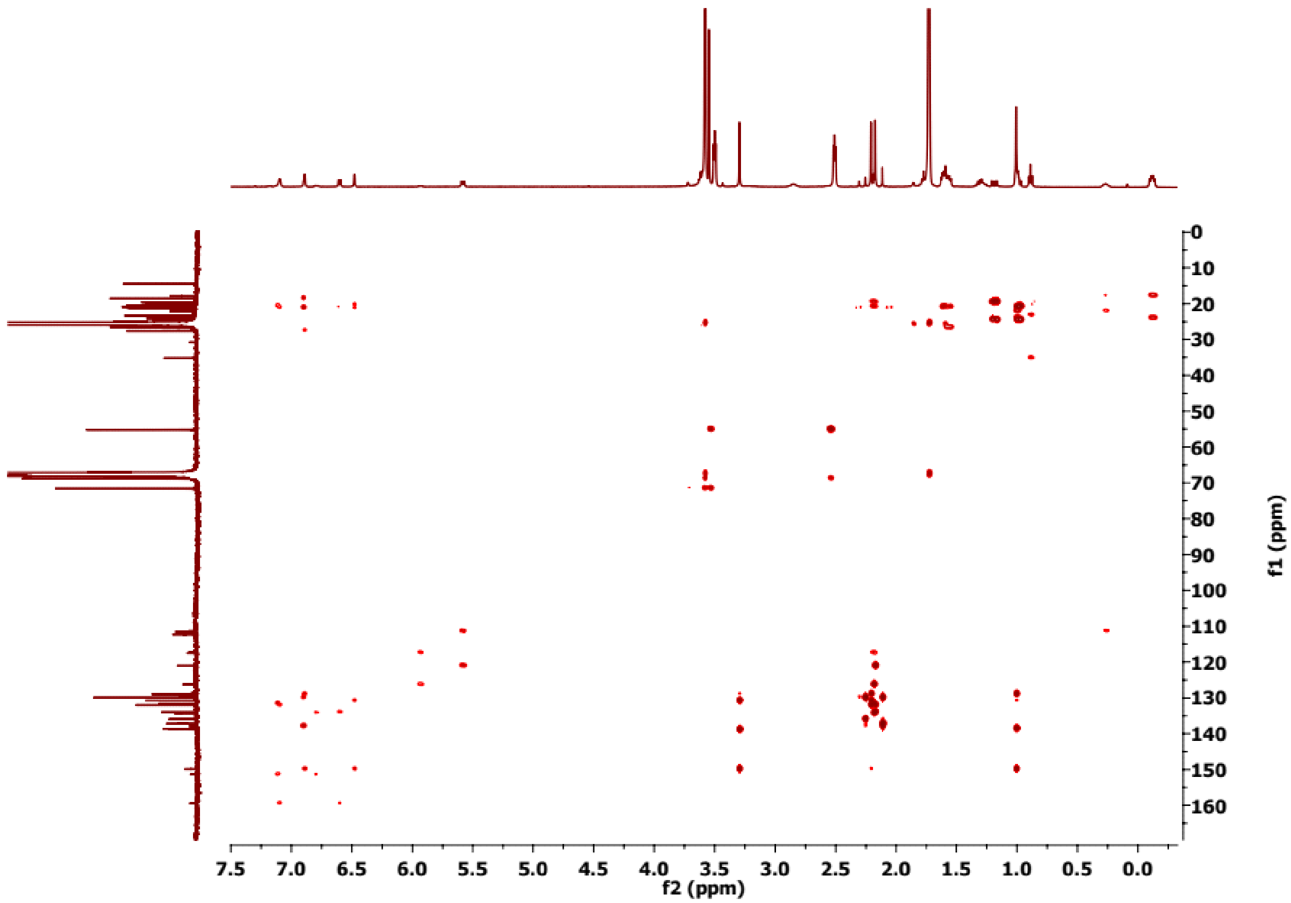

Figure S43: ^{1}H-^{13}C HMBC NMR spectrum of **10** in THF–d_8 (298 K).

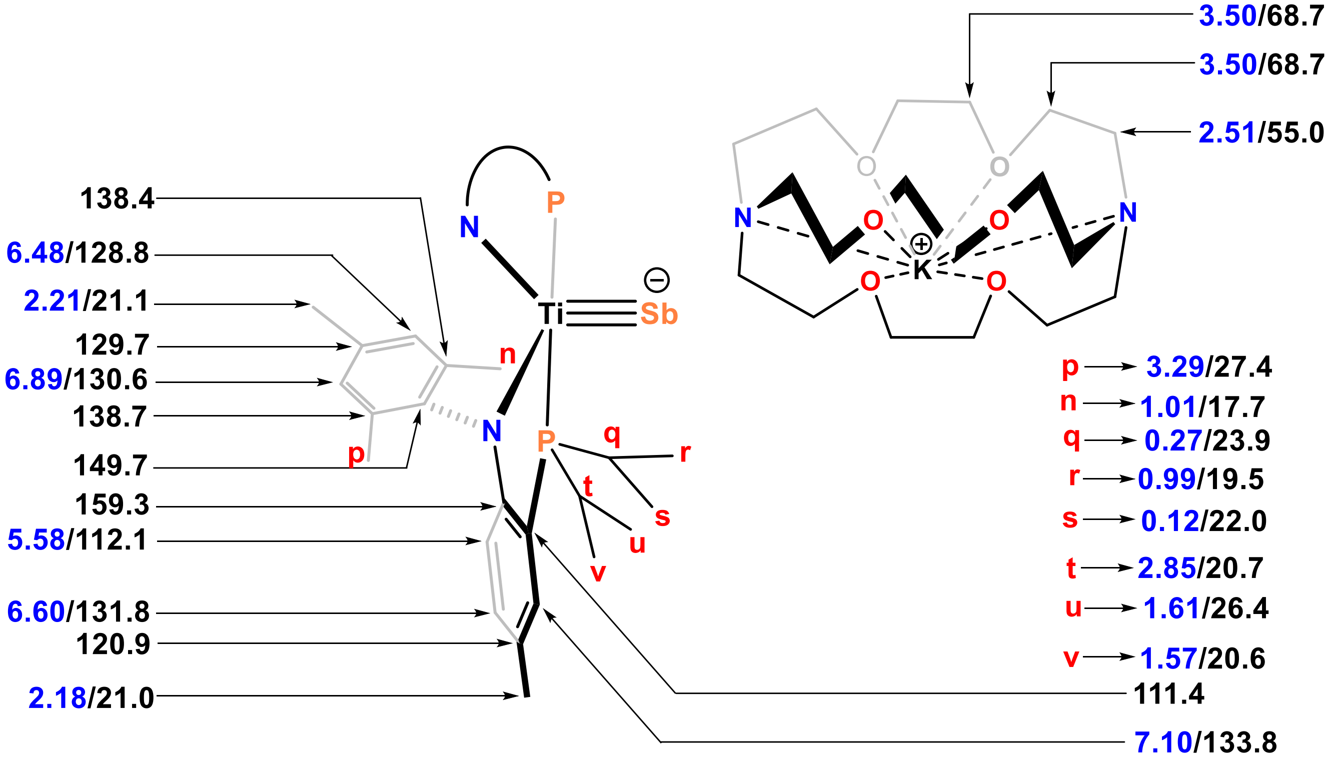

Figure S44: ^{1}H (blue), and $^{13}C\{^{1}H\}$ (black) NMR spectral assignments for **10**.

4.9 NMR spectral data for the reaction of [(PN)(PN')Zr(H)] (1) with $NaNH_2$ and 18-C-6

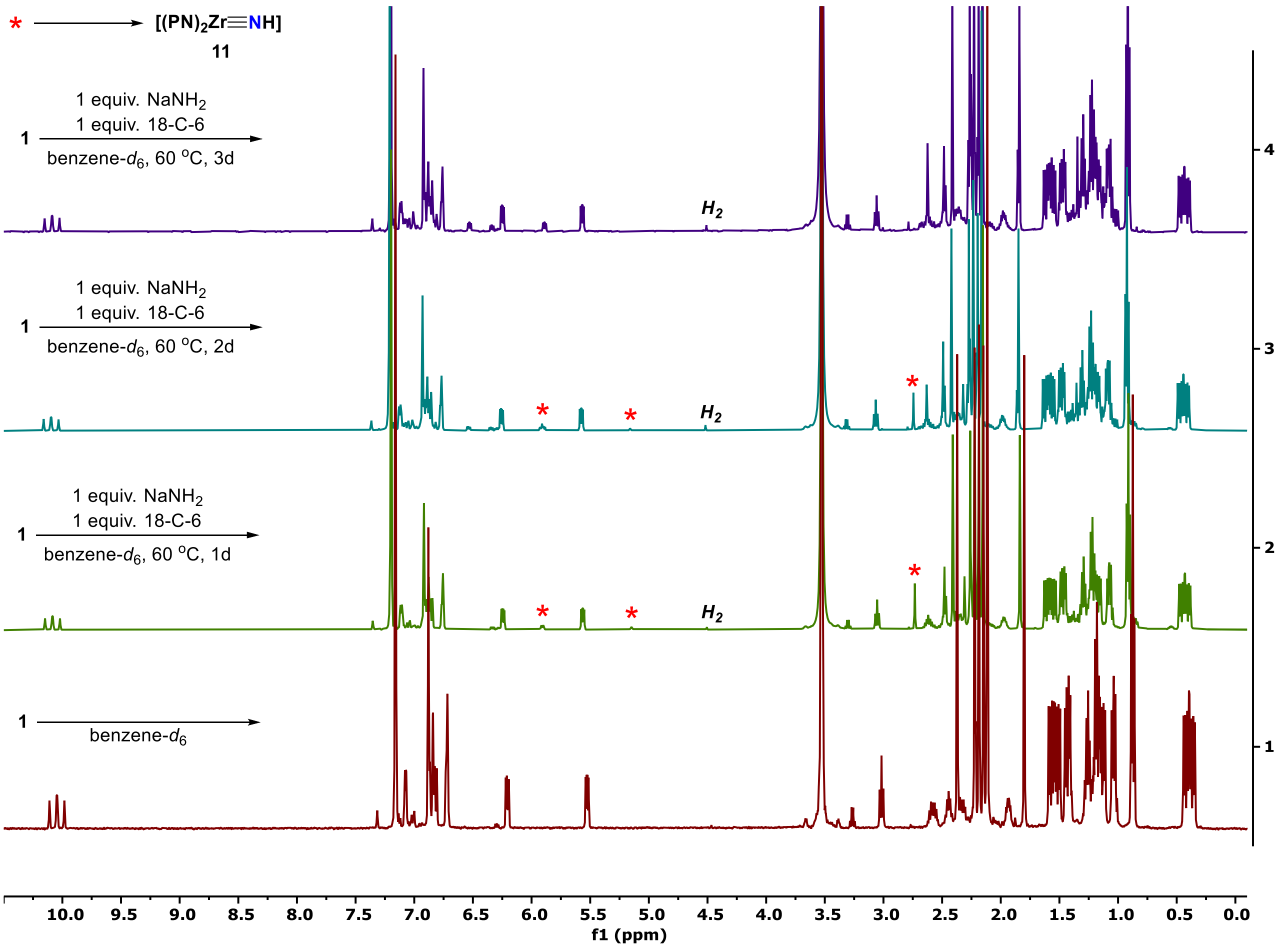

Figure S45: Stacked plot of $^{1}H\{^{31}P\}$ NMR spectrum in benzene-d_6 (500 MHz, 298 K) for the reaction mixture of [(PN)(PN')Zr(H)] (**1**) with 1 equiv. $NaNH_2$ and 1 equiv. 18-C-6 at 60 °C after 1d, 2d, and 3d respectively. The sign * indicates the formation of parent zirconium imide (**11**) with different time.

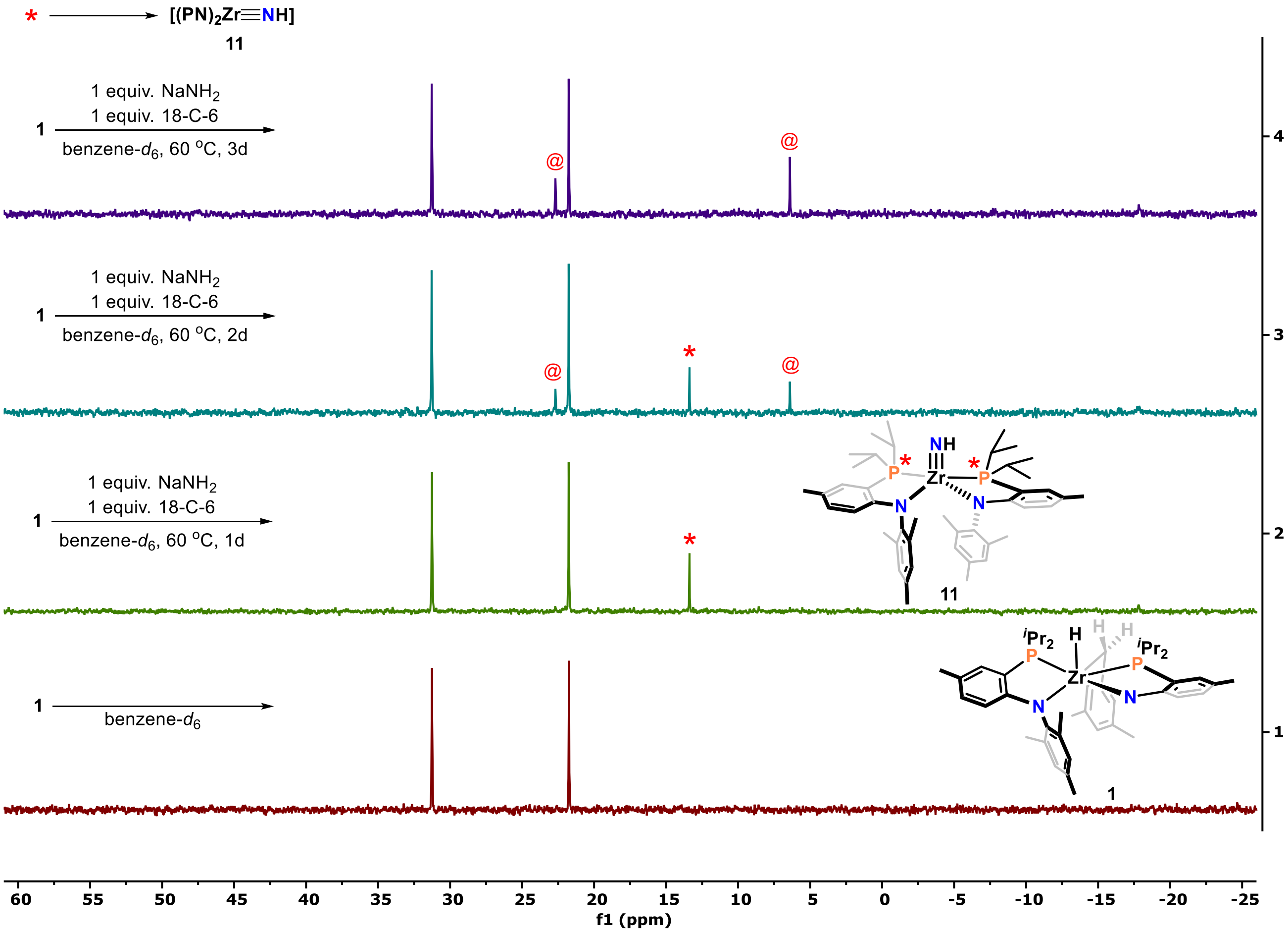

Figure S46: Stacked plot of $^{31}P\{^{1}H\}$ NMR spectrum in benzene-*d₆* (202.5 MHz, 298 K) for the reaction mixture of [(PN)(PN')Zr(H)] (**1**) with 1 equiv. $NaNH_2$ and 1 equiv. 18-C-6 at 60 °C after 1d, 2d, and 3d respectively. The sign * indicates the formation of parent zirconium imide (**11**) with Zr≡*N*H moiety at different time interval. The sign @ denotes appearance of unknown byproduct.

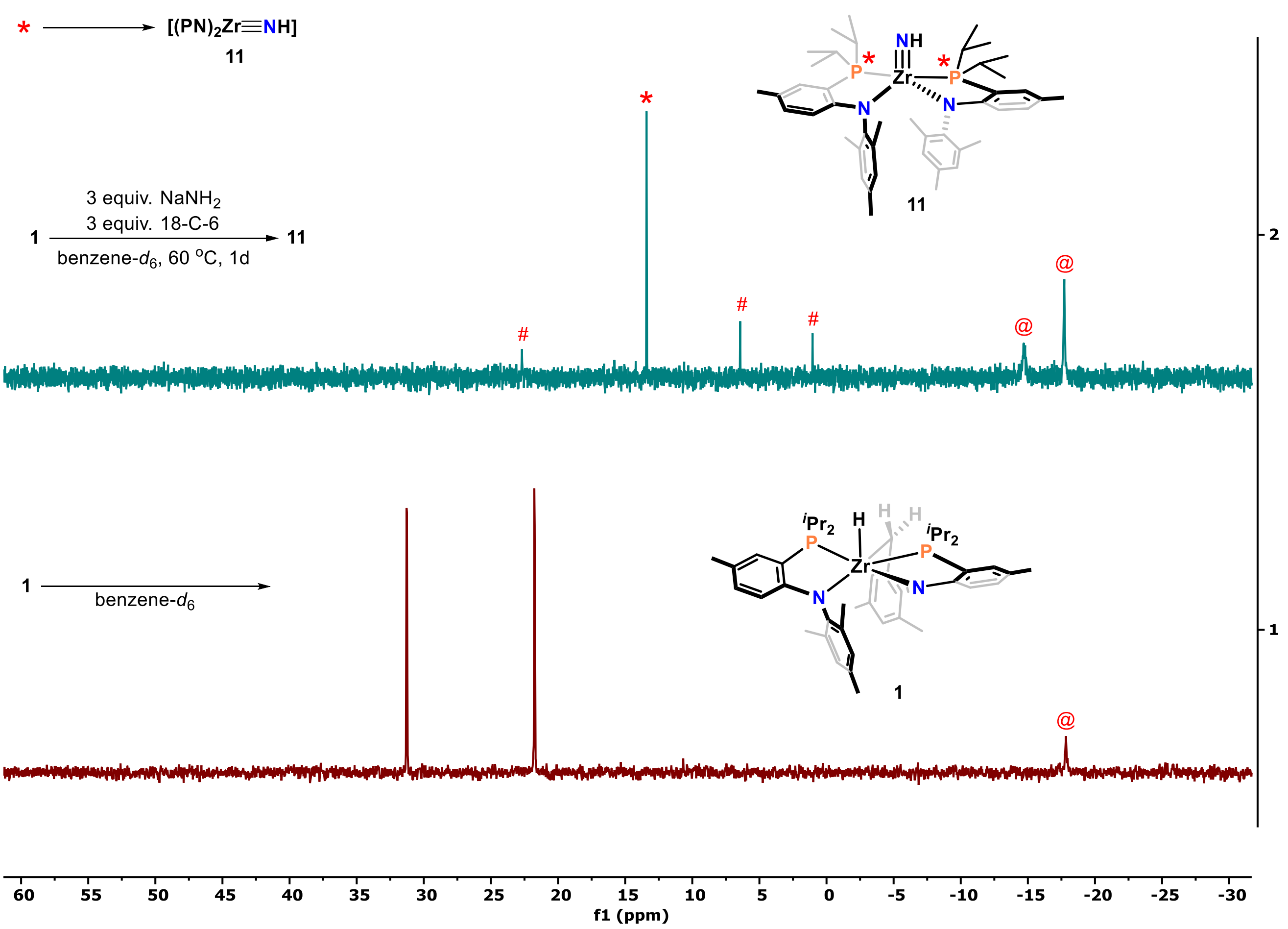

Figure S47: Stacked plot of $^{31}P\{^{1}H\}$ NMR spectrum in benzene-d_6 (202.5 MHz, 298 K) for the reaction mixture of [(PN)(PN')Zr(H)] (**1**) with 3 equiv. $NaNH_2$ and 3 equiv. 18-C-6 at 60 °C after 1d. The sign * denotes the formation of parent zirconium imide (**11**) with Zr≡*N*H functional group, whereas, the sign # denotes formation of minute amount of unknown byproduct. Also, the sign @ denotes appearance of corresponding PN-H (-17.75 ppm)[1] and PN-Na (-15.1 ppm).

5. UV-*vis* Spectroscopy

5.1 UV-*vis* Spectral Data for [K(18-C-6)(THF)$_2$][(PN)$_2$Zr≡Sb] (2) in THF

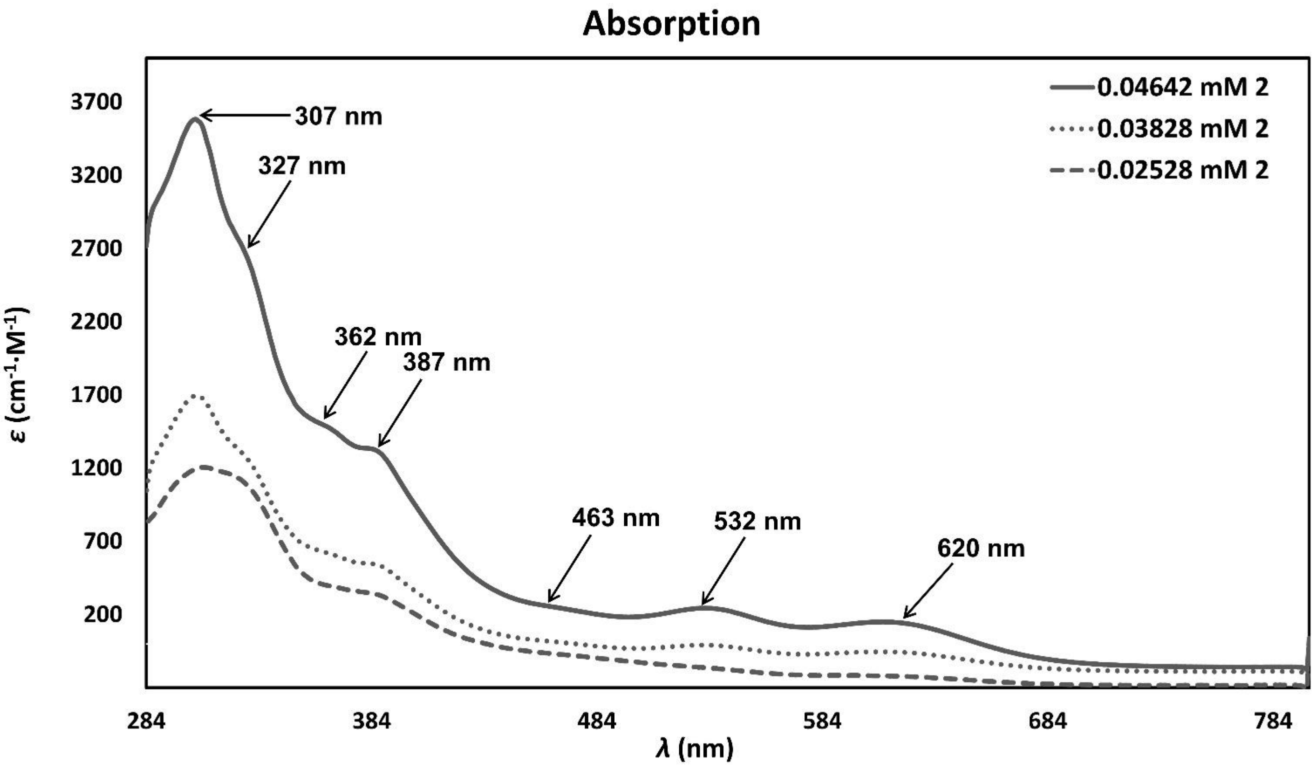

Figure S48: UV-*vis* spectroscopy of [K(18-C-6)(THF)$_2$][(PN)$_2$Zr≡Sb] (**2**) (grey) in THF in the region of 284 to 800 nm. UV-*vis* spectrum of **2** was collected at different concentrations in THF such as 4.64x10^{-5} M (Grey, solid), 2.53x10^{-5} M (Grey, solid-dot), and 3.83x10^{-5} M (Grey, dotted).

5.2 UV-*vis* Spectral Data for [K(18-C-6)(THF)$_2$][(PN)$_2$Zr≡As] (3) in THF

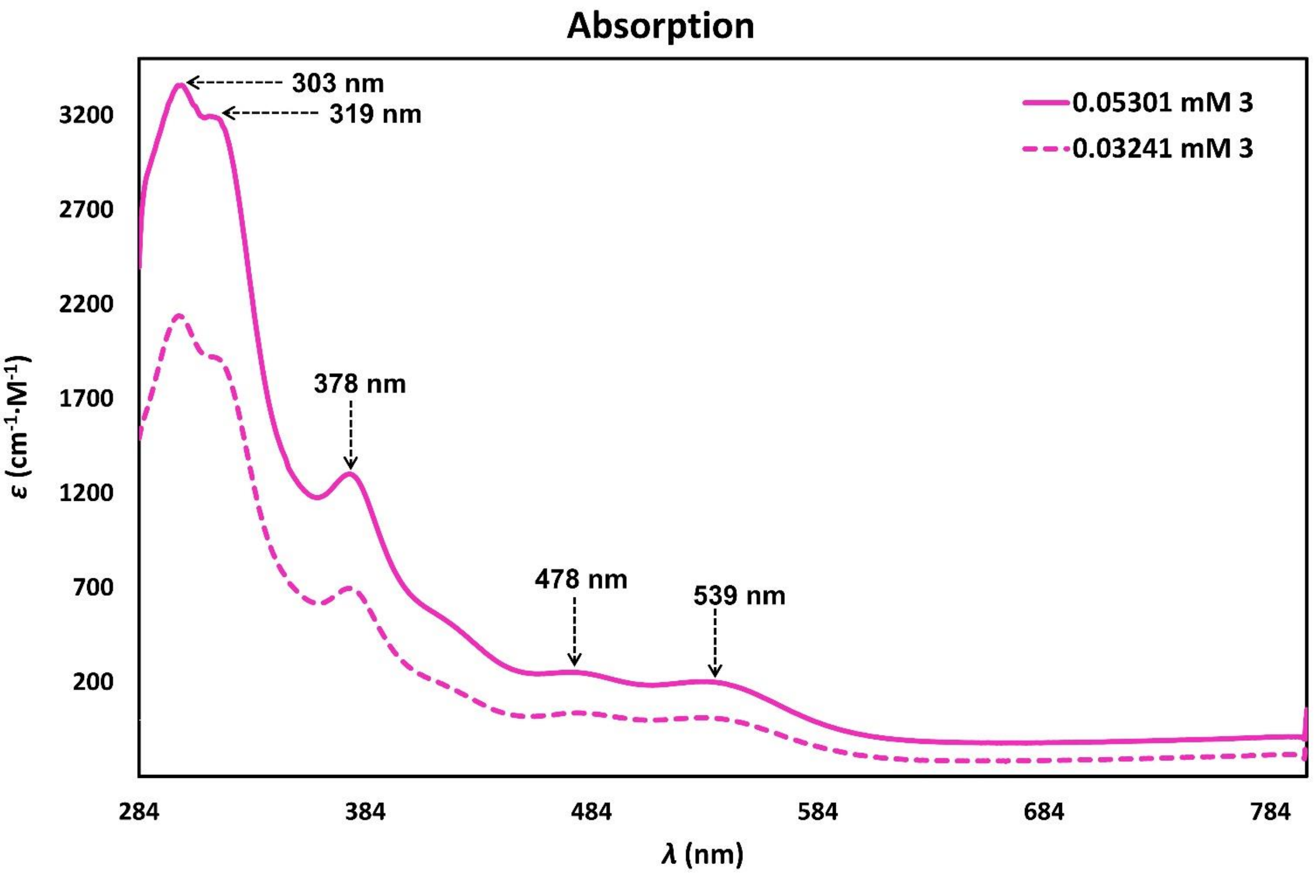

Figure S49: UV-*vis* spectroscopy of [K(18-C-6)(THF)$_2$][(PN)$_2$Zr≡As] (**3**) (pink) in THF in the region of 284 to 800 nm. UV-*vis* spectrum of **3** was collected at different concentrations in THF such as 5.30x10^{-5} M (Pink, solid), and 3.24x10^{-5} M (Pink, dotted).

5.3 Comparative UV-*vis* Spectral Data for [Na(18-C-6)(THF)$_2$][(PN)$_2$Zr≡P] (4), [K(18-C-6)(THF)$_2$][(PN)$_2$Zr≡As] (3), and [K(18-C-6)(THF)$_2$][(PN)$_2$Zr≡Sb] (2) in THF

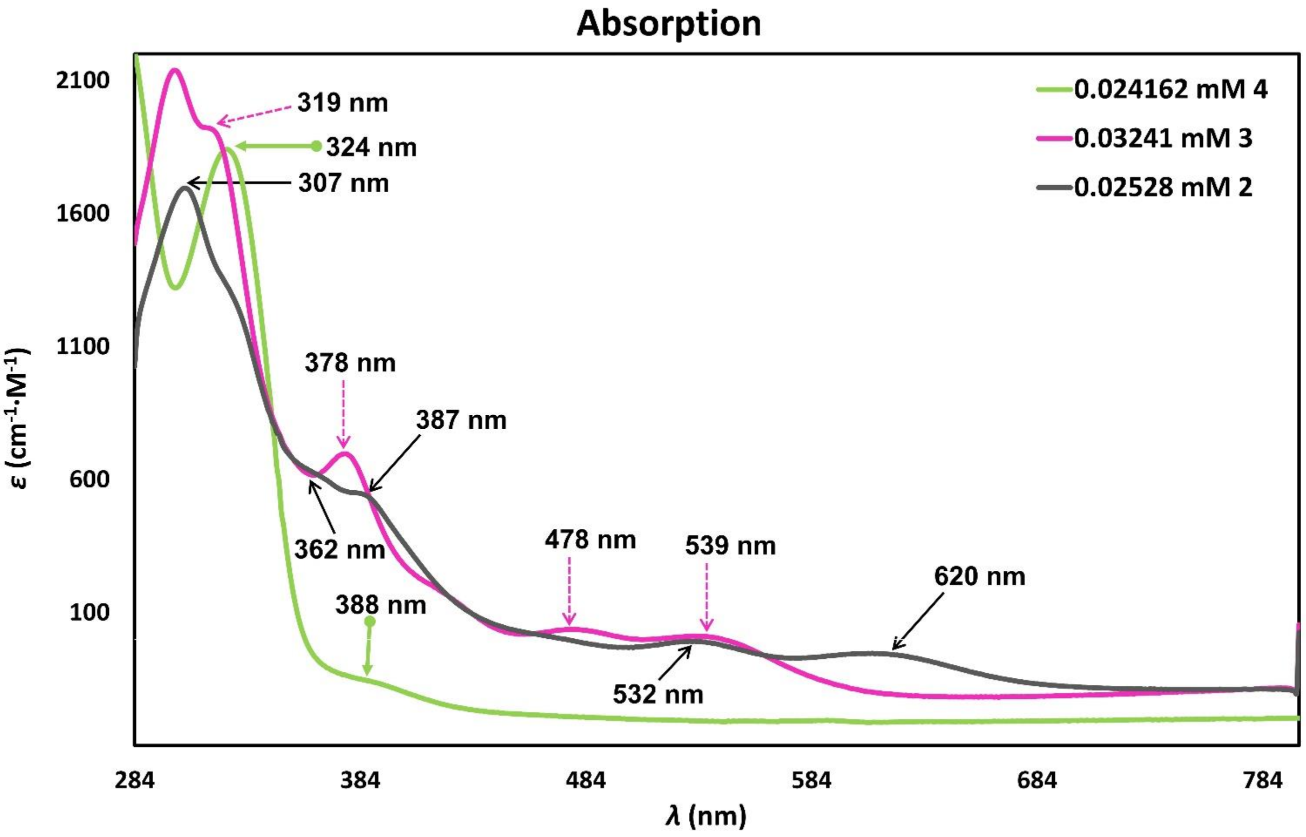

Figure S50: UV-*vis* spectroscopy of [Na(18-C-6)(THF)$_2$][(PN)$_2$Zr≡P] (**4**) *vs.* [K(18-C-6)(THF)$_2$][(PN)$_2$Zr≡As] (**3**) *vs.* [K(18-C-6)(THF)$_2$][(PN)$_2$Zr≡Sb] (**2**) in THF in the region of 284 to 800 nm. UV-*vis* spectra of **4** were recorded with 2.42x10^{-5} M (Yellowish-Green, solid); **3** was recorded with 3.24x10^{-5} M (Pink, solid); and **2** was recorded with 2.53x10^{-5} M (Grey, solid) concentration in THF.

5.4 Comparative UV-*vis* Spectral Data for [Na(18-C-6)(THF)$_2$][(PN)$_2$Zr≡P] (4), [K(18-C-6)(THF)$_2$][(PN)$_2$Zr≡As] (3), and [K(18-C-6)(THF)$_2$][(PN)$_2$Zr≡Sb] (2) in THF with Zoom Region

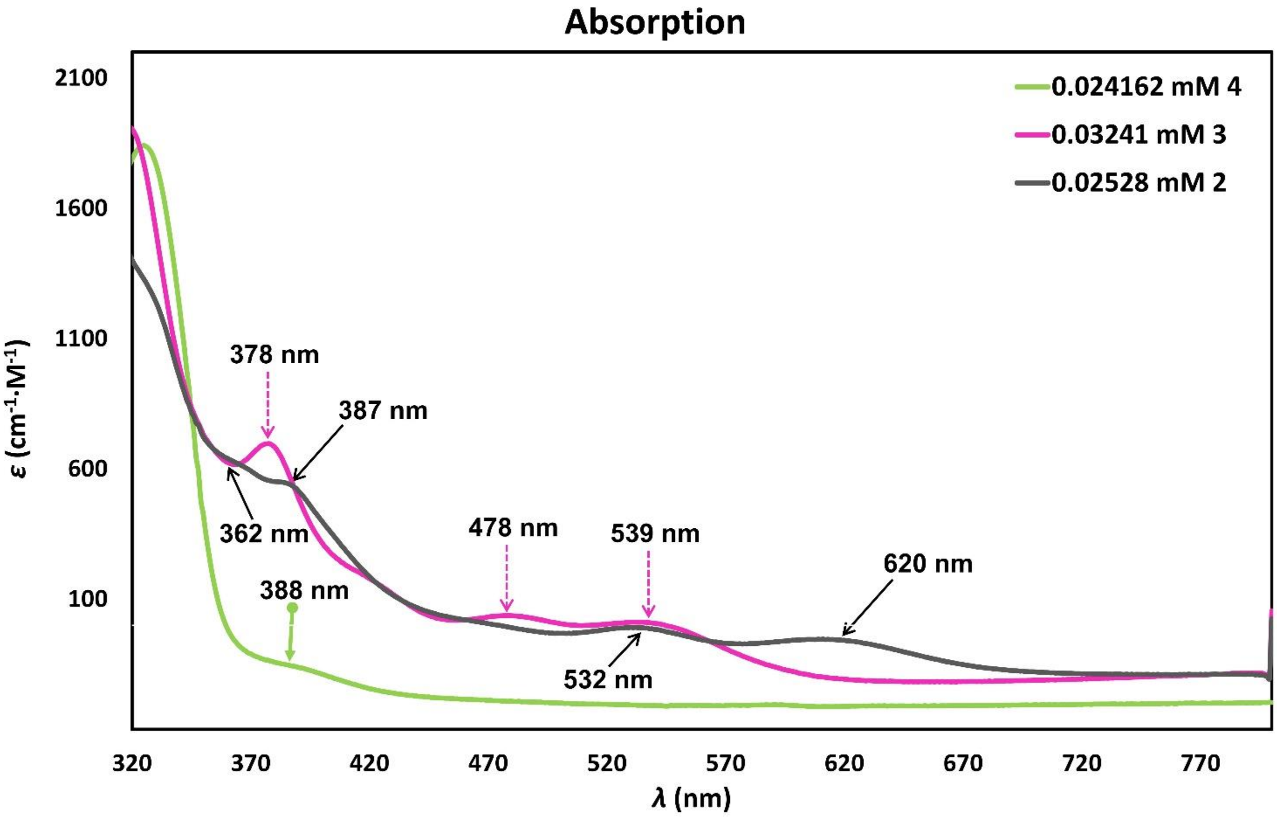

Figure S51: UV-*vis* spectroscopy of [Na(18-C-6)(THF)$_2$][(PN)$_2$Zr≡P] (**4**) (Yellowish-Green, solid) *vs.* [K(18-C-6)(THF)$_2$][(PN)$_2$Zr≡As] (**3**) (Pink, solid) *vs.* [K(18-C-6)(THF)$_2$][(PN)$_2$Zr≡Sb] (**2**) (Grey, solid) in THF in the region of 320 to 800 nm.

5.5 UV-*vis* Spectral Data for [K(18-C-6)][(PN)$_2$Ti-Cl] (8) in toluene.

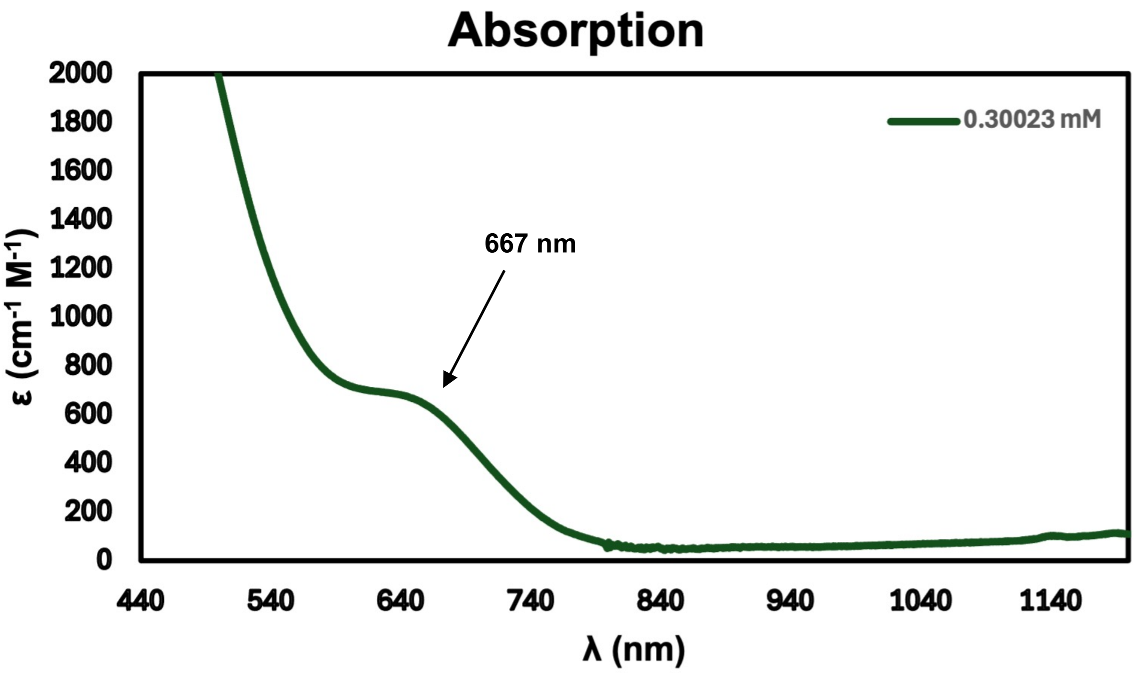

Figure S52: UV-*vis* spectroscopy of [K(18-C-6)][(PN)$_2$Ti–Cl] (**8**) (Green, solid) in toluene.

5.6 UV-*vis* Spectral Data for [K(18-C-6)][$(PN)_2$Ti-Cl] (8) in toluene at Different Region.

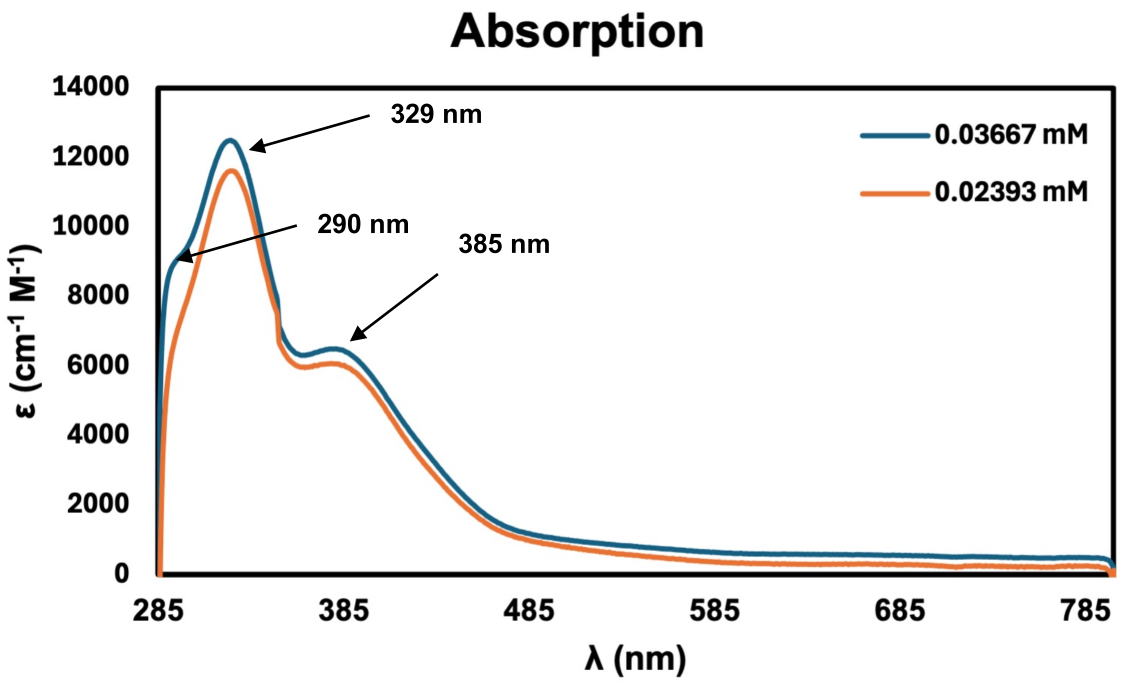

Figure S53: UV-*vis* spectroscopy of [K(18-C-6)][$(PN)_2$Ti–Cl] (**8**) at 0.03667 mM (Blue, solid) and 0.02393 mM (Orange, solid) in toluene.

5.7 Comparative UV-*vis* Spectral Data for [K(crypt)][(PN)$_2$Ti≡P] (6), [K(crypt)][(PN)$_2$Ti≡As] (7) and [K(crypt)][(PN)$_2$Ti≡Sb] (10) in THF

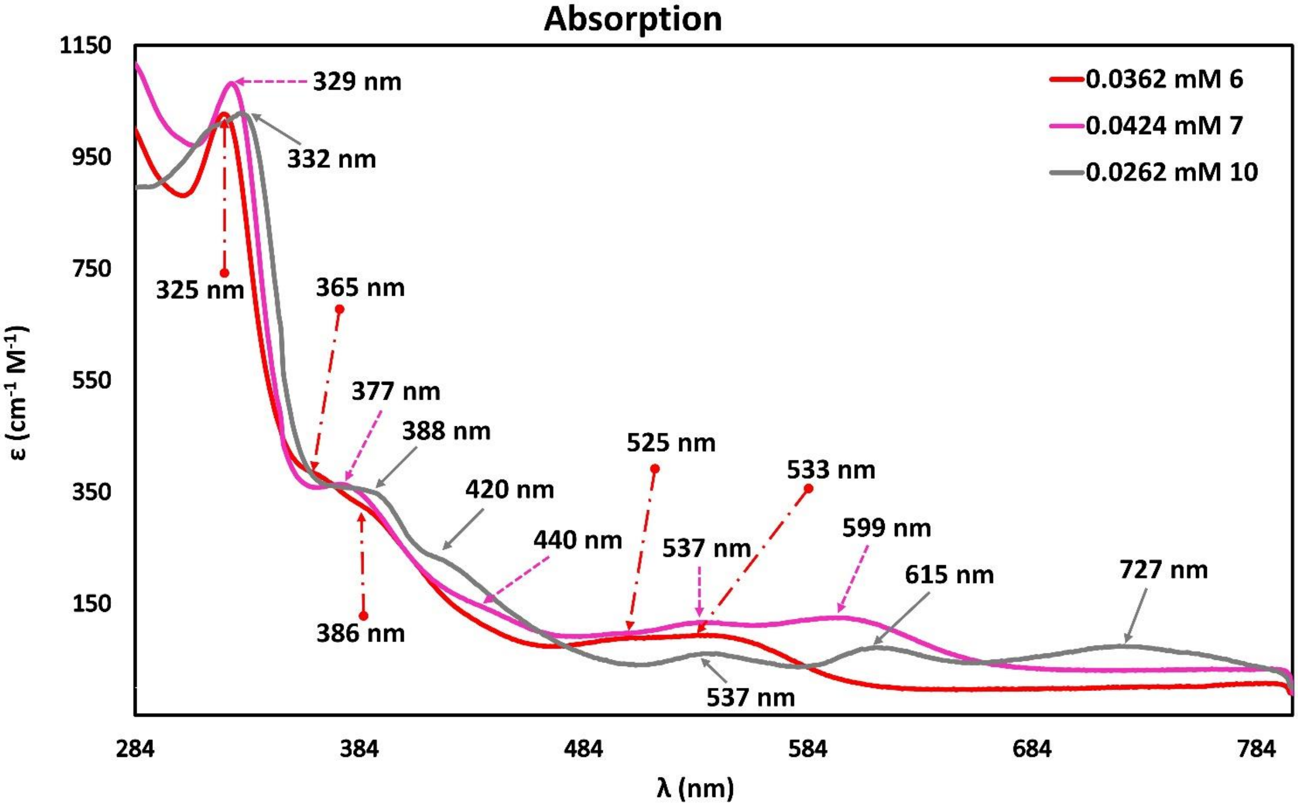

Figure S54: UV-*vis* spectroscopy of [K(crypt)][(PN)$_2$Ti≡P] (**6**) (Red, solid) *vs.* [K(crypt)][(PN)$_2$Ti≡As] (**7**) (Pink, solid) *vs.* [K(crypt)][(PN)$_2$Ti≡Sb] (**10**) (Grey, solid) in THF in the region of 284 to 800 nm.

6. X-Ray Crystallographic Information

Crystallographic data are summarized in Tables S1-S4. Suitable crystals for X-ray analysis of **2-3**, **8-10** were coated with paratone oil, mounted at the end of a MiTeGen cryoloop, and placed in the nitrogen cold stream of the diffractometer. The X-ray intensity data collection for **2**, **3**, **9**, **10** and **8-*THF₂*** was collected on a Rigaku XtaLAB Synergy-S diffractometer[12-13] equipped with an HPC area detector (HyPix-6000HE) and employing confocal multilayer optic-monochromated Cu-Kα radiation (λ=1.54184 Å) at a temperature of 100K. The X-ray intensity data collection for complex **8** was collected on a Rigaku XtaLAB Synergy-i diffractometer[14] equipped with an HPC area detector (HyPix 3000HE) and employing confocal multilayer optic-monochromated Mo-Kα radiation (λ=0.71073 Å) at a temperature of 100K. Preliminary indexing was performed from a series of sixty 0.5° rotation frames with exposures of 0.625 seconds for θ = ±47.554° and θ = ±47.612° for **2** and **9**, respectively, 0.25 seconds for θ = ±47.658° and 1 second for θ = 113.25° for **3**, 2.5 seconds for θ = ±47.612° and 10 seconds for θ = 113.25° for **10**, 0.25 seconds for θ = ±47.156° and 1 second for θ = 113.25° for **8-*THF₂***, while preliminary indexing for **8** was performed from a series of thirty 0.5° rotation frames with exposures of 19.5 seconds. The total of 7352 frames (47 runs) (for **2**), 7206 frames (57 runs) (for **3**), 3398 frames (36 runs) (for **8**), of 5932 frames (48 runs) (for **9**), 5432 frames (46 runs) (for **10**), 7552 frames (48 runs) (for **8-*THF₂***) were collected employing ω scans with a crystal to detector distance of 34.0 mm, rotation widths of 0.5° and exposures of 6.1 seconds (for **2**), 0.76 seconds (for **3**), 0.92 seconds (for **9**), 13.2 seconds (for **10**), 3 seconds (for **8-*THF₂***) respectively, while **8** was collected employing ω scans with a crystal to detector distance of 41.0 mm, rotation widths of 0.5° and exposures of 52 seconds.

Rotation frames were integrated using CrysAlisPro[15–16] producing a listing of unaveraged F^2 and $\sigma(F^2)$ values. The intensity data were corrected for Lorentz and polarization effects and for absorption using SCALE3 ABSPACK[17] (minimum transmission 0.57605 (for **2**), 0.41835 (for **3**), 0.24606 (for **8**), 0.48577 (for **9**), 0.87382 (for **10**), 0.60177 (for **8-*THF₂***) and maximum transmission 1.00000). The initial structures were solved by dual space methods – SHELXT[18]. Refinement was performed by full-matrix least squares based on F^2 using SHELXL[19]. All reflections were used during refinement. Non-hydrogen atoms were refined anisotropically and hydrogen atoms were refined using riding models. The weighting scheme used was $w=1/[\sigma^2(F_o^2)+$

$(0.0865P)^2 + 23.3527P]$ where $P = (F_o^2 + 2F_c^2)/3$ (for **2**); $w=1/[\sigma^2(F_o^2) + (0.1037P)^2 + 8.4451P]$ where $P = (F_o^2 + 2F_c^2)/3$. (for **3**); $w=1/[\sigma^2(F_o^2) + (0.03270P)^2 + 17.8769P]$ where $P = (F_o^2 + 2F_c^2)/3$ (for **8**); $w=1/[\sigma^2(F_o^2) + (0.0466P)^2 + 7.6798P]$ where $P = (F_o^2 + 2F_c^2)/3$ (for **9**); $w=1/[\sigma^2(F_o^2) + (0.1092P)^2 + 35.4583P]$ where $P = (F_o^2 + 2F_c^2)/3$ (for **10**); $w=1/[\sigma^2(F_o^2) + (0.1124P)^2 + 8.1036P]$ where $P = (F_o^2 + 2F_c^2)/3$ (for **8-*THF₂***). The region of disordered on solvent for which a reliable disorder model could not be devised; the X-ray data were corrected for the presence of disordered solvent using the SQUEEZE algorithm.[20] Non-hydrogen atoms were refined anisotropically and hydrogen atoms were refined using a riding model. Olex2[21] was used to generate reports and tables.

These results were checked using the IUCR's CheckCIF routine.

Table S1. Crystallographic data for complexes [K(18-C-6)(THF)$_2$][(PN)$_2$Zr≡Sb] (**2**) and [K(18-C-6)(THF)$_2$][(PN)$_2$Zr≡As] (**3**) as THF solvates.

**Complex**<br>**CCDC Number**	**2·2½THF**<br>**2467015**	**3·4THF**<br>**2467016**
Empirical formula	$C_{74}H_{122}KN_2O_{10.5}P_2SbZr$	$C_{80}H_{134}AsKN_2O_{12}P_2Zr$
Formula weight	1521.74	1583.06
Temperature/K	100	100
Crystal system	triclinic	monoclinic
Space group	P-1	P2/c
a	12.8029(3)Å	12.13250(10)Å
b	13.0246(3)Å	13.05990(10)Å
c	24.7363(5)Å	26.3593(2)Å
α	88.7811(19)°	90°
β	81.6304(19)°	93.8000(10)°
γ	73.365(2)°	90°
Volume	3909.23(17)$Å^3$	4167.43(6)$Å^3$
Z	2	2
d_{calc}	1.293 g/cm^3	1.262 g/cm^3
μ	5.128 mm^{-1}	2.785 mm^{-1}
F(000)	1608.0	1692.0
Crystal size, mm	0.24 × 0.17 × 0.05	0.34 × 0.2 × 0.07
2θ range for data collection	7.086 - 149.004°	6.768 - 148.994°
Index ranges	$-15 \le h \le 12$, $-16 \le k \le 16$, $-30 \le l \le 30$	$-14 \le h \le 15$, $-16 \le k \le 16$, $-32 \le l \le 21$
Reflections collected	87009	99226
Independent reflections	15737[R(int) = 0.1071]	8480[R(int) = 0.0697]
Data/restraints/parameters	15737/231/911	8480/228/484
Goodness-of-fit on F^2	1.058	1.041
Final R indexes [I >=2σ (I)]	$R_1 = 0.0789$, $wR_2 = 0.2047$	$R_1 = 0.0661$, $wR_2 = 0.1813$
Final R indexes [all data]	$R_1 = 0.0956$, $wR_2 = 0.2173$	$R_1 = 0.0696$, $wR_2 = 0.1870$
Largest diff. peak/hole	1.48/-1.49 eÅ^{-3}	0.96/-0.92 eÅ^{-3}

6.1 Molecular Structure for Complex [K(18-C-6)][$(PN)_2$TiCl] (**8**).

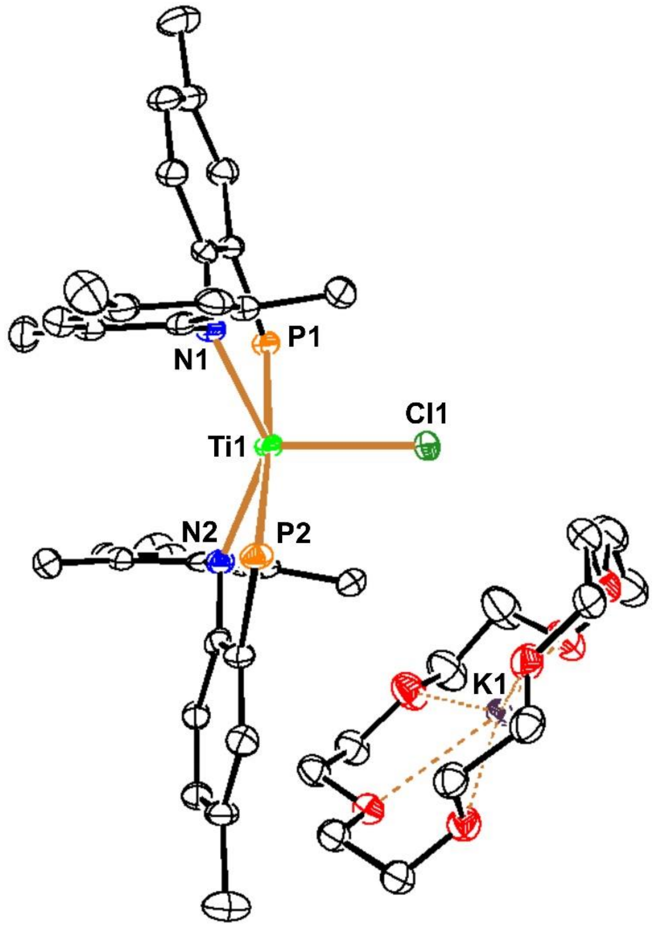

Figure S55: The molecular structure of complex **8** exhibiting mono-nuclear form shows thermal ellipsoids at the 50% probability level. The isopropyl group of PN^{-}=(N-(2-$P^{i}Pr_2$-4-methylphenyl)-2,4,6-$Me_3C_6H_2$ ligand and hydrogen atoms, and residual solvent molecules are omitted for clarity. Selected bond distances [Å] and angles [°]: Ti1-P1, 2.6326(12); Ti1-P2, 2.6438(12); Ti1-N1, 2.151(3); Ti1-N2, 2.170(3); Ti1-Cl1, 2.4687(10); K1-Cl1, 6.018(6); P1-Ti1-P2, 174.92(4); Cl1-Ti1-P1, 92.89(4); Cl1-Ti1-P2, 92.20(4); N1-Ti1-Cl1, 113.70(8); N1-Ti1-P1, 75.53(8); N1-Ti1-P2, 102.43(9); N1-Ti1-N2, 134.39(11); N2-Ti1-Cl1, 111.91(8); N2-Ti1-P1, 102.53(8); N2-Ti1-P2, 75.47(8).

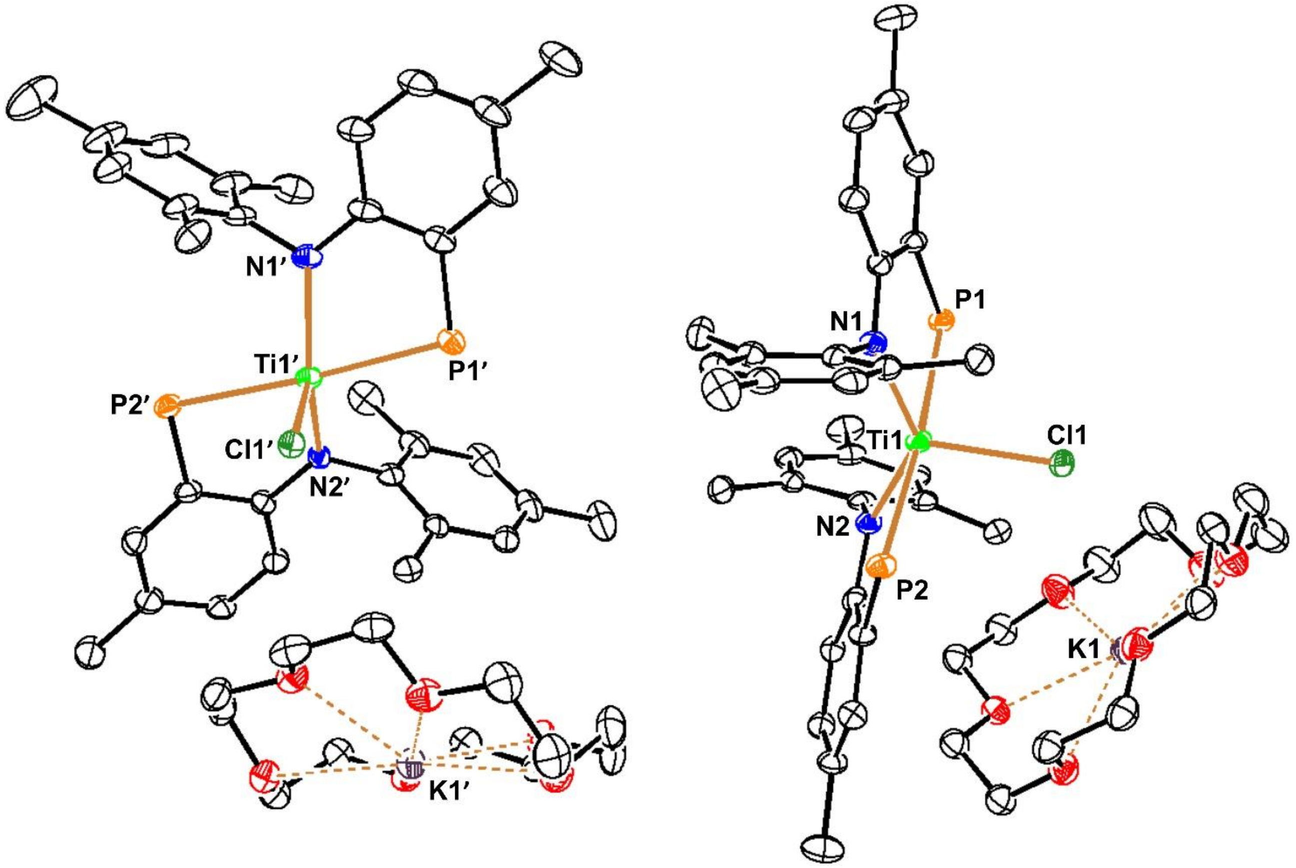

Figure S56: The molecular structure of complex **8** exhibiting binuclear form in the asymmetric unit, shows thermal ellipsoids at the 50% probability level. The isopropyl group of PN^{-}=(N-(2-$P^{i}Pr_2$-4-methylphenyl)-2,4,6-$Me_3C_6H_2$ ligand and hydrogen atoms, and residual solvent molecules are omitted for clarity. Selected bond distances [Å] and angles [°]: Ti1-P1, 2.6326(12); Ti1-P2, 2.6438(12); Ti1-N1, 2.151(3); Ti1-N2, 2.170(3); Ti1-Cl1, 2.4687(10); K1-Cl1, 6.018(6); K1-O6, 2.829(9); K1-O6, 2.813(9); Ti1'-P1', 2.6295(12); Ti1'-P2', 2.6484(12); Ti1'-N1', 2.140(3); Ti1'-N2', 2.163(3); Ti1'-Cl1', 2.4676(10); K1'-Cl1', 6.018(6); K1'-O6', 2.824(3); K1'-O6', 2.891(3); P1-Ti1-P2, 174.92(4); Cl1-Ti1-P1, 92.89(4); Cl1-Ti1-P2, 92.20(4); N1-Ti1-Cl1, 113.70(8); N1-Ti1-P1, 75.53(8); N1-Ti1-P2, 102.43(9); N1-Ti1-N2, 134.39(11); N2-Ti1-Cl1, 111.91(8); N2-Ti1-P1, 102.53(8); N2-Ti1-P2, 75.47(8); P1'-Ti1'-P2', 174.26(4).

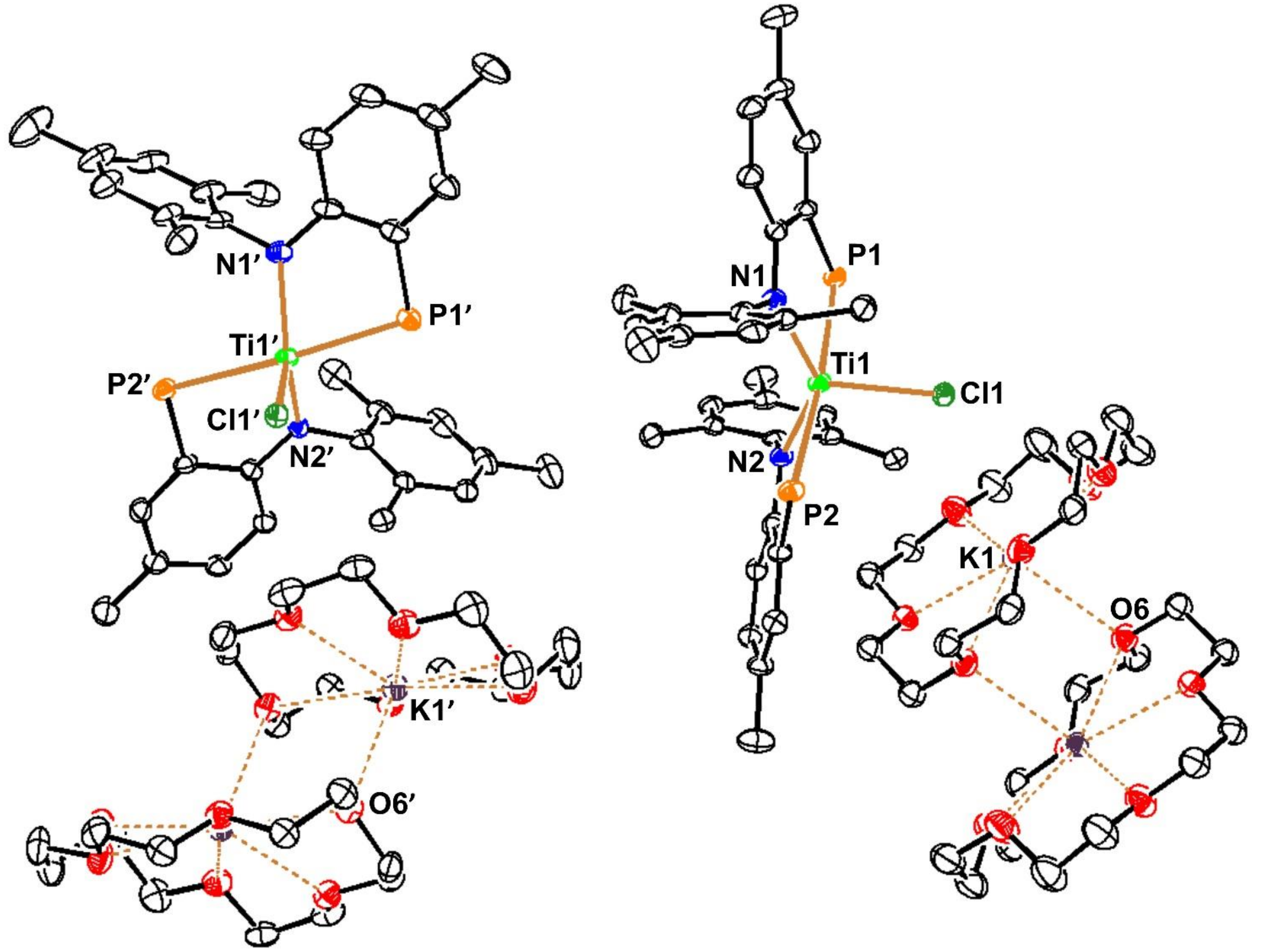

Figure S57: The molecular structure of complex **8** exhibiting dinuclear form and stabilization of the molecule through interaction of crown-ether, 18-C-6 and partially encapsulated K atom, shows thermal ellipsoids at the 50% probability level. The isopropyl group of PN^{-}=(N-(2-P^{i}Pr$_2$-4-methylphenyl)-2,4,6-Me$_3$C$_6$H$_2$ ligand and hydrogen atoms, and residual solvent molecules are omitted for clarity. Selected bond distances [Å] and angles [°]: Ti1-P1, 2.6326(12); Ti1-P2, 2.6438(12); Ti1-N1, 2.151(3); Ti1-N2, 2.170(3); Ti1-Cl1, 2.4687(10); K1-Cl1, 6.018(6); K1-O6, 2.829(9); K1-K1, 4.324(13); K1-O6, 2.813(9); Ti1'-P1', 2.6295(12); Ti1'-P2', 2.6484(12); Ti1'-N1', 2.140(3); Ti1'-N2', 2.163(3); Ti1'-Cl1', 2.4676(10); K1'-Cl1', 6.018(6); K1'-O6', 2.824(3); K1'-K1', 4.4548(17); K1'-O6', 2.891(3); P1-Ti1-P2, 174.92(4); Cl1-Ti1-P1, 92.89(4); Cl1-Ti1-P2, 92.20(4); N1-Ti1-Cl1, 113.70(8); N1-Ti1-P1, 75.53(8); N1-Ti1-P2, 102.43(9); N1-Ti1-N2, 134.39(11); N2-Ti1-Cl1, 111.91(8); N2-Ti1-P1, 102.53(8); N2-Ti1-P2, 75.47(8); P1'-Ti1'-P2', 174.26(4).

6.2 Molecular Structure for Complex [K(18-C-6)(THF)$_2$][(PN)$_2$TiCl] (**8-*THF$_2$***).

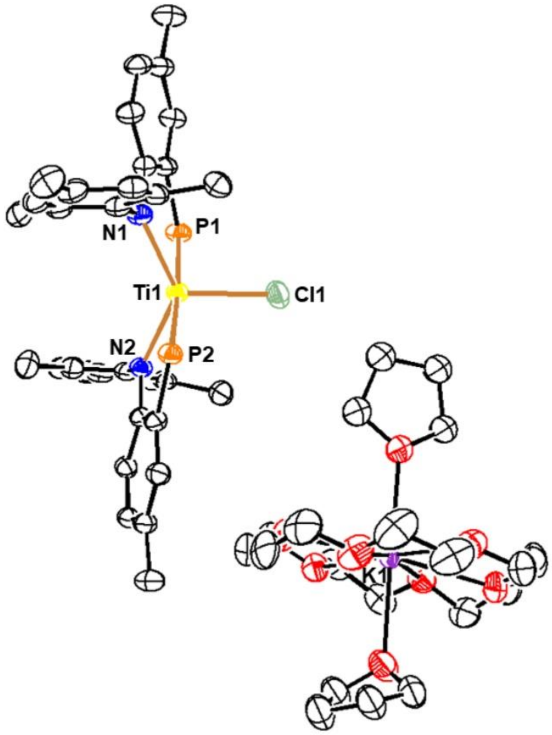

Figure S58: The molecular structure of complex **8-*THF$_2$*** shows thermal ellipsoids at the 50% probability level. The isopropyl group of PN$^-$=(N-(2-P^{i}Pr$_2$-4-methylphenyl)-2,4,6-Me$_3$C$_6$H$_2$ ligand and hydrogen atoms, and residual solvent molecules are omitted for clarity. Selected bond distances [Å] and angles [°]: Ti1-P1, 2.6450(11); Ti1-P2, 2.6310(11); Ti1-N1, 2.149(3); Ti1-N2, 2.148(3); Ti1-Cl1, 2.4387(11); K1-Cl1, 7.494(2); P1-Ti1-P2, 176.89(4); Cl1-Ti1-P1, 91.22(4); Cl1-Ti1-P2, 91.84(4); N1-Ti1-Cl1, 115.78(9); N1-Ti1-P1, 75.69(9); N1-Ti1-P2, 102.41(9); N1-Ti1-N2, 130.55(12); N2-Ti1-Cl1, 113.66(8); N2-Ti1-P1, 103.58(8); N2-Ti1-P2, 75.70(8).

Table S2. Crystallographic data for complexes [K(18-C-6)][(PN)$_2$TiCl] (**8**) and [K(18-C-6)(THF)$_2$][(PN)$_2$TiCl] (**8-*THF$_2$***) as Et_2O and THF solvates.

**Complex**<br>**CCDC Number**	**8·½Et$_2$O**<br>**2467017**	**8-*THF$_2$*·1½THF**<br>**2467935**
Empirical formula	$C_{58}H_{91}ClKN_2O_{6.5}P_2Ti$	$C_{70}H_{114}ClKN_2O_{9.5}P_2Ti$
Formula weight	1104.71	1320.02
Temperature/K	100	100
Crystal system	triclinic	triclinic
Space group	P-1	PT
a	12.8177(3)Å	12.8541(2)Å
b	19.7363(4)Å	13.8223(2)Å
c	24.1350(5)Å	21.4758(3)Å
α	92.0682(18)°	86.5280(10)°
β	93.2603(19)°	75.9060(10)°
γ	95.3595(18)°	85.6720(10)°
Volume	6063.7(2)Å^3	3686.75(10)Å^3
Z	4	2
d_{calc}	1.210 g/cm^3	1.189 g/cm^3
μ	0.355 mm^{-1}	2.653 mm^{-1}
F(000)	2372.0	1424.0
Crystal size, mm	0.2 × 0.2 × 0.2	0.32 × 0.26 × 0.02
2θ range for data collection	6.384 - 54.966°	6.418 - 148.994°
Index ranges	$-16 \leq h \leq 16, -25 \leq k \leq 25, -31 \leq l \leq 31$	$-16 \leq h \leq 16, -17 \leq k \leq 17, -26 \leq l \leq 26$
Reflections collected	192762	88262
Independent reflections	27621[R(int) = 0.0917]	14878[R(int) = 0.0755]
Data/restraints/parameters	27621/655/1521	14878/457/935
Goodness-of-fit on F^2	1.063	1.060
Final R indexes [I >=2σ (I)]	R_1 = 0.0725, wR_2 = 0.1506	R_1 = 0.0767, wR_2 = 0.2220
Final R indexes [all data]	R_1 = 0.1143, wR_2 = 0.1650	R_1 = 0.0880, wR_2 = 0.2311
Largest diff. peak/hole	0.96/-0.87 eÅ^{-3}	0.81/-0.63 eÅ^{-3}

6.3 Molecular Structure for Complex [K(18-C-6)][$(PN)_2$Ti≡Sb] (**9**).

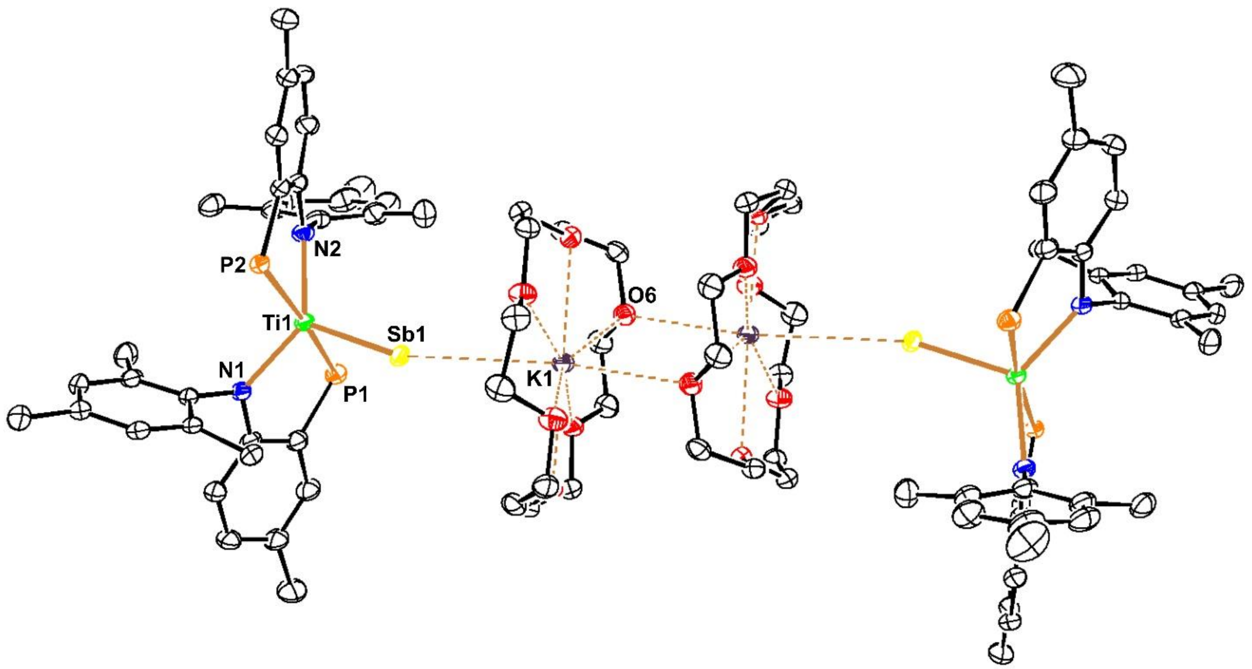

Figure S59: The molecular structure of complex **9** shows thermal ellipsoids at the 50% probability level in the dimeric form, exhibiting ion pair interaction. The isopropyl group of PN^{-}= (N-(2-$P^{i}Pr_2$-4-methylphenyl)-2,4,6-$Me_3C_6H_2$ ligand and hydrogen atoms, and residual solvent molecules are omitted for clarity. Selected bond distances [Å] and angles [°]: Ti1-P1, 2.6370(7), Ti1-P2, 2.6543(7); Ti1-N1, 2.1266(19); Ti1-N2, 2.128(2); Ti1-Sb1, 2.5181(4); K1-Sb1, 3.7289(5); Ti1-Sb1-K1 161.232(13); Sb1-Ti1-P1, 91.113(18); Sb1-Ti1-P2, 94.730(18); P1-Ti1-P2, 174.12(2); N1-Ti1-Sb1, 112.76(5); N1-Ti1-P1, 76.61(6); N1-Ti1-P2, 100.45(6); N1-Ti1-N2, 131.93(8); N2-Ti1-Sb1, 115.30(6).

Table S3. Crystallographic data for complexes [K(18-C-6)][(PN)$_2$Ti≡Sb] (**9**) and [K(crypt)][(PN)$_2$Ti≡Sb] (**10**) as THF and mixture of THF and pentane solvates.

**Complex**<br>**CCDC Number**	**9·THF**<br>**2467018**	**10·2THF·¼Pentane**<br>**2467019**
Empirical formula	$C_{60}H_{94}KN_2O_7P_2SbTi$	$C_{71.25}H_{116}KN_4O_8P_2SbTi$
Formula weight	1226.06	1427.36
Temperature/K	100	100(2)
Crystal system	monoclinic	monoclinic
Space group	$P2_1/c$	I2/a
a	12.70060(10)Å	23.0726(5)Å
b	20.06530(10)Å	26.4303(5)Å
c	24.6100(2)Å	26.3518(6)Å
α	90°	90°
β	98.4070(10)°	110.479(2)°
γ	90°	90°
Volume	6204.25(8)$Å^3$	15054.1(6)$Å^3$
Z	4	8
d_{calc}	1.313 g/cm^3	1.260 g/cm^3
μ	6.027 mm^{-1}	5.057 mm^{-1}
F(000)	2584.0	6060.0
Crystal size, mm	0.27 × 0.17 × 0.08	0.54 × 0.04 × 0.03
2θ range for data collection	7.036 - 149.004°	6.688 - 148.974°
Index ranges	$-15 \le h \le 14$, $-25 \le k \le 25$, $-30 \le l \le 30$	$-28 \le h \le 23$, $-32 \le k \le 33$, $-31 \le l \le 32$
Reflections collected	125901	129365
Independent reflections	12662[R(int) = 0.0746]	15353[R(int) = 0.1011]
Data/restraints/parameters	12662/166/728	15353/713/947
Goodness-of-fit on F^2	1.035	1.058
Final R indexes [I >=2σ (I)]	R_1 = 0.0357, wR_2 = 0.0917	R_1 = 0.0684, wR_2 = 0.1809
Final R indexes [all data]	R_1 = 0.0382, wR_2 = 0.0934	R_1 = 0.0924, wR_2 = 0.1976
Largest diff. peak/hole	0.91/-0.83 eÅ^{-3}	1.07/-1.61 eÅ^{-3}

7. Computational Studies

7.1 Methodology

All calculations were performed using density functional theory (DFT) as implemented in TURBOMOLE software version 7.9.[24] Geometry optimizations were carried out using the PBE functional[25] (fine grids, i.e. gridsize 5[26] plus weight derivatives) combined with dhf-TZVP basis sets[27] assuming C_2 symmetry; the conductor-like screening model was employed.[28] Grimme's D3 method and the Becke-Johnson (D3BJ) damping scheme[29] were used to consider dispersion effects in all calculations. In order to confirm minima, vibration frequencies were carried out at the same level of theory. Cartesian coordinates together with energies and lowest vibration frequencies are collected in file structures.xyz. Further, localized orbitals were constructed by the Pipek-Mezey procedure[30] and Wiberg bond indices[31] were calculated.

Subsequently, chemical shielding constants were calculated at one- as well as at two-component relativistic level using the exact two-component decoupling scheme in the diagonal local approximation for the unitary transformation matrix, 1c-DLU-X2C[32] and 2c-DLU-X2C[33], employing x2c-TZVPall-2c basis sets[34] (other settings as above). Moreover, time-dependent DFT calculations[35] were done at 1c-DLU-X2C and the 2c-DLU-X2C level[36] with the PBE0 functional. Singlet excitation energies, oscillator strengths and dominant orbital contributions are listed in Table S4, images of the frontier orbitals are displayed in Figures S60 and S61.

7.2 Excitation Energies of Ti and Zr

		N		P		As		Sb		Bi	
	irrep	e	o	e	o	e	o	e	o	e	o
Ti											
1	B	2.76	0.01904	1.66	0.00393	1.42	0.00287	1.15	0.00171	1.02	0.00149
2	B	2.96	0.00199	1.93	0.00365	1.51	0.00377	1.51	0.00355	1.43	0.00348
3	A	2.92	0.00008	2.25	0.00004	2.02	0.00002	1.70	0.00022	1.58	0.00014
Zr											
1	B	2.77	0.01122	2.06	0.01122	1.82	0.00822	1.61	0.00359	1.48	0.00271
2	B	3.13	0.00159	2.16	0.00156	1.90	0.00220	1.71	0.00479	1.59	0.00494
3	A	2.82	0.00006	2.46	0.00001	2.24	0.00001	2.00	0.00057	1.88	0.00050
1(2c)		2.66	$7*10^{-7}$	1.83	$9*10^{-8}$	1.54	$3*10^{-7}$	1.06	0.00001	0.76	0.00009

Table S4. Excitation energies e, in eV and oscillator strengths, o, in velocity representation, for the lowest three singlet excitations at 1c-X2C level (i.e. without spin-orbit coupling) and, in row “1(2c)”, for the lowest (triplet-dominated) excitation at 2c-X2C level (i.e. with spin-orbit coupling). The PBE0 functional together with x2c-TZVPall-2c bases was used. The lowest three singlet excitations involve LUMO and HOMO, HOMO-1, HOMO-2. For the shape of orbitals see Figures S60 and S61.

7.3 Data of bond-critical points for the Pn-M bond

M	Pn	Density	Laplacian	EV(1)	EV(2)	EV(3)
Ti	N	2.425E-01	5.639E-01	-4.346E-01	-4.209E-01	1.419E+00
Ti	P	1.083E-01	9.133E-02	-1.066E-01	-9.820E-02	2.961E-01
Ti	As	9.538E-02	8.817E-02	-8.746E-02	-8.003E-02	2.557E-01
Ti	Sb	7.197E-02	5.730E-02	-5.631E-02	-4.943E-02	1.630E-01
Ti	Bi	6.652E-02	7.496E-02	-5.192E-02	-4.482E-02	1.717E-01
Zr	N	2.015E-01	4.612E-01	-2.943E-01	-2.851E-01	1.041E+00
Zr	P	9.979E-02	7.348E-02	-8.604E-02	-7.824E-02	2.378E-01
Zr	As	8.980E-02	6.690E-02	-7.257E-02	-6.515E-02	2.046E-01
Zr	Sb	6.930E-02	4.487E-02	-4.843E-02	-4.211E-02	1.354E-01
Zr	Bi	6.423E-02	6.040E-02	-4.459E-02	-3.818E-02	1.432E-01

Table S5. Density, Laplacian of the density, and the three eigenvalues of the second derivative of the density at the bond-critical points of the Pn-M bond. All quantities in atomic units.

7.4 Computational data for Zr

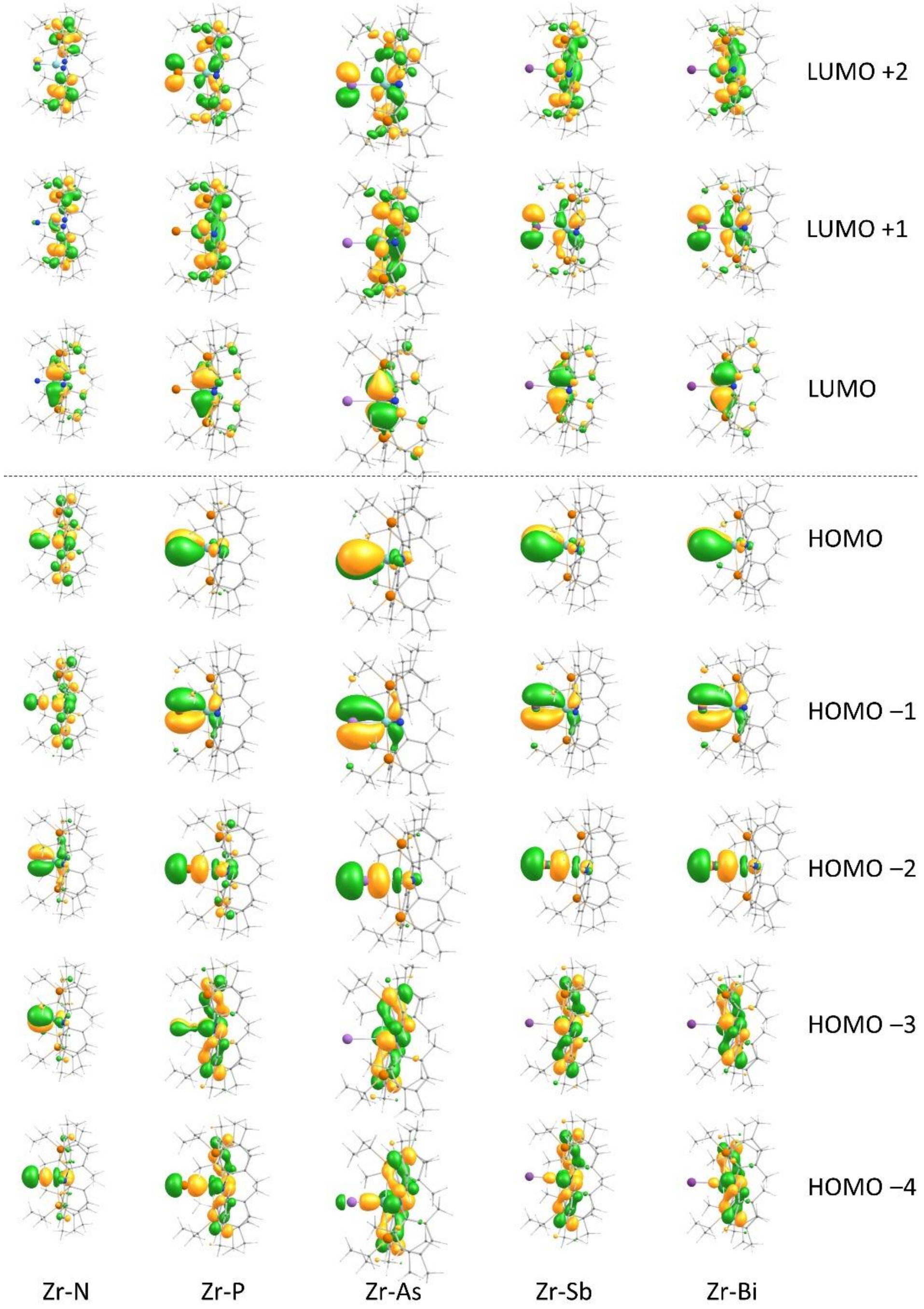

Figure S60: Images of frontier orbitals of the Pn-Zr compounds, Pn=N, P, As, Sb, and hypothetical Bi; unoccupied orbitals above the dashed line, occupied below. Contours are drawn at 0.04 a.u.

7.5 Computational data for Ti

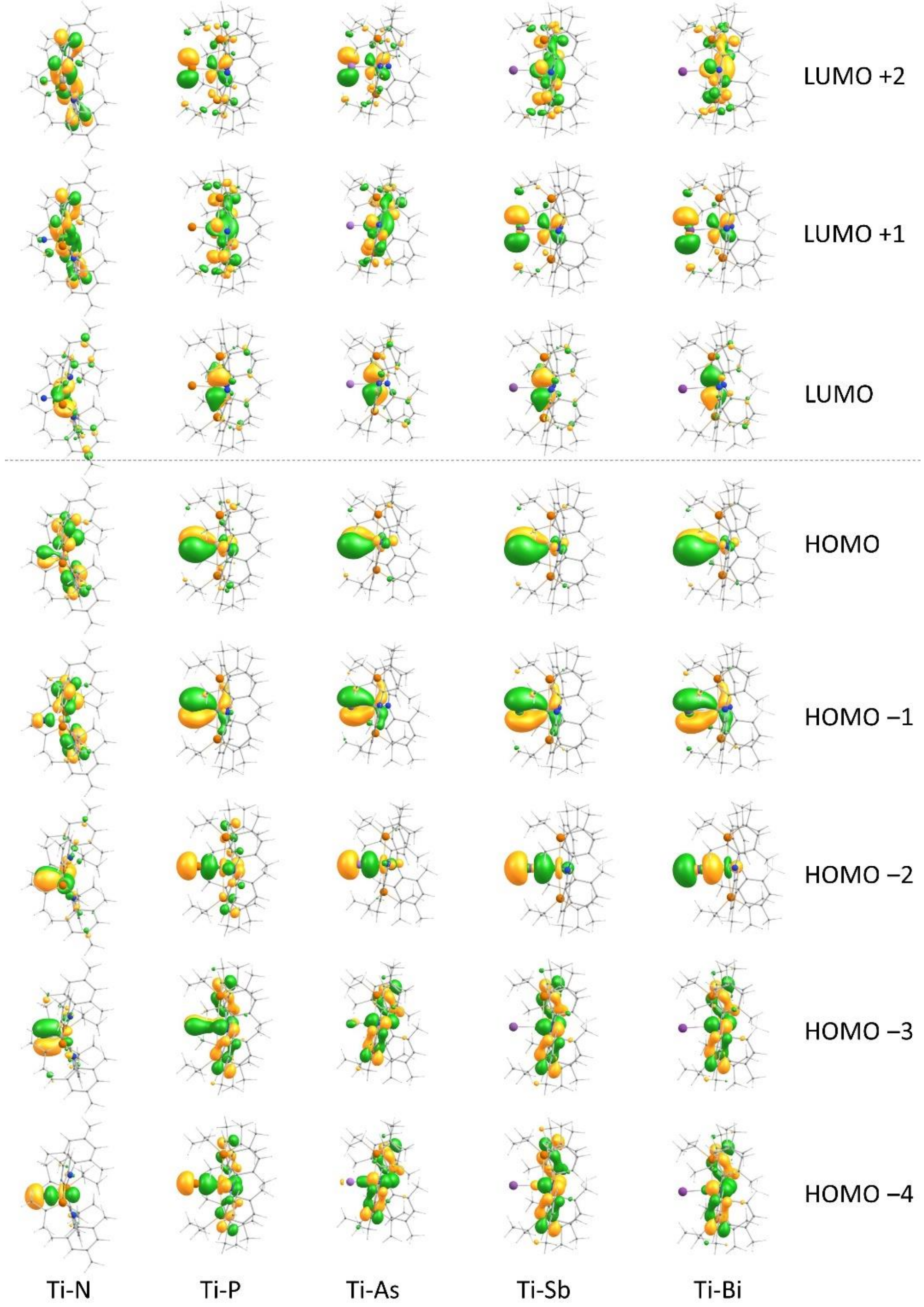

Figure S61: Images of frontier orbitals of the Pn-Ti compounds, Pn=N, P, As, Sb, and hypothetical Bi; unoccupied orbitals above the dashed line, occupied below. Contours are drawn at 0.04 a.u.

7.6 Images of calculated localized orbitals of Pn-Zr compounds

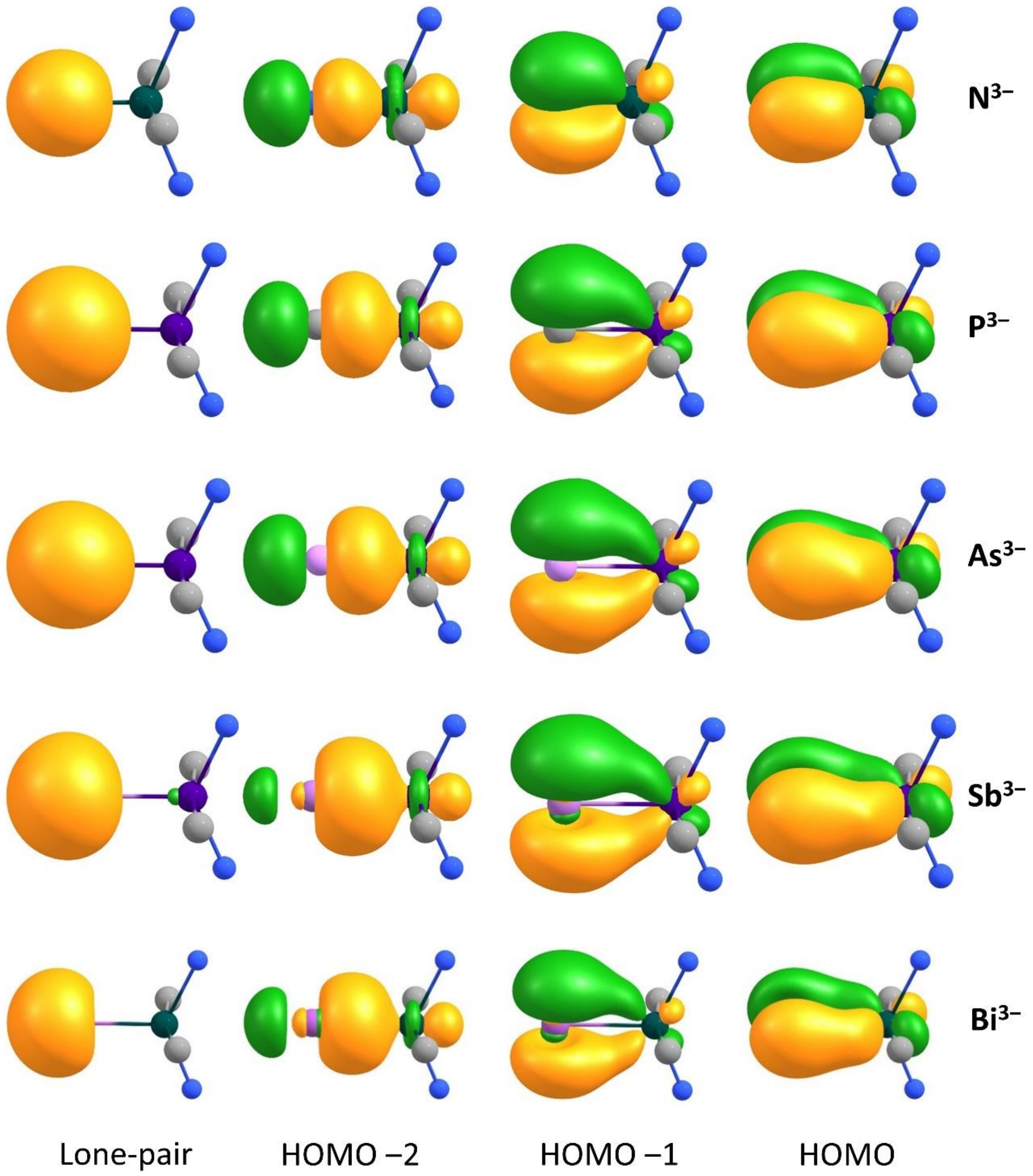

Figure S62: Images of localized orbitals of the Pn-Zr compounds, for Pn=N, P, As, Sb, and hypothetical Bi in rows 1-5 Contours are drawn at 0.05 a.u. C and H atoms are omitted for clarity.

7.7 Images of calculated localized orbitals of Pn-Ti compounds

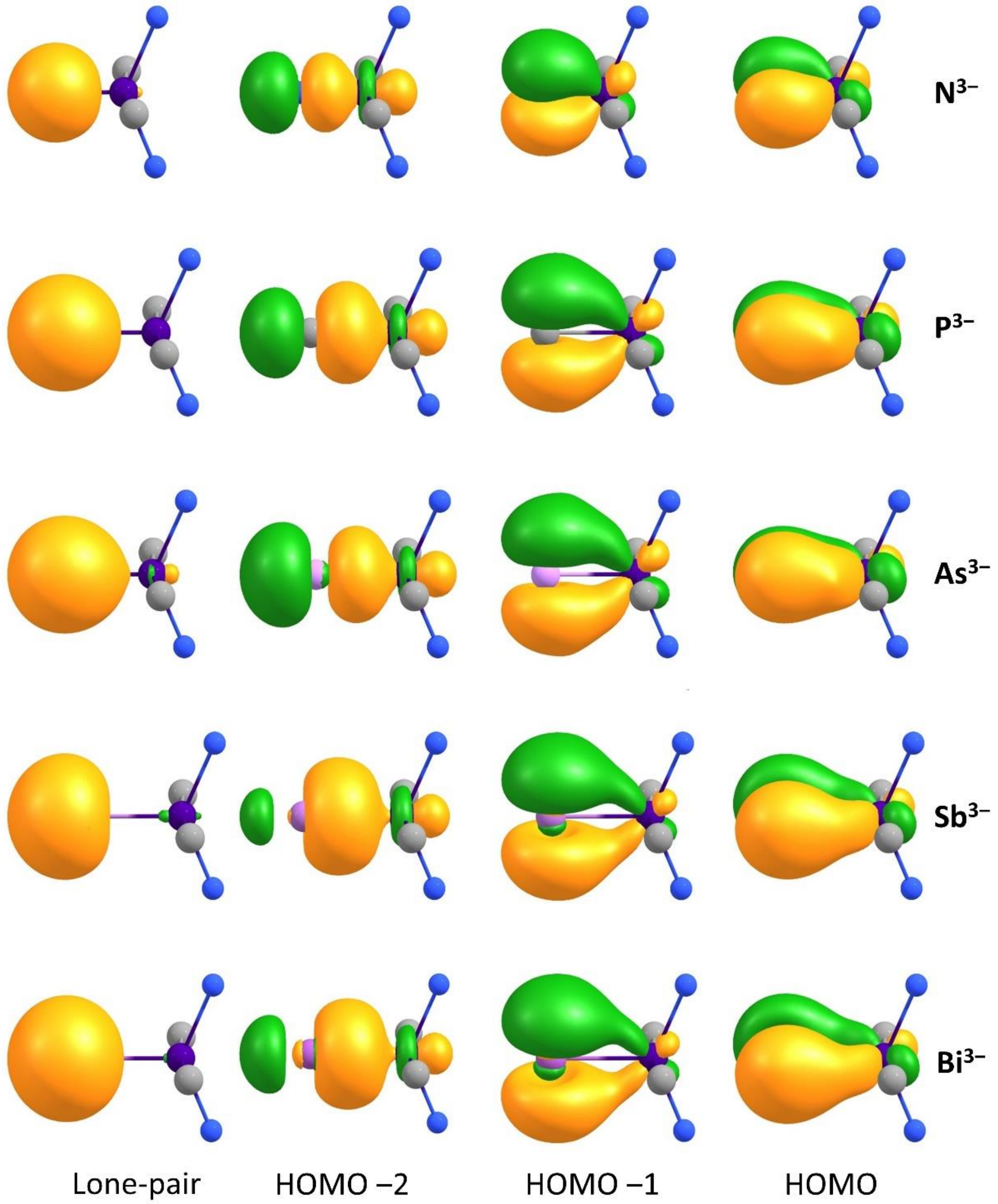

Figure S63: Images of localized orbitals of the Pn-Ti compounds, for Pn=N, P, As, Sb, and hypothetical Bi in rows 1-5 Contours are drawn at 0.05 a.u. C and H atoms are omitted for clarity.

Table S6. Coordinates of the optimized molecular structure for **ZrPn** Series

Zr N: lowest frequency /cm(-1): 16.62; energy /a.u. = -2606.260855605

N	-0.0000000	0.0000000	2.7498721
Zr	-0.0000000	0.0000000	0.9218207
P	-2.2342430	1.6937898	0.6587512
P	2.2342430	-1.6937898	0.6587512
N	-1.7793670	-1.2449173	0.1259972
N	1.7793670	1.2449173	0.1259972
C	-3.5281857	0.4443638	0.3678944
C	-2.3263879	2.8621019	-0.8043159
C	-2.8964868	2.7001060	2.0860863
C	3.5281857	-0.4443638	0.3678944
C	2.3263879	-2.8621019	-0.8043159
C	2.8964868	-2.7001060	2.0860863
C	-3.1041103	-0.8936665	0.0799802
C	-1.4370273	-2.5502737	-0.3184998
C	3.1041103	0.8936665	0.0799802
C	1.4370273	2.5502737	-0.3184998
C	-4.8936109	0.7687544	0.3783938
H	-1.4224379	3.4813322	-0.6820835
C	-3.5485182	3.7767200	-0.8779319
C	-2.1742994	2.0460273	-2.0885745
H	-3.8687101	3.0948306	1.7487521
C	-1.9709974	3.8772600	2.3998442
C	-3.1272348	1.8269064	3.3196574
C	4.8936109	-0.7687544	0.3783938
H	1.4224379	-3.4813322	-0.6820835
C	3.5485182	-3.7767200	-0.8779319
C	2.1742994	-2.0460273	-2.0885745
H	3.8687101	-3.0948306	1.7487521
C	1.9709974	-3.8772600	2.3998442
C	3.1272348	-1.8269064	3.3196574
C	-4.1381059	-1.8211824	-0.2472492
C	-1.2137466	-2.7961462	-1.6954099
C	-1.3209486	-3.6179053	0.6028554
C	4.1381059	1.8211824	-0.2472492
C	1.2137466	2.7961462	-1.6954099
C	1.3209486	3.6179053	0.6028554
C	-5.8992482	-0.1563152	0.0797638
H	-5.1931613	1.7893054	0.6302038
H	-4.4754023	3.1980233	-1.0013403
H	-3.4526190	4.4385168	-1.7538342
H	-3.6549212	4.4152143	0.0093128
H	-1.3065896	1.3753145	-2.0415557
H	-2.0368250	2.7180536	-2.9493153
H	-3.0689594	1.4313960	-2.2681759

H	-2.4181557	4.5022574	3.1884221
H	-1.7890875	4.5145543	1.5235872
H	-0.9975044	3.5195146	2.7633831
H	-2.1831356	1.3686247	3.6514979
H	-3.8438061	1.0193797	3.1152719
H	-3.5259539	2.4430411	4.1407660
C	5.8992482	0.1563152	0.0797638
H	5.1931613	-1.7893054	0.6302038
H	4.4754023	-3.1980233	-1.0013403
H	3.4526190	-4.4385168	-1.7538342
H	3.6549212	-4.4152143	0.0093128
H	1.3065896	-1.3753145	-2.0415557
H	2.0368250	-2.7180536	-2.9493153
H	3.0689594	-1.4313960	-2.2681759
H	2.4181557	-4.5022574	3.1884221
H	1.7890875	-4.5145543	1.5235872
H	0.9975044	-3.5195146	2.7633831
H	2.1831356	-1.3686247	3.6514979
H	3.8438061	-1.0193797	3.1152719
H	3.5259539	-2.4430411	4.1407660
H	-3.8592666	-2.8479338	-0.4914780
C	-5.4790047	-1.4595437	-0.2459940
C	-0.8207943	-4.0726319	-2.1118087
C	-1.4166560	-1.6970753	-2.6999589
C	-0.9133596	-4.8780604	0.1454918
C	-1.6851260	-3.4127050	2.0472653
H	3.8592666	2.8479338	-0.4914780
C	5.4790047	1.4595437	-0.2459940
C	0.8207943	4.0726319	-2.1118087
C	1.4166560	1.6970753	-2.6999589
C	0.9133596	4.8780604	0.1454918
C	1.6851260	3.4127050	2.0472653
C	-7.3577677	0.2219080	0.1038694
C	7.3577677	-0.2219080	0.1038694
H	-6.2290158	-2.2161391	-0.4969874
H	-0.6361359	-4.2453160	-3.1764427
C	-0.6419408	-5.1260469	-1.2053350
H	-2.4733368	-1.3882966	-2.7436177
H	-1.1062775	-2.0185797	-3.7028800
H	-0.8475657	-0.7971563	-2.4251260
H	-0.8112966	-5.6924481	0.8691487
H	-1.1280135	-2.5710424	2.4882617
H	-1.4870318	-4.3192054	2.6346744
H	-2.7537084	-3.1621966	2.1466309
H	6.2290158	2.2161391	-0.4969874
H	0.6361359	4.2453160	-3.1764427
C	0.6419408	5.1260469	-1.2053350
H	2.4733368	1.3882966	-2.7436177
H	1.1062775	2.0185797	-3.7028800
H	0.8475657	0.7971563	-2.4251260
H	0.8112966	5.6924481	0.8691487

H	1.1280135	2.5710424	2.4882617
H	1.4870318	4.3192054	2.6346744
H	2.7537084	3.1621966	2.1466309
H	-7.4820261	1.2931538	0.3160699
H	-7.9131706	-0.3375983	0.8743647
H	-7.8498340	0.0102343	-0.8588987
H	7.4820261	-1.2931538	0.3160699
H	7.9131706	0.3375983	0.8743647
H	7.8498340	-0.0102343	-0.8588987
C	-0.1422265	-6.4699671	-1.6661471
C	0.1422265	6.4699671	-1.6661471
H	-0.4442550	-7.2685281	-0.9741640
H	0.9592964	-6.4819639	-1.7200988
H	-0.5174709	-6.7160163	-2.6698967
H	0.4442550	7.2685281	-0.9741640
H	-0.9592964	6.4819639	-1.7200988
H	0.5174709	6.7160163	-2.6698967

Zr P

lowest frequency /cm(-1): 19.83; energy /a.u. = -2892.774201751

P	0.0000000	-0.0000000	3.2692093
Zr	-0.0000000	-0.0000000	0.9226746
P	1.2279245	-2.4953033	0.6933227
P	-1.2279245	2.4953033	0.6933227
N	2.0650595	0.3372841	0.0931859
N	-2.0650595	-0.3372841	0.0931859
C	2.9363395	-1.9287753	0.4073409
C	0.8121029	-3.5561640	-0.7994961
C	1.3770949	-3.7473348	2.0755626
C	-2.9363395	1.9287753	0.4073409
C	-0.8121029	3.5561640	-0.7994961
C	-1.3770949	3.7473348	2.0755626
C	3.1254455	-0.5505289	0.0835836
C	2.3421509	1.6597153	-0.3699899
C	-3.1254455	0.5505289	0.0835836
C	-2.3421509	-1.6597153	-0.3699899
C	4.0316950	-2.8046776	0.4447244
H	-0.2775118	-3.6918871	-0.7079965
C	1.4769559	-4.9298998	-0.8749096
C	1.0865347	-2.7463917	-2.0662924
H	2.0134619	-4.5467774	1.6634312
C	0.0042677	-4.3355863	2.4085524
C	2.0591807	-3.1851333	3.3206874
C	-4.0316950	2.8046776	0.4447244
H	0.2775118	3.6918871	-0.7079965
C	-1.4769559	4.9298998	-0.8749096
C	-1.0865347	2.7463917	-2.0662924
H	-2.0134619	4.5467774	1.6634312
C	-0.0042677	4.3355863	2.4085524
C	-2.0591807	3.1851333	3.3206874
C	4.4519177	-0.1549360	-0.2432049

C	2.2489739	1.9625216	-1.7498252
C	2.7546134	2.6681722	0.5340209
C	-4.4519177	0.1549360	-0.2432049
C	-2.2489739	-1.9625216	-1.7498252
C	-2.7546134	-2.6681722	0.5340209
C	5.3366893	-2.4003643	0.1440516
H	3.8654214	-3.8499779	0.7169567
H	2.5727968	-4.8465599	-0.9218424
H	1.1443119	-5.4383361	-1.7942354
H	1.2137491	-5.5763769	-0.0272785
H	0.6342551	-1.7483121	-2.0127127
H	0.6661379	-3.2629649	-2.9423572
H	2.1678405	-2.6195015	-2.2234557
H	0.1156535	-5.1445414	3.1469444
H	-0.5031270	-4.7472562	1.5252230
H	-0.6488424	-3.5655987	2.8437996
H	1.4812290	-2.3423067	3.7326087
H	3.0755562	-2.8292609	3.1037475
H	2.1252391	-3.9730596	4.0875149
C	-5.3366893	2.4003643	0.1440516
H	-3.8654214	3.8499779	0.7169567
H	-2.5727968	4.8465599	-0.9218424
H	-1.1443119	5.4383361	-1.7942354
H	-1.2137491	5.5763769	-0.0272785
H	-0.6342551	1.7483121	-2.0127127
H	-0.6661379	3.2629649	-2.9423572
H	-2.1678405	2.6195015	-2.2234557
H	-0.1156535	5.1445414	3.1469444
H	0.5031270	4.7472562	1.5252230
H	0.6488424	3.5655987	2.8437996
H	-1.4812290	2.3423067	3.7326087
H	-3.0755562	2.8292609	3.1037475
H	-2.1252391	3.9730596	4.0875149
H	4.6375696	0.8855913	-0.5144828
C	5.5134948	-1.0516667	-0.2123260
C	2.4775886	3.2731612	-2.1834937
C	1.9448792	0.8823642	-2.7481985
C	2.9600881	3.9702063	0.0586857
C	3.0427552	2.3371756	1.9710618
H	-4.6375696	-0.8855913	-0.5144828
C	-5.5134948	1.0516667	-0.2123260
C	-2.4775886	-3.2731612	-2.1834937
C	-1.9448792	-0.8823642	-2.7481985
C	-2.9600881	-3.9702063	0.0586857
C	-3.0427552	-2.3371756	1.9710618
C	6.4963637	-3.3603500	0.2033169
C	-6.4963637	3.3603500	0.2033169
H	6.5151167	-0.6915322	-0.4660287
H	2.3841802	3.4977926	-3.2501176
C	2.8076214	4.3021568	-1.2924632
H	2.7653662	0.1488948	-2.7990582

H	1.7943511	1.3050099	-3.7501618
H	1.0473014	0.3168733	-2.4655622
H	3.2588969	4.7464772	0.7692896
H	2.2123953	1.7809994	2.4367887
H	3.2374509	3.2491252	2.5509681
H	3.9299451	1.6865862	2.0438930
H	-6.5151167	0.6915322	-0.4660287
H	-2.3841802	-3.4977926	-3.2501176
C	-2.8076214	-4.3021568	-1.2924632
H	-2.7653662	-0.1488948	-2.7990582
H	-1.7943511	-1.3050099	-3.7501618
H	-1.0473014	-0.3168733	-2.4655622
H	-3.2588969	-4.7464772	0.7692896
H	-2.2123953	-1.7809994	2.4367887
H	-3.2374509	-3.2491252	2.5509681
H	-3.9299451	-1.6865862	2.0438930
H	6.1491037	-4.3854652	0.3934930
H	7.2041505	-3.0934052	1.0049902
H	7.0681624	-3.3649074	-0.7378218
H	-6.1491037	4.3854652	0.3934930
H	-7.2041505	3.0934052	1.0049902
H	-7.0681624	3.3649074	-0.7378218
C	2.9649551	5.7217832	-1.7684699
C	-2.9649551	-5.7217832	-1.7684699
H	3.6502898	6.2880975	-1.1224069
H	1.9954678	6.2471867	-1.7568405
H	3.3421672	5.7601790	-2.8002045
H	-3.6502898	-6.2880975	-1.1224069
H	-1.9954678	-6.2471867	-1.7568405
H	-3.3421672	-5.7601790	-2.8002045

Zr As: lowest frequency /cm(-1): 20.04; energy /a.u. = -4787.122248875

As	0.0000000	-0.0000000	3.3732593
Zr	-0.0000000	-0.0000000	0.9310462
P	2.6364537	-0.8620060	0.7039535
P	-2.6364537	0.8620060	0.7039535
N	-0.0496465	-2.0883396	0.0968310
N	0.0496465	2.0883396	0.0968310
C	2.3157765	-2.6328384	0.4125858
C	3.6298142	-0.3034525	-0.7912838
C	3.9063171	-0.8435213	2.0793085
C	-2.3157765	2.6328384	0.4125858
C	-3.6298142	0.3034525	-0.7912838
C	-3.9063171	0.8435213	2.0793085
C	0.9788957	-3.0133658	0.0846286
C	-1.3194000	-2.5486178	-0.3684337
C	-0.9788957	3.0133658	0.0846286
C	1.3194000	2.5486178	-0.3684337
C	3.3376238	-3.5934319	0.4454266
H	3.6119732	0.7944678	-0.7003623
C	2.8652621	-0.6885208	-2.0573849

C	5.0835766	-0.7676965	-0.8705504
H	4.7806363	-1.3673132	1.6608805
C	3.4452235	-1.5959677	3.3248341
C	4.3117583	0.5941287	2.4116010
C	-3.3376238	3.5934319	0.4454266
H	-3.6119732	-0.7944678	-0.7003623
C	-2.8652621	0.6885208	-2.0573849
C	-5.0835766	0.7676965	-0.8705504
H	-4.7806363	1.3673132	1.6608805
C	-3.4452235	1.5959677	3.3248341
C	-4.3117583	-0.5941287	2.4116010
C	0.7745759	-4.3813905	-0.2463817
C	-1.6286175	-2.5033956	-1.7492252
C	-2.2622574	-3.0958124	0.5350604
C	-0.7745759	4.3813905	-0.2463817
C	1.6286175	2.5033956	-1.7492252
C	2.2622574	3.0958124	0.5350604
C	3.1229722	-4.9408468	0.1367931
H	4.3483575	-3.2824523	0.7209649
H	2.8877774	-1.7772882	-2.2131256
H	3.3186718	-0.2025503	-2.9345849
H	1.8145748	-0.3775068	-2.0038917
H	5.6884250	-0.4194862	-0.0228283
H	5.5384567	-0.3625914	-1.7889368
H	5.1563460	-1.8640686	-0.9227617
H	2.5175589	-1.1520859	3.7227781
H	4.2267573	-1.5367809	4.0988735
H	3.2506882	-2.6559042	3.1110728
H	4.6472826	1.1524487	1.5266958
H	5.1338976	0.5885112	3.1439121
H	3.4668650	1.1407920	2.8541472
C	-3.1229722	4.9408468	0.1367931
H	-4.3483575	3.2824523	0.7209649
H	-2.8877774	1.7772882	-2.2131256
H	-3.3186718	0.2025503	-2.9345849
H	-1.8145748	0.3775068	-2.0038917
H	-5.6884250	0.4194862	-0.0228283
H	-5.5384567	0.3625914	-1.7889368
H	-5.1563460	1.8640686	-0.9227617
H	-2.5175589	1.1520859	3.7227781
H	-4.2267573	1.5367809	4.0988735
H	-3.2506882	2.6559042	3.1110728
H	-4.6472826	-1.1524487	1.5266958
H	-5.1338976	-0.5885112	3.1439121
H	-3.4668650	-1.1407920	2.8541472
H	-0.2294408	-4.7115483	-0.5179665
C	1.8129179	-5.3053774	-0.2208236
C	-2.8930748	-2.9149823	-2.1849395
C	-0.5993111	-2.0538017	-2.7465804
C	-3.5214321	-3.4831700	0.0574226
C	-1.8977503	-3.3284090	1.9738826

H	0.2294408	4.7115483	-0.5179665
C	-1.8129179	5.3053774	-0.2208236
C	2.8930748	2.9149823	-2.1849395
C	0.5993111	2.0538017	-2.7465804
C	3.5214321	3.4831700	0.0574226
C	1.8977503	3.3284090	1.9738826
C	4.2394591	-5.9514400	0.1852194
C	-4.2394591	5.9514400	0.1852194
H	1.5980514	-6.3467386	-0.4790524
H	-3.1254877	-2.8577078	-3.2524821
C	-3.8680437	-3.3830819	-1.2949769
H	0.2457744	-2.7594181	-2.7870411
H	-0.1715794	-1.0811903	-2.4698096
H	-1.0344218	-1.9747525	-3.7513703
H	-4.2497070	-3.8862327	0.7674635
H	-1.1089364	-4.0949831	2.0503173
H	-2.7688705	-3.6734838	2.5468030
H	-1.4892029	-2.4182583	2.4466186
H	-1.5980514	6.3467386	-0.4790524
H	3.1254877	2.8577078	-3.2524821
C	3.8680437	3.3830819	-1.2949769
H	-0.2457744	2.7594181	-2.7870411
H	0.1715794	1.0811903	-2.4698096
H	1.0344218	1.9747525	-3.7513703
H	4.2497070	3.8862327	0.7674635
H	1.1089364	4.0949831	2.0503173
H	2.7688705	3.6734838	2.5468030
H	1.4892029	2.4182583	2.4466186
H	4.3498942	-6.4812305	-0.7741178
H	4.0594719	-6.7198269	0.9544904
H	5.1985805	-5.4668852	0.4153800
H	-4.3498942	6.4812305	-0.7741178
H	-4.0594719	6.7198269	0.9544904
H	-5.1985805	5.4668852	0.4153800
C	-5.2500457	-3.7404450	-1.7736566
C	5.2500457	3.7404450	-1.7736566
H	-5.9062600	-2.8540812	-1.7698980
H	-5.7171764	-4.4944456	-1.1246485
H	-5.2316340	-4.1257213	-2.8029836
H	5.9062600	2.8540812	-1.7698980
H	5.7171764	4.4944456	-1.1246485
H	5.2316340	4.1257213	-2.8029836

Zr Sb

lowest frequency /cm(-1): 22.08; energy /a.u. = -2791.833366021

Sb	0.0000000	0.0000000	3.6255560
Zr	0.0000000	-0.0000000	0.9557215
P	1.1014510	2.5478164	0.7207489
P	-1.1014510	-2.5478164	0.7207489
N	-1.5918167	1.3246327	0.1218439
N	1.5918167	-1.3246327	0.1218439

C	-0.4458121	3.4687559	0.4332948
C	2.1581394	2.9116821	-0.7932248
C	1.9807848	3.5172702	2.0618594
C	0.4458121	-3.4687559	0.4332948
C	-2.1581394	-2.9116821	-0.7932248
C	-1.9807848	-3.5172702	2.0618594
C	-1.6116805	2.7121426	0.1100062
C	-2.7741984	0.6769561	-0.3590527
C	1.6116805	-2.7121426	0.1100062
C	2.7741984	-0.6769561	-0.3590527
C	-0.4971615	4.8704406	0.4609499
H	2.9700343	2.1721137	-0.7084173
C	1.3459610	2.5863654	-2.0452579
C	2.7720167	4.3075830	-0.8924710
H	2.1718353	4.5010700	1.6048161
C	1.1427577	3.7342561	3.3180006
C	3.3243477	2.8632426	2.3916290
C	0.4971615	-4.8704406	0.4609499
H	-2.9700343	-2.1721137	-0.7084173
C	-1.3459610	-2.5863654	-2.0452579
C	-2.7720167	-4.3075830	-0.8924710
H	-2.1718353	-4.5010700	1.6048161
C	-3.3243477	-2.8632426	2.3916290
C	-1.1427577	-3.7342561	3.3180006
C	-2.7776443	3.4546875	-0.2178974
C	-2.9323016	0.4291432	-1.7440585
C	-3.8208333	0.3304741	0.5292121
C	2.7776443	-3.4546875	-0.2178974
C	2.9323016	-0.4291432	-1.7440585
C	3.8208333	-0.3304741	0.5292121
C	-1.6549754	5.5930283	0.1541332
H	0.4029164	5.4289611	0.7291241
H	0.8808515	1.5957500	-1.9755526
H	1.9987375	2.5934232	-2.9312224
H	0.5456085	3.3254944	-2.1970215
H	1.9990220	5.0896883	-0.9235448
H	3.3488495	4.3763200	-1.8289025
H	3.4598945	4.5305384	-0.0663158
H	0.2072815	4.2670942	3.0996570
H	1.7201324	4.3310532	4.0417077
H	0.8859680	2.7706811	3.7875581
H	3.1688552	1.8853040	2.8697565
H	3.8851593	3.4997769	3.0932557
H	3.9468606	2.7066496	1.4998010
C	1.6549754	-5.5930283	0.1541332
H	-0.4029164	-5.4289611	0.7291241
H	-0.8808515	-1.5957500	-1.9755526
H	-1.9987375	-2.5934232	-2.9312224
H	-0.5456085	-3.3254944	-2.1970215
H	-1.9990220	-5.0896883	-0.9235448
H	-3.3488495	-4.3763200	-1.8289025

H	-3.4598945	-4.5305384	-0.0663158
H	-3.8851593	-3.4997769	3.0932557
H	-3.9468606	-2.7066496	1.4998010
H	-3.1688552	-1.8853040	2.8697565
H	-0.2072815	-4.2670942	3.0996570
H	-1.7201324	-4.3310532	4.0417077
H	-0.8859680	-2.7706811	3.7875581
H	-3.6872596	2.9146389	-0.4850724
C	-2.7922447	4.8448655	-0.1958918
C	-4.0732402	-0.2414987	-2.1979268
C	-1.9071965	0.9139257	-2.7281983
C	-4.9395540	-0.3527761	0.0332781
C	-3.7805100	0.7533911	1.9696839
H	3.6872596	-2.9146389	-0.4850724
C	2.7922447	-4.8448655	-0.1958918
C	4.0732402	0.2414987	-2.1979268
C	1.9071965	-0.9139257	-2.7281983
C	4.9395540	0.3527761	0.0332781
C	3.7805100	-0.7533911	1.9696839
C	-1.6828106	7.0985899	0.1995895
C	1.6828106	-7.0985899	0.1995895
H	-3.7193951	5.3664061	-0.4517649
H	-4.1726785	-0.4431816	-3.2684800
C	-5.0789263	-0.6699070	-1.3224224
H	-1.8681413	2.0145574	-2.7483393
H	-2.1342840	0.5545454	-3.7402633
H	-0.8989553	0.5780539	-2.4537624
H	-5.7324040	-0.6347666	0.7322291
H	-2.8291916	0.4749997	2.4543457
H	-4.6139682	0.3100823	2.5307218
H	-3.8569690	1.8504778	2.0488602
H	3.7193951	-5.3664061	-0.4517649
H	4.1726785	0.4431816	-3.2684800
C	5.0789263	0.6699070	-1.3224224
H	1.8681413	-2.0145574	-2.7483393
H	2.1342840	-0.5545454	-3.7402633
H	0.8989553	-0.5780539	-2.4537624
H	5.7324040	0.6347666	0.7322291
H	2.8291916	-0.4749997	2.4543457
H	4.6139682	-0.3100823	2.5307218
H	3.8569690	-1.8504778	2.0488602
H	-2.0441508	7.5259462	-0.7487742
H	-0.6806565	7.5050930	0.3943961
H	-2.3535950	7.4681655	0.9921525
H	0.6806565	-7.5050930	0.3943961
H	2.3535950	-7.4681655	0.9921525
H	2.0441508	-7.5259462	-0.7487742
C	-6.2561964	-1.4652329	-1.8201317
C	6.2561964	1.4652329	-1.8201317
H	-7.1316813	-1.3366495	-1.1684642
H	-6.0186848	-2.5419443	-1.8411210

H	-6.5338170	-1.1747345	-2.8432858
H	7.1316813	1.3366495	-1.1684642
H	6.0186848	2.5419443	-1.8411210
H	6.5338170	1.1747345	-2.8432858

Zr Bi: lowest frequency /cm(-1): 20.80; energy /a.u. = -2766.248527189

Bi	0.0000000	0.0000000	3.7021539
Zr	0.0000000	0.0000000	0.9595018
P	1.1021159	2.5452969	0.7283114
P	-1.1021159	-2.5452969	0.7283114
N	-1.5905813	1.3217213	0.1243838
N	1.5905813	-1.3217213	0.1243838
C	-0.4446863	3.4663204	0.4378766
C	2.1619188	2.9080318	-0.7854540
C	1.9830070	3.5189022	2.0667700
C	0.4446863	-3.4663204	0.4378766
C	-2.1619188	-2.9080318	-0.7854540
C	-1.9830070	-3.5189022	2.0667700
C	-1.6101274	2.7098635	0.1127030
C	-2.7719162	0.6746887	-0.3610927
C	1.6101274	-2.7098635	0.1127030
C	2.7719162	-0.6746887	-0.3610927
C	-0.4960300	4.8681287	0.4641945
H	2.9714737	2.1659626	-0.7007557
C	1.3508272	2.5863079	-2.0390965
C	2.7805379	4.3020104	-0.8834656
H	2.1793459	4.4986758	1.6035790
C	1.1460635	3.7497343	3.3208781
C	3.3224984	2.8595518	2.4019542
C	0.4960300	-4.8681287	0.4641945
H	-2.9714737	-2.1659626	-0.7007557
C	-1.3508272	-2.5863079	-2.0390965
C	-2.7805379	-4.3020104	-0.8834656
H	-2.1793459	-4.4986758	1.6035790
C	-3.3224984	-2.8595518	2.4019542
C	-1.1460635	-3.7497343	3.3208781
C	-2.7758435	3.4519724	-0.2162026
C	-2.9260456	0.4287581	-1.7469472
C	-3.8221471	0.3278512	0.5230333
C	2.7758435	-3.4519724	-0.2162026
C	2.9260456	-0.4287581	-1.7469472
C	3.8221471	-0.3278512	0.5230333
C	-1.6533573	5.5904642	0.1548415
H	0.4035789	5.4268248	0.7335789
H	0.8834234	1.5967960	-1.9711602
H	2.0052825	2.5925742	-2.9238653
H	0.5525080	3.3274325	-2.1919747
H	2.0104265	5.0871944	-0.9101870
H	3.3544056	4.3705749	-1.8217825
H	3.4722692	4.5207807	-0.0594277
H	0.2096074	4.2789114	3.0980759

H	1.7239035	4.3548975	4.0372244
H	0.8910558	2.7919860	3.8035102
H	3.1599539	1.8848583	2.8848645
H	3.8863388	3.4963556	3.1008865
H	3.9447277	2.6943208	1.5114562
C	1.6533573	-5.5904642	0.1548415
H	-0.4035789	-5.4268248	0.7335789
H	-0.8834234	-1.5967960	-1.9711602
H	-2.0052825	-2.5925742	-2.9238653
H	-0.5525080	-3.3274325	-2.1919747
H	-2.0104265	-5.0871944	-0.9101870
H	-3.3544056	-4.3705749	-1.8217825
H	-3.4722692	-4.5207807	-0.0594277
H	-3.8863388	-3.4963556	3.1008865
H	-3.9447277	-2.6943208	1.5114562
H	-3.1599539	-1.8848583	2.8848645
H	-0.2096074	-4.2789114	3.0980759
H	-1.7239035	-4.3548975	4.0372244
H	-0.8910558	-2.7919860	3.8035102
H	-3.6853932	2.9117001	-0.4830987
C	-2.7905543	4.8421801	-0.1952318
C	-4.0646201	-0.2429874	-2.2050687
C	-1.8996495	0.9160810	-2.7284847
C	-4.9379183	-0.3573523	0.0229993
C	-3.7908857	0.7545787	1.9624823
H	3.6853932	-2.9117001	-0.4830987
C	2.7905543	-4.8421801	-0.1952318
C	4.0646201	0.2429874	-2.2050687
C	1.8996495	-0.9160810	-2.7284847
C	4.9379183	0.3573523	0.0229993
C	3.7908857	-0.7545787	1.9624823
C	-1.6801872	7.0962143	0.1938063
C	1.6801872	-7.0962143	0.1938063
H	-3.7176646	5.3634925	-0.4516873
H	-4.1602782	-0.4434684	-3.2761936
C	-5.0720775	-0.6744973	-1.3332345
H	-1.8613023	2.0167163	-2.7469774
H	-2.1248831	0.5581202	-3.7414768
H	-0.8917030	0.5804954	-2.4531122
H	-5.7329332	-0.6403159	0.7190911
H	-2.8404112	0.4835496	2.4532256
H	-4.6242049	0.3079801	2.5210811
H	-3.8739612	1.8514096	2.0382007
H	3.7176646	-5.3634925	-0.4516873
H	4.1602782	0.4434684	-3.2761936
C	5.0720775	0.6744973	-1.3332345
H	1.8613023	-2.0167163	-2.7469774
H	2.1248831	-0.5581202	-3.7414768
H	0.8917030	-0.5804954	-2.4531122
H	5.7329332	0.6403159	0.7190911
H	2.8404112	-0.4835496	2.4532256

H	4.6242049	-0.3079801	2.5210811
H	3.8739612	-1.8514096	2.0382007
H	-1.9952493	7.5210881	-0.7722746
H	-0.6872819	7.5014264	0.4333469
H	-2.3871607	7.4696873	0.9520769
H	0.6872819	-7.5014264	0.4333469
H	2.3871607	-7.4696873	0.9520769
H	1.9952493	-7.5210881	-0.7722746
C	-6.2455939	-1.4725591	-1.8353106
C	6.2455939	1.4725591	-1.8353106
H	-7.1248088	-1.3436881	-1.1887229
H	-6.0064983	-2.5490067	-1.8523021
H	-6.5180562	-1.1849926	-2.8606727
H	7.1248088	1.3436881	-1.1887229
H	6.0064983	2.5490067	-1.8523021
H	6.5180562	1.1849926	-2.8606727

Table S7. Coordinates of the optimized molecular structure for **TiPn** Series

Ti N: lowest frequency /cm(-1): 18.87; energy /a.u. = -3408.583306148

N	0.0000000	0.0000000	2.4692718
Ti	0.0000000	0.0000000	0.7999735
P	-1.0157873	2.4787113	0.6366915
P	1.0157873	-2.4787113	0.6366915
N	-2.0085078	-0.2620917	-0.0196875
N	2.0085078	0.2620917	-0.0196875
C	-2.7619407	2.0196492	0.3975315
C	-0.6283557	3.6072905	-0.8110572
C	-1.0813305	3.6394318	2.1015693
C	2.7619407	-2.0196492	0.3975315
C	1.0813305	-3.6394318	2.1015693
C	0.6283557	-3.6072905	-0.8110572
C	-3.0221967	0.6615267	0.0301726
C	-2.3775137	-1.5673731	-0.4421061
C	3.0221967	-0.6615267	0.0301726
C	2.3775137	1.5673731	-0.4421061
C	-3.8124025	2.9423786	0.5035673
H	0.4662082	3.7195393	-0.7520699
C	-0.9627441	2.8649978	-2.1061736
C	-1.2709230	4.9940411	-0.7984348
H	-1.7882198	4.4333173	1.8101355
C	0.2775448	4.2834592	2.3796640
C	-1.6258458	2.9355944	3.3447443
C	3.8124025	-2.9423786	0.5035673
H	1.7882198	-4.4333173	1.8101355
C	-0.2775448	-4.2834592	2.3796640
C	1.6258458	-2.9355944	3.3447443
H	-0.4662082	-3.7195393	-0.7520699
C	0.9627441	-2.8649978	-2.1061736
C	1.2709230	-4.9940411	-0.7984348
C	-4.3803943	0.3468704	-0.2775429

C	-2.8459316	-2.5167984	0.4965440
C	-2.3278699	-1.9155959	-1.8117196
C	4.3803943	-0.3468704	-0.2775429
C	2.8459316	2.5167984	0.4965440
C	2.3278699	1.9155959	-1.8117196
H	-3.5894311	3.9668650	0.8123934
C	-5.1451396	2.6139046	0.2308285
H	-0.5382308	1.8531294	-2.1152054
H	-0.5574139	3.4114753	-2.9714985
H	-2.0520387	2.7756017	-2.2327294
H	-2.3675722	4.9289168	-0.8498270
H	-0.9314172	5.5577951	-1.6825364
H	-1.0010493	5.5793933	0.0906705
H	0.9988363	3.5346827	2.7314126
H	0.1709245	5.0455909	3.1673580
H	0.7044683	4.7692961	1.4919816
H	-2.6457712	2.5600521	3.1822566
H	-1.6486595	3.6410729	4.1904004
H	-0.9879500	2.0788327	3.6102461
H	3.5894311	-3.9668650	0.8123934
C	5.1451396	-2.6139046	0.2308285
H	-0.9988363	-3.5346827	2.7314126
H	-0.1709245	-5.0455909	3.1673580
H	-0.7044683	-4.7692961	1.4919816
H	2.6457712	-2.5600521	3.1822566
H	1.6486595	-3.6410729	4.1904004
H	0.9879500	-2.0788327	3.6102461
H	0.5382308	-1.8531294	-2.1152054
H	0.5574139	-3.4114753	-2.9714985
H	2.0520387	-2.7756017	-2.2327294
H	2.3675722	-4.9289168	-0.8498270
H	0.9314172	-5.5577951	-1.6825364
H	1.0010493	-5.5793933	0.0906705
C	-5.3948766	1.2908580	-0.1773300
H	-4.6258876	-0.6705036	-0.5866977
C	-3.1595327	-3.8117601	0.0662349
C	-3.0613478	-2.1095742	1.9269566
C	-2.6585190	-3.2187879	-2.2030153
C	-1.9660983	-0.8822169	-2.8411387
C	5.3948766	-1.2908580	-0.1773300
H	4.6258876	0.6705036	-0.5866977
C	3.1595327	3.8117601	0.0662349
C	3.0613478	2.1095742	1.9269566
C	2.6585190	3.2187879	-2.2030153
C	1.9660983	0.8822169	-2.8411387
C	-6.2557172	3.6228636	0.3712596
C	6.2557172	-3.6228636	0.3712596
H	-6.4195578	0.9894513	-0.4169507
H	-3.5033233	-4.5429667	0.8042514
C	-3.0538691	-4.1929413	-1.2775998
H	-2.1658049	-1.6181173	2.3390817

H	-3.3245079	-2.9751985	2.5492236
H	-3.8787434	-1.3731496	1.9971101
H	-2.5984616	-3.4816866	-3.2636402
H	-2.7589808	-0.1217676	-2.9307695
H	-1.8164493	-1.3418941	-3.8271120
H	-1.0540942	-0.3393214	-2.5588751
H	6.4195578	-0.9894513	-0.4169507
H	3.5033233	4.5429667	0.8042514
C	3.0538691	4.1929413	-1.2775998
H	3.8787434	1.3731496	1.9971101
H	2.1658049	1.6181173	2.3390817
H	3.3245079	2.9751985	2.5492236
H	2.5984616	3.4816866	-3.2636402
H	2.7589808	0.1217676	-2.9307695
H	1.8164493	1.3418941	-3.8271120
H	1.0540942	0.3393214	-2.5588751
H	-6.8627694	3.6916998	-0.5453581
H	-5.8528788	4.6231683	0.5843345
H	-6.9457477	3.3613589	1.1905505
H	6.9457477	-3.3613589	1.1905505
H	6.8627694	-3.6916998	-0.5453581
H	5.8528788	-4.6231683	0.5843345
C	-3.3295201	-5.6096346	-1.7087270
C	3.3295201	5.6096346	-1.7087270
H	-2.4127530	-6.2208375	-1.6613962
H	-3.6935508	-5.6503163	-2.7451522
H	-4.0735319	-6.0899972	-1.0574424
H	4.0735319	6.0899972	-1.0574424
H	2.4127530	6.2208375	-1.6613962
H	3.6935508	5.6503163	-2.7451522

Ti P: lowest frequency /cm(-1): 24.40 ; energy /a.u. = -3695.083599663

P	0.0000000	0.0000000	3.0155121
Ti	0.0000000	0.0000000	0.8248467
P	-0.9959256	2.4588336	0.7093435
P	0.9959256	-2.4588336	0.7093435
N	-1.9436433	-0.2728601	0.0012631
N	1.9436433	0.2728601	0.0012631
C	-2.7354237	1.9948150	0.4437330
C	-0.6024983	3.5710953	-0.7581778
C	-1.0783621	3.6793657	2.1296559
C	2.7354237	-1.9948150	0.4437330
C	1.0783621	-3.6793657	2.1296559
C	0.6024983	-3.5710953	-0.7581778
C	-2.9763754	0.6465924	0.0469291
C	-2.3136546	-1.5729859	-0.4578210
C	2.9763754	-0.6465924	0.0469291
C	2.3136546	1.5729859	-0.4578210
C	-3.7932422	2.9098916	0.5373142
H	0.4949920	3.6482401	-0.7204354
C	-0.9858877	2.8353792	-2.0422886

C	-1.1931212	4.9806408	-0.7455280
H	-1.6938470	4.5051471	1.7385038
C	0.3093755	4.2277965	2.4649940
C	-1.7680688	3.1219688	3.3721671
C	3.7932422	-2.9098916	0.5373142
H	1.6938470	-4.5051471	1.7385038
C	-0.3093755	-4.2277965	2.4649940
C	1.7680688	-3.1219688	3.3721671
H	-0.4949920	-3.6482401	-0.7204354
C	0.9858877	-2.8353792	-2.0422886
C	1.1931212	-4.9806408	-0.7455280
C	-4.3189350	0.3211004	-0.2952331
C	-2.8277407	-2.5345665	0.4451897
C	-2.2508452	-1.8850613	-1.8358290
C	4.3189350	-0.3211004	-0.2952331
C	2.8277407	2.5345665	0.4451897
C	2.2508452	1.8850613	-1.8358290
H	-3.5847153	3.9316621	0.8640808
C	-5.1153273	2.5732248	0.2257525
H	-0.6116696	1.8043635	-2.0450623
H	-0.5611936	3.3533056	-2.9156870
H	-2.0791542	2.7973117	-2.1587443
H	-2.2929559	4.9601356	-0.7315411
H	-0.8850530	5.5052790	-1.6646685
H	-0.8457555	5.5795993	0.1066731
H	0.9354929	3.4423854	2.9100163
H	0.2187134	5.0454645	3.1968431
H	0.8346789	4.6160637	1.5816094
H	-2.7995087	2.8084869	3.1611669
H	-1.7951056	3.8981408	4.1536941
H	-1.2191936	2.2485621	3.7602417
H	3.5847153	-3.9316621	0.8640808
C	5.1153273	-2.5732248	0.2257525
H	-0.9354929	-3.4423854	2.9100163
H	-0.2187134	-5.0454645	3.1968431
H	-0.8346789	-4.6160637	1.5816094
H	2.7995087	-2.8084869	3.1611669
H	1.7951056	-3.8981408	4.1536941
H	1.2191936	-2.2485621	3.7602417
H	0.6116696	-1.8043635	-2.0450623
H	0.5611936	-3.3533056	-2.9156870
H	2.0791542	-2.7973117	-2.1587443
H	2.2929559	-4.9601356	-0.7315411
H	0.8850530	-5.5052790	-1.6646685
H	0.8457555	-5.5795993	0.1066731
C	-5.3448726	1.2552909	-0.2062468
H	-4.5485968	-0.6924889	-0.6266707
C	-3.1426395	-3.8158526	-0.0253909
C	-3.1156401	-2.1622361	1.8713442
C	-2.5891917	-3.1734470	-2.2666997
C	-1.8804244	-0.8316000	-2.8402852

C	5.3448726	-1.2552909	-0.2062468
H	4.5485968	0.6924889	-0.6266707
C	3.1426395	3.8158526	-0.0253909
C	3.1156401	2.1622361	1.8713442
C	2.5891917	3.1734470	-2.2666997
C	1.8804244	0.8316000	-2.8402852
C	-6.2375132	3.5703490	0.3541369
C	6.2375132	-3.5703490	0.3541369
H	-6.3603288	0.9490409	-0.4754868
H	-3.5166928	-4.5582537	0.6860790
C	-3.0085172	-4.1673717	-1.3740890
H	-2.2728843	-1.6139483	2.3236716
H	-3.3427824	-3.0522617	2.4728767
H	-3.9862292	-1.4867605	1.9170312
H	-2.5156299	-3.4075478	-3.3329397
H	-2.6798725	-0.0781730	-2.9301162
H	-1.7104562	-1.2733349	-3.8309604
H	-0.9795187	-0.2850032	-2.5358094
H	6.3603288	-0.9490409	-0.4754868
H	3.5166928	4.5582537	0.6860790
C	3.0085172	4.1673717	-1.3740890
H	3.9862292	1.4867605	1.9170312
H	2.2728843	1.6139483	2.3236716
H	3.3427824	3.0522617	2.4728767
H	2.5156299	3.4075478	-3.3329397
H	2.6798725	0.0781730	-2.9301162
H	1.7104562	1.2733349	-3.8309604
H	0.9795187	0.2850032	-2.5358094
H	-6.8483551	3.6140398	-0.5609545
H	-5.8475615	4.5789744	0.5503142
H	-6.9195694	3.3111899	1.1806626
H	6.9195694	-3.3111899	1.1806626
H	6.8483551	-3.6140398	-0.5609545
H	5.8475615	-4.5789744	0.5503142
C	-3.2858341	-5.5704326	-1.8450174
C	3.2858341	5.5704326	-1.8450174
H	-2.3729226	-6.1872545	-1.7975647
H	-3.6321275	-5.5826775	-2.8881693
H	-4.0430416	-6.0621607	-1.2180504
H	4.0430416	6.0621607	-1.2180504
H	2.3729226	6.1872545	-1.7975647
H	3.6321275	5.5826775	-2.8881693

Ti As: lowest frequency /cm(-1): 23.01; energy /a.u. = -5589.431071568

As	0.0000000	0.0000000	3.1173550
Ti	0.0000000	-0.0000000	0.8231773
P	-0.9960667	2.4529396	0.7153606
P	0.9960667	-2.4529396	0.7153606
N	-1.9426318	-0.2762622	-0.0001129
N	1.9426318	0.2762622	-0.0001129
C	-2.7356583	1.9906080	0.4442242

C	-0.6012129	3.5628400	-0.7563579
C	-1.0821709	3.6862239	2.1258597
C	2.7356583	-1.9906080	0.4442242
C	1.0821709	-3.6862239	2.1258597
C	0.6012129	-3.5628400	-0.7563579
C	-2.9759306	0.6428856	0.0448260
C	-2.3135131	-1.5764897	-0.4582329
C	2.9759306	-0.6428856	0.0448260
C	2.3135131	1.5764897	-0.4582329
C	-3.7938094	2.9052815	0.5369617
H	0.4966765	3.6336980	-0.7228337
C	-0.9927833	2.8264731	-2.0374815
C	-1.1815055	4.9767422	-0.7467507
H	-1.6811062	4.5164538	1.7191014
C	0.3100052	4.2167125	2.4709078
C	-1.7943182	3.1538342	3.3660489
C	3.7938094	-2.9052815	0.5369617
H	1.6811062	-4.5164538	1.7191014
C	-0.3100052	-4.2167125	2.4709078
C	1.7943182	-3.1538342	3.3660489
H	-0.4966765	-3.6336980	-0.7228337
C	0.9927833	-2.8264731	-2.0374815
C	1.1815055	-4.9767422	-0.7467507
C	-4.3178360	0.3175890	-0.2995219
C	-2.8314102	-2.5369637	0.4439578
C	-2.2496890	-1.8892625	-1.8362418
C	4.3178360	-0.3175890	-0.2995219
C	2.8314102	2.5369637	0.4439578
C	2.2496890	1.8892625	-1.8362418
H	-3.5857923	3.9266226	0.8654572
C	-5.1155657	2.5686815	0.2234896
H	-0.6298979	1.7913501	-2.0355671
H	-0.5631171	3.3358426	-2.9135431
H	-2.0864415	2.7989931	-2.1533256
H	-2.2813407	4.9656146	-0.7214862
H	-0.8787730	5.4936292	-1.6721666
H	-0.8207313	5.5784865	0.0979406
H	0.9202783	3.4226523	2.9228235
H	0.2258414	5.0383847	3.1991551
H	0.8476833	4.5939533	1.5900509
H	-2.8304298	2.8609195	3.1487668
H	-1.8110681	3.9369356	4.1411334
H	-1.2679341	2.2716556	3.7664450
H	3.5857923	-3.9266226	0.8654572
C	5.1155657	-2.5686815	0.2234896
H	-0.9202783	-3.4226523	2.9228235
H	-0.2258414	-5.0383847	3.1991551
H	-0.8476833	-4.5939533	1.5900509
H	2.8304298	-2.8609195	3.1487668
H	1.8110681	-3.9369356	4.1411334
H	1.2679341	-2.2716556	3.7664450

H	0.6298979	-1.7913501	-2.0355671
H	0.5631171	-3.3358426	-2.9135431
H	2.0864415	-2.7989931	-2.1533256
H	2.2813407	-4.9656146	-0.7214862
H	0.8787730	-5.4936292	-1.6721666
H	0.8207313	-5.5784865	0.0979406
C	-5.3444268	1.2512635	-0.2102452
H	-4.5469017	-0.6956478	-0.6324494
C	-3.1451780	-3.8186347	-0.0269207
C	-3.1256034	-2.1641214	1.8685301
C	-2.5884426	-3.1773408	-2.2674569
C	-1.8777939	-0.8362087	-2.8405578
C	5.3444268	-1.2512635	-0.2102452
H	4.5469017	0.6956478	-0.6324494
C	3.1451780	3.8186347	-0.0269207
C	3.1256034	2.1641214	1.8685301
C	2.5884426	3.1773408	-2.2674569
C	1.8777939	0.8362087	-2.8405578
C	-6.2382969	3.5651051	0.3528730
C	6.2382969	-3.5651051	0.3528730
H	-6.3595720	0.9449216	-0.4806100
H	-3.5211832	-4.5605284	0.6841032
C	-3.0089059	-4.1710433	-1.3750601
H	-2.2889534	-1.6062235	2.3224728
H	-3.3463150	-3.0550441	2.4713578
H	-4.0030972	-1.4971941	1.9104611
H	-2.5140658	-3.4114934	-3.3336350
H	-2.6718234	-0.0763263	-2.9230748
H	-1.7172810	-1.2767411	-3.8333332
H	-0.9706774	-0.2973723	-2.5405167
H	6.3595720	-0.9449216	-0.4806100
H	3.5211832	4.5605284	0.6841032
C	3.0089059	4.1710433	-1.3750601
H	4.0030972	1.4971941	1.9104611
H	2.2889534	1.6062235	2.3224728
H	3.3463150	3.0550441	2.4713578
H	2.5140658	3.4114934	-3.3336350
H	2.6718234	0.0763263	-2.9230748
H	1.7172810	1.2767411	-3.8333332
H	0.9706774	0.2973723	-2.5405167
H	-6.8492153	3.6096172	-0.5621437
H	-5.8489099	4.5737322	0.5502597
H	-6.9203191	3.3047830	1.1791034
H	6.9203191	-3.3047830	1.1791034
H	6.8492153	-3.6096172	-0.5621437
H	5.8489099	-4.5737322	0.5502597
C	-3.2858047	-5.5742635	-1.8458850
C	3.2858047	5.5742635	-1.8458850
H	-2.3725602	-6.1906849	-1.7992008
H	-3.6328856	-5.5866860	-2.8887896
H	-4.0423335	-6.0664493	-1.2184362

H	4.0423335	6.0664493	-1.2184362
H	2.3725602	6.1906849	-1.7992008
H	3.6328856	5.5866860	-2.8887896

Ti Sb: lowest frequency /cm(-1): 26.44; energy /a.u. = -3594.141777382

Sb	0.0000000	0.0000000	3.3716545
Ti	-0.0000000	0.0000000	0.8478320
P	-0.9948423	2.4577233	0.7343737
P	0.9948423	-2.4577233	0.7343737
N	-1.9310053	-0.2855305	0.0402816
N	1.9310053	0.2855305	0.0402816
C	-2.7313297	1.9869488	0.4607453
C	-0.5930958	3.5435555	-0.7562242
C	-1.0906371	3.7318646	2.1106207
C	2.7313297	-1.9869488	0.4607453
C	1.0906371	-3.7318646	2.1106207
C	0.5930958	-3.5435555	-0.7562242
C	-2.9678153	0.6367016	0.0724858
C	-2.3005852	-1.5808741	-0.4417929
C	2.9678153	-0.6367016	0.0724858
C	2.3005852	1.5808741	-0.4417929
C	-3.7902518	2.9028718	0.5317880
H	0.5045054	3.6128394	-0.7184749
C	-0.9807918	2.7884103	-2.0265786
C	-1.1697339	4.9589318	-0.7746443
H	-1.6702230	4.5534524	1.6613913
C	0.3016640	4.2576476	2.4619009
C	-1.8365142	3.2661474	3.3567011
C	3.7902518	-2.9028718	0.5317880
H	1.6702230	-4.5534524	1.6613913
C	-0.3016640	-4.2576476	2.4619009
C	1.8365142	-3.2661474	3.3567011
H	-0.5045054	-3.6128394	-0.7184749
C	0.9807918	-2.7884103	-2.0265786
C	1.1697339	-4.9589318	-0.7746443
C	-4.3055198	0.3049120	-0.2759622
C	-2.8213036	-2.5577069	0.4412889
C	-2.2425964	-1.8670737	-1.8260976
C	4.3055198	-0.3049120	-0.2759622
C	2.8213036	2.5577069	0.4412889
C	2.2425964	1.8670737	-1.8260976
H	-3.5854194	3.9288905	0.8468899
C	-5.1087427	2.5615339	0.2103632
H	-0.6161228	1.7546712	-2.0077883
H	-0.5489528	3.2852301	-2.9086616
H	-2.0740084	2.7573190	-2.1452588
H	-2.2695557	4.9520950	-0.7449407
H	-0.8693687	5.4523330	-1.7135109
H	-0.8026283	5.5798237	0.0531830
H	0.8948347	3.4715600	2.9500124
H	0.2146978	5.1033652	3.1616161

H	0.8578676	4.5999296	1.5782843
H	-2.8612701	2.9444094	3.1276443
H	-1.8869915	4.0959735	4.0798712
H	-1.3138346	2.4219295	3.8351501
H	3.5854194	-3.9288905	0.8468899
C	5.1087427	-2.5615339	0.2103632
H	-0.8948347	-3.4715600	2.9500124
H	-0.2146978	-5.1033652	3.1616161
H	-0.8578676	-4.5999296	1.5782843
H	2.8612701	-2.9444094	3.1276443
H	1.8869915	-4.0959735	4.0798712
H	1.3138346	-2.4219295	3.8351501
H	0.6161228	-1.7546712	-2.0077883
H	0.5489528	-3.2852301	-2.9086616
H	2.0740084	-2.7573190	-2.1452588
H	2.2695557	-4.9520950	-0.7449407
H	0.8693687	-5.4523330	-1.7135109
H	0.8026283	-5.5798237	0.0531830
C	-5.3339272	1.2387215	-0.2069465
H	-4.5314261	-0.7119630	-0.5992024
C	-3.1345821	-3.8304924	-0.0537326
C	-3.1271510	-2.2155237	1.8705838
C	-2.5808255	-3.1470847	-2.2810371
C	-1.8789635	-0.7979208	-2.8154676
C	5.3339272	-1.2387215	-0.2069465
H	4.5314261	0.7119630	-0.5992024
C	3.1345821	3.8304924	-0.0537326
C	3.1271510	2.2155237	1.8705838
C	2.5808255	3.1470847	-2.2810371
C	1.8789635	0.7979208	-2.8154676
C	-6.2322206	3.5595841	0.3165917
C	6.2322206	-3.5595841	0.3165917
H	-6.3463921	0.9281868	-0.4819250
H	-3.5123638	-4.5842978	0.6435247
C	-2.9978773	-4.1584638	-1.4076504
H	-2.2887609	-1.6871200	2.3538521
H	-3.3720754	-3.1175895	2.4468308
H	-3.9919237	-1.5327678	1.9185165
H	-2.5089126	-3.3596200	-3.3518027
H	-2.6750320	-0.0387894	-2.8821289
H	-1.7230110	-1.2233858	-3.8154352
H	-0.9713897	-0.2622537	-2.5134774
H	6.3463921	-0.9281868	-0.4819250
H	3.5123638	4.5842978	0.6435247
C	2.9978773	4.1584638	-1.4076504
H	3.9919237	1.5327678	1.9185165
H	2.2887609	1.6871200	2.3538521
H	3.3720754	3.1175895	2.4468308
H	2.5089126	3.3596200	-3.3518027
H	2.6750320	0.0387894	-2.8821289
H	1.7230110	1.2233858	-3.8154352

H	0.9713897	0.2622537	-2.5134774
H	-6.8539887	3.5677818	-0.5918481
H	-5.8436230	4.5756750	0.4725562
H	-6.9028053	3.3275587	1.1604211
H	6.9028053	-3.3275587	1.1604211
H	6.8539887	-3.5677818	-0.5918481
H	5.8436230	-4.5756750	0.4725562
C	-3.2735911	-5.5526907	-1.9042781
C	3.2735911	5.5526907	-1.9042781
H	-2.3587862	-6.1676249	-1.8718681
H	-3.6234856	-5.5458334	-2.9462677
H	-4.0271828	-6.0581347	-1.2839710
H	4.0271828	6.0581347	-1.2839710
H	2.3587862	6.1676249	-1.8718681
H	3.6234856	5.5458334	-2.9462677

Ti Bi: lowest frequency /cm(-1): 23.67; energy /a.u. = -3568.557372103

Bi	-0.0000000	0.0000000	3.4491837
Ti	0.0000000	0.0000000	0.8534733
P	-0.9947376	2.4569788	0.7446345
P	0.9947376	-2.4569788	0.7446345
N	-1.9311155	-0.2881207	0.0491503
N	1.9311155	0.2881207	0.0491503
C	-2.7309870	1.9850096	0.4702579
C	-0.5920500	3.5396575	-0.7493978
C	-1.0926904	3.7406249	2.1132444
C	2.7309870	-1.9850096	0.4702579
C	1.0926904	-3.7406249	2.1132444
C	0.5920500	-3.5396575	-0.7493978
C	-2.9677153	0.6349707	0.0814074
C	-2.3007317	-1.5820571	-0.4375858
C	2.9677153	-0.6349707	0.0814074
C	2.3007317	1.5820571	-0.4375858
C	-3.7899690	2.9013002	0.5395264
H	0.5055642	3.6082308	-0.7115187
C	-0.9797625	2.7810984	-2.0175137
C	-1.1674119	4.9555063	-0.7731128
H	-1.6686348	4.5598943	1.6554708
C	0.2998357	4.2656096	2.4648738
C	-1.8444871	3.2903758	3.3612965
C	3.7899690	-2.9013002	0.5395264
H	1.6686348	-4.5598943	1.6554708
C	-0.2998357	-4.2656096	2.4648738
C	1.8444871	-3.2903758	3.3612965
H	-0.5055642	-3.6082308	-0.7115187
C	0.9797625	-2.7810984	-2.0175137
C	1.1674119	-4.9555063	-0.7731128
C	-4.3053882	0.3032833	-0.2669363
C	-2.8221587	-2.5625215	0.4411676
C	-2.2427053	-1.8632708	-1.8229966
C	4.3053882	-0.3032833	-0.2669363

C	2.8221587	2.5625215	0.4411676
C	2.2427053	1.8632708	-1.8229966
H	-3.5853787	3.9273473	0.8546135
C	-5.1081396	2.5604567	0.2167659
H	-0.6159802	1.7471881	-1.9948635
H	-0.5469858	3.2745903	-2.9010155
H	-2.0729310	2.7503304	-2.1367664
H	-2.2672027	4.9500582	-0.7409184
H	-0.8687154	5.4441030	-1.7150568
H	-0.7977432	5.5801852	0.0507200
H	0.8887049	3.4814640	2.9617978
H	0.2132198	5.1174154	3.1572017
H	0.8602662	4.5984670	1.5803242
H	-2.8653303	2.9574541	3.1311430
H	-1.9055597	4.1319687	4.0699084
H	-1.3200461	2.4595800	3.8610028
H	3.5853787	-3.9273473	0.8546135
C	5.1081396	-2.5604567	0.2167659
H	-0.8887049	-3.4814640	2.9617978
H	-0.2132198	-5.1174154	3.1572017
H	-0.8602662	-4.5984670	1.5803242
H	2.8653303	-2.9574541	3.1311430
H	1.9055597	-4.1319687	4.0699084
H	1.3200461	-2.4595800	3.8610028
H	0.6159802	-1.7471881	-1.9948635
H	0.5469858	-3.2745903	-2.9010155
H	2.0729310	-2.7503304	-2.1367664
H	2.2672027	-4.9500582	-0.7409184
H	0.8687154	-5.4441030	-1.7150568
H	0.7977432	-5.5801852	0.0507200
C	-5.3337336	1.2371978	-0.1991290
H	-4.5314443	-0.7139582	-0.5889082
C	-3.1339684	-3.8338231	-0.0586779
C	-3.1308342	-2.2267707	1.8711874
C	-2.5795383	-3.1418919	-2.2828284
C	-1.8802307	-0.7906660	-2.8089102
C	5.3337336	-1.2371978	-0.1991290
H	4.5314443	0.7139582	-0.5889082
C	3.1339684	3.8338231	-0.0586779
C	3.1308342	2.2267707	1.8711874
C	2.5795383	3.1418919	-2.2828284
C	1.8802307	0.7906660	-2.8089102
C	-6.2305797	3.5604053	0.3160784
C	6.2305797	-3.5604053	0.3160784
H	-6.3461821	0.9263564	-0.4738072
H	-3.5119967	-4.5902151	0.6356339
C	-2.9958062	-4.1570419	-1.4135230
H	-2.2918487	-1.7043296	2.3600063
H	-3.3799185	-3.1309445	2.4423288
H	-3.9931019	-1.5410679	1.9208379
H	-2.5073284	-3.3501249	-3.3544127

H	-2.6730267	-0.0275192	-2.8670416
H	-1.7317514	-1.2118942	-3.8118001
H	-0.9684299	-0.2607006	-2.5097621
H	6.3461821	-0.9263564	-0.4738072
H	3.5119967	4.5902151	0.6356339
C	2.9958062	4.1570419	-1.4135230
H	3.9931019	1.5410679	1.9208379
H	2.2918487	1.7043296	2.3600063
H	3.3799185	3.1309445	2.4423288
H	2.5073284	3.3501249	-3.3544127
H	2.6730267	0.0275192	-2.8670416
H	1.7317514	1.2118942	-3.8118001
H	0.9684299	0.2607006	-2.5097621
H	-6.8142981	3.6089000	-0.6163193
H	-5.8435687	4.5670850	0.5269964
H	-6.9367483	3.2992141	1.1211622
H	6.9367483	-3.2992141	1.1211622
H	6.8142981	-3.6089000	-0.6163193
H	5.8435687	-4.5670850	0.5269964
C	-3.2697597	-5.5497280	-1.9153331
C	3.2697597	5.5497280	-1.9153331
H	-2.3550538	-6.1647367	-1.8815744
H	-3.6160629	-5.5396856	-2.9584894
H	-4.0254504	-6.0571365	-1.2991754
H	4.0254504	6.0571365	-1.2991754
H	2.3550538	6.1647367	-1.8815744
H	3.6160629	5.5396856	-2.9584894

8. References

1. B. L. Tran, M. Pink, D. J. Mindiola, *Organometallics* **2009**, *28*, 2234–2243.
2. M. Bhunia, J. S. Mohar, C. Sandoval-Pauker, D. Fehn, E. S. Yang, M. R. Gau, J. Goicoechea, A. Ozarowski, J. Krzystek, J. Telser, K. Meyer, D. J. Mindiola, *J. Am. Chem. Soc.* **2024**, *146*, 3609–3614.
3. M. Bhunia, M. R. Mena, J. S. Mohar, M. R. Gau, D. J. Mindiola, *J. Am. Chem. Soc.* **2025**, *147*, 2984–2990.
4. F. F. Puschmann, D. Stein, D. Heift, C. Hendriksen, Z. A. Gal, H.-F. Grützmacher, H. Grützmacher, *Angew. Chem. Int. Ed.* **2011**, *50*, 8420–8423.
5. M. Podewitz, J. D. van Beek, M. Wörle, T. Ott, D. Stein, H. Rüegger, B. H. Meier, M. Reiher, H. Grützmacher, *Angew. Chem. Int. Ed.* **2010**, *49*, 7465–7469.
6. A. Hinz, J. M. Goicoechea, *Angew. Chem. Int. Ed.* **2016**, *55*, 8536–8541.

7. S. Yao, Y. Grossheim, A. Kostenko, E. Ballestero-Martínez, S. Schutte, M. Bispinghoff, H. Grützmacher, M. Driess, *Angew. Chem. Int. Ed.* **2017**, *56*, 7465–7469.
8. K. Dollberg, S. Schneider, R. Richter, T. Dunaj, C. Von Hänisch, *Angew. Chem. Int. Ed.* **2022**, *61*, e202213098.
9. R. K. Harris, E. D. Becker, S. M. Cabral De Menezes, R. Goodfellow, P. Granger, *Pure Appl. Chem.* **2001**, *73*, 1795–1818.
10. E. M. Schubert, *J. Chem. Educ.* **1992**, *69*, 62.
11. G. A. Bain, J. F. Berry, *J. Chem. Educ.* **2008**, *85*, 532.
12. CrysAlisPro 1.171. 43.106a: Rigaku Oxford Diffraction, Rigaku Corporation, Oxford, UK. (**2024**).
13. CrysAlisPro 1.171. 43.121a: Rigaku Oxford Diffraction, Rigaku Corporation, Oxford, UK. (**2024**).
14. CrysAlisPro 1.171.43.94a: Rigaku Oxford Diffraction, Rigaku Corporation, Oxford, UK. (**2023**).
15. CrysAlisPro 1.171. 43.106a: Rigaku Oxford Diffraction, Rigaku Corporation, Oxford, UK. (**2024**).
16. CrysAlisPro 1.171. 43.121a: Rigaku Oxford Diffraction, Rigaku Corporation, Oxford, UK. (**2024**).
17. SCALE3 ABSPACK v1.0.7: an Oxford Diffraction program; Oxford Diffraction Ltd: Abingdon, UK, **2005**.
18. SHELXT v2018/2: G. M. Sheldrick, *Acta Cryst.* A **2015**, *71*, 3−8.
19. SHELXL-2019/3: G. M. Sheldrick, *Acta Cryst.* A **2015**, *71*, 3−8.
20. A. L. Spek, *Acta Crystallogr. D Biol. Crystallogr.* **2009**, *65*, 148–155.
21. O. V. Dolomanov, L. J. Bourhis, R. J. Gildea, J. A. K. Howard, H. Puschmann, *J. Appl. Crystallogr.* **2009**, *42*, 339–341.
22. L. N. Grant, B. Pinter, J. Gu, D. J. Mindiola, *J. Am. Chem. Soc.* **2018**, *140*, 17399–17403.
23. G. R. Fulmer, A. J. M. Miller, N. H. Sherden, H. E. Gottlieb, A. Nudelman, B. M. Stoltz, J. E. Bercaw, K. I. Goldberg, *Organometallics* **2010**, *29*, 2176–2179.
24. TURBOMOLE, Version 7.9 2024; a development of University, of Karlsruhe and Forschungszentrum Karlsruhe GmbH 1989-2007, TURBOMOLE GmbH since 2007, available via www.turbomole.org, **2024**.

25. J. P. Perdew, K. Burke, M. Ernzerhof, *Phys. Rev. Lett.* **1996**, *77*, 3865–3868.

26. O. Treutler, R. Ahlrichs, *J. Chem. Phys.* **1995**, *102*, 346–354.

27. F. Weigend, A. Baldes, *J. Chem. Phys.* **2010**, *133*, 174102.

28. A. Pausch, *J. Chem. Theory Comput.* **2024**, *20*, 3169–3183.

29. S. Grimme, S. Ehrlich, L. Goerigk, *J. Comput. Chem.* **2011**, *32*, 1456–1465.

30. J. Pipek, P. G. Mezey, *J. Chem. Phys* **1989**, *90*, 4916–4926.

31. K. B. Wiberg, *Tetrahedron* **1968**, *24*, 1083–1096.

32. Y. J. Franzke, F. Weigend, *J. Chem. Theory Comput.* **2019**, *15*, 1028–1043.

33. Y. J. Franzke, C. Holzer, *J. Chem. Phys.* **2023**, *159*, 184102.

34. P. Pollak, F. Weigend, *J. Chem. Theory Comput.* **2017**, *13*, 3696–3705.

35. J. P. Perdew, M. Ernzerhof, K. Burke, *J. Chem. Phys.* **1996**, *105*, 9982–9985.

36. C. Holzer, *J. Chem. Phys.* **2020**, *153*, 184115.
